# Global, regional, and national burden of congenital musculoskeletal and limb anomalies, 1990–2021: a systematic analysis of the global burden of disease in 2021

**DOI:** 10.1186/s41182-025-00750-4

**Published:** 2025-05-12

**Authors:** Yu Luo, Rubin Zheng, Jiaxi Chen, Miao Deng, Ziyang Zhang, Zhouke Tan, Zhixun Bai

**Affiliations:** 1 Department of Pediatric Surgery, Affiliated Hospital of Zunyi Medical University, Zunyi, 563006 China; 2 Department of Nephrology, People’s Hospital of Qianxinan Prefecture, Xingyi, 562400 Guizhou China; 3 Clinical College, Zunyi Medical University, Zunyi, 563000 Guizhou China; 4 Department of Nephrology, Affiliated Hospital of Zunyi Medical University, Zunyi, 563006 Guizhou China; 5 Organ Transplant Center, Affiliated Hospital of Zunyi Medical University, Zunyi, 563006 Guizhou China

**Keywords:** Congenital musculoskeletal and limb anomalies, Incidence, Mortality, Prevalence, Global burden of disease

## Abstract

**Background:**

Congenital musculoskeletal and limb (CML) anomalies are uncommon, multifactorial conditions whose global incidence trends remain underexplored. This study delineates the epidemiology and temporal evolution of CML anomalies from 1990 to 2021.

**Methods:**

We extracted data from the 2021 global burden of disease (GBD) Study, stratifying by sex, region, country and socio-demographic index (SDI). We calculated age-standardized incidence rate (ASIR), age-standardized mortality rate (ASMR), age-standardized prevalence rate (ASPR), and estimated annual percentage change (EAPC). Decomposition analysis quantified the contributions of population growth, aging, and epidemiological change. Projections to 2031 were made using an autoregressive integrated moving average (ARIMA) model. Health inequities were assessed via the slope index of inequality (SII) and concentration index (CI).

**Results:**

Global epidemiological patterns of CML anomalies exhibited significant disparities between 1990 and 2021. Brunei Darussalam demonstrated the highest ASIR, while Afghanistan and the United Mexican States recorded the highest ASMR and ASPR, respectively. Absolute case and death burdens predominantly clustered in populous nations, with India and China reporting the highest absolute numbers. ARIMA modeling projected a 0.85% increase in incident cases (from 2,437,890.12 to 2,458,596.45), a 25.46% decrease in mortality (from 13,599.83 to 10,137.02), and a 3.55% increase in prevalence (from 18,549,408.27 to 19,207,414.19) by 2031. Decomposition analyses revealed that population growth was the primary driver of increased cases in middle SDI regions, whereas epidemiological transitions and aging were the main contributors to mortality reductions. In lower-middle SDI regions, concurrent demographic expansion and epidemiological changes amplified case burdens. Health inequality significantly increased, with the incidence CI rising from 0.28 to 0.35 and the mortality CI from 0.34 to 0.42 between 1990 and 2021. Significant correlations were observed between EAPC and baseline ASIR/ASMR, with declining trends in mortality and rising prevalence driven by population growth and epidemiological transitions.

**Conclusion:**

From 1990 to 2021, CML anomalies’ incidence and mortality exhibited divergent trends across SDI strata, with less favorable outcomes in lower-SDI countries. Tailored interventions are essential to mitigate the growing burden in these settings.

**Supplementary Information:**

The online version contains supplementary material available at 10.1186/s41182-025-00750-4.

## Introduction

Congenital musculoskeletal and limb (CML) anomalies constitute a group of birth defects that are prevalent globally, significantly impacting children’s health and quality of life. The global burden of these conditions has demonstrated complex temporal trends, influenced by population growth and advancements in medical technology. The World Health Organization (WHO) estimates that congenital anomalies are among the primary causes of neonatal mortality and long-term disability in children [1].

The precise prevalence of CML anomalies remains to be fully elucidated, exhibiting significant variations across geographical regions, ethnic groups, and socioeconomic strata [2]. It is estimated that 6% of global infant deaths are attributable to congenital anomalies, with 92% of these deaths occurring in low- and middle-income countries [3]. Furthermore, several factors have been identified as significantly associated with the development of CML anomalies, including genetic factors, environmental exposures, and maternal health [4–6]. Recent years have witnessed an increasing number of studies focusing on the role of genetics in CML anomalies, largely due to advancements in molecular biology techniques [7–9]. A study conducted in Pakistan revealed that familial factors were implicated in 35% of cases of congenital malformations [10]. However, there remains a paucity of in-depth understanding of the etiology and pathogenesis of many complex malformations, which limits the development of effective preventive and intervention strategies. CML anomalies have a considerable impact on affected individuals and their families. These deformities can result in significant physical dysfunction, impacting the patient’s capacity for physical activity, daily living, and psychological well-being. On the other hand, the long-term medical needs and social support required can impose a substantial financial burden on families and society [2]. Therefore, it is imperative to comprehend the global epidemiological characteristics and temporal trends of these conditions to formulate targeted prevention strategies.

The global burden of disease (GBD) study provides detailed data on CML anomalies, offering a valuable opportunity to assess their health impacts at global, national, and regional levels. Using the GBD database, this study systematically analyzed the temporal trends of incidence, mortality, and prevalence of CML anomalies from 1990 to 2021, and further explored their regional variations. The findings of this study are expected to contribute to the existing literature and provide a scientific basis for the development of targeted prevention strategies for congenital anomalies in diverse countries and regions.

## Materials and methods

### Study data

This observational study constitutes a time-series cross-sectional analysis using data from the GBD 2021 study. Metrics for CML anomalies—including age-standardized incidence rates (ASIR), age-standardized mortality rates (ASMR), age-standardized prevalence rates (ASPR), and absolute counts of cases and deaths—were obtained from 1990 to 2021 via the Global Health Data Exchange (GHDx) query tool (http://ghdx.healthdata.org/gbd-results-tool). The Socio-demographic Index (SDI), a composite measure of national development, was calculated as the geometric mean of three normalized components (0.00–1.00 scale): lag-distributed income per capita (log-transformed, PPP-adjusted), average years of education in populations aged ≥15 years, and total fertility rate in females <25 years. Countries were stratified into five SDI quintiles in 2021: low (0.00–0.45), low-middle (0.46–0.61), middle (0.62–0.69), high-middle (0.70–0.80), and high (≥0.81) [11, 12]. To account for geographical heterogeneity, the cohort was further divided into 21 regions (e.g., Sub-Saharan Africa, South Asia) following GBD protocols. In the GBD database, CML anomalies are classified under"Non-communicable diseases", specifically within"Other non-communicable diseases", under the"Congenital birth defects"category at the level 4 directory. CML anomalies are defined as abnormalities in the muscles or skeletal system present at birth that are not caused by a defined chromosomal syndrome. These anomalies are further divided into three subcategories: polydactyly and syndactyly, limb reduction defects, and all other CML anomalies. Polydactyly and syndactyly correspond to ICD-10 codes Q69–Q70, limb reduction defects to Q71–Q73, and other CML anomalies to Q65–Q68 and Q74–Q79. Historical ICD-9 codes (754–756) were mapped to ICD-10 equivalents using GBD crosswalk algorithms [12]. All aggregated data, including age-standardized rates computed using the GBD reference population, are publicly accessible through GHDx, ensuring methodological transparency and reproducibility. For detailed modeling procedures and uncertainty adjustments, see the GBD 2021 Modeling Framework section below.

### GBD 2021 modeling framework

CML anomalies were generated through the standardized GBD 2021 analytical pipeline, integrating 60,902 data sources across 204 countries from 1990 to 2021. In GBD 2021, the estimation of congenital musculoskeletal and limb abnormalities was based on data from multiple international birth defect registries (e.g., EUROCAT), inpatient records, and systematic literature reviews [13]. We synthesized data from congenital anomaly registries, ICD-10-coded hospitalization records (Q65–Q79), nationally representative surveys (e.g., Demographic and Health Surveys), and systematic literature reviews. For regions with sparse data (e.g., sub-Saharan Africa), spatiotemporal Gaussian process regression (ST-GPR) with covariates (SDI, Healthcare Access and Quality Index [HAQI]) imputed missing values, while ambiguous ICD codes (e.g., Q79.9) were reclassified probabilistically using clinical consensus surveys (n = 142) [12].

To ensure temporal consistency, pre-2000 data were adjusted for under ascertainment of non-lethal anomalies, reflecting limited prenatal imaging capabilities during that period. Post-2000 estimates incorporated an annual 12% improvement in detection probability per unit increase in the HAQI, reflecting advancements in diagnostic infrastructure. Mortality was modeled using SDI-dependent logistic functions, where survival probabilities exhibited a non-linear increase when SDI exceeded 0.7, reflecting improved surgical accessibility [14]. Both SDI and HAQI were modeled as linear predictors with spline terms at key development thresholds (knots at SDI = 0.3, 0.6, 0.8) to account for heterogeneous healthcare capacity effects. Statistical coherence across incidence, prevalence, and mortality estimates was enforced using DisMod-MR 2.1, a Bayesian meta-regression tool incorporating cause-specific mortality rates (CSMR), 12 covariates (e.g., SDI, folate deficiency proxies), and Bayesian regression adjustments to improve accuracy and reproducibility. The model assumes that congenital anomalies occur exclusively at birth, with partial resolution possible through surgical interventions during childhood [13]. Spatiotemporal extrapolation utilized Gaussian process regression with a Matérn kernel (ν = 2.5), weighted by geographical adjacency and SDI concordance [12].

### Model robustness and uncertainty analysis

Model robustness was validated through cross-validation and ensemble simulations (see"Uncertainty quantification"section). Probabilistic uncertainty analysis combined Bayesian posterior estimation via Markov chain Monte Carlo (MCMC) sampling (1000 draws) with multi-model ensemble simulations to quantify parametric and structural uncertainties [15, 16]. The structural uncertainty assessment integrated counterfactual healthcare access scenarios (e.g., a substantial decrease in surgical capacity in regions with SDI < 0.5) to evaluate the policy-sensitive determinants of mortality trends [17, 18]. Systematic data quality corrections reduced errors in passive surveillance systems by over 20%, aligning with GBD best practices.

### Statistical analysis

The present study employed ASIR, ASMR, ASPR, and Estimated Annual Percentage Change (EAPC) to quantify the burden of CML anomalies. Age standardization is essential when comparing populations with differing age structures or when examining changes in age distribution within the same population over time. The Age-Standardized Rate (ASR), expressed per 100,000 population, was calculated using the direct method. This method involves summing the products of age-specific rates (a_i_, where i denotes the i^th^ age class) and the corresponding number of persons (or weight) (w_i_) in the same age subgroup i of the chosen reference standard population. The sum is then divided by the total sum of the standard population weights, i.e.,

$$ASR=\frac{{\sum }_{\left\{i=1\right\}}^{A}{a}_{i}{w}_{i}}{{\sum }_{\left\{i=1\right\}}^{A}{w}_{i}}\times \text{100,000}$$

For ASIR, ASMR, and ASPR, Bayesian uncertainty intervals (UIs) were derived via 1,000 MCMC posterior draws, reflecting probabilistic parameter distributions. In contrast, frequentist confidence intervals (CIs) were exclusively applied to EAPC trends, calculated through log-linear regression models.

UIs for age-standardized rates were derived via Bayesian posterior estimation (see"Uncertainty quantification"section for details). This approach accounts for variability from three key sources: data measurement errors (e.g., underreporting in passive surveillance systems, particularly in low SDI regions), model parameter uncertainty (e.g., stochastic variability in regression coefficients and covariate effects), and structural uncertainty (e.g., imputation for sparse data) [12]. The final UI represents the 2.5 th and 97.5 th percentiles of the posterior distribution. Temporal changes (e.g., 1990 vs. 2021) were deemed significant if the UI of the absolute difference excluded zero [14].

The Estimated Annual Percentage Change (EAPC) quantifies the temporal trend in age-standardized rates (ASR) by fitting a log-linear regression model to the natural logarithm of annual rates: y = α + βx + ϵ, where *y* = ln (ASR) and x = calendar year. The EAPC is calculated as 100 × (exp(β) − 1), representing the average annual percentage change. The 95% confidence interval (CI) was derived from the variance–covariance matrix of regression coefficients, using the normal approximation method: 95% CI = [100 × (exp(β − 1.96 × SE(β)) − 1), 100 × (exp(β + 1.96 × SE(β)) − 1)]. Trends were classified as increasing (EAPC and lower CI > 0), decreasing (EAPC and upper CI < 0), or *stable* (CI spanning zero), ensuring distinction between genuine epidemiological shifts and random variability. This approach distinguishes genuine epidemiological shifts from random variability, ensuring robust trend interpretation [2, 19].

To quantify uncertainty, 95% UIs for ASIR, ASMR, and ASPR were derived through 1,000 MCMC posterior draws, accounting for data measurement errors and model parameter variability. The 2.5 th and 97.5 th percentiles of the posterior distribution defined the 95% UI, with wider intervals indicating higher uncertainty in data-sparse regions (e.g., low SDI countries) and narrower intervals reflecting precise estimates in well-surveyed populations [12]. Model robustness was validated via tenfold cross-validation, yielding an RMSE of 1.2 per 100,000 for incidence rates [12]. If both the EAPC estimate and the lower bound of its 95% CI are greater than 0, the ASR is considered to be on an increasing trend. Conversely, if both the EAPC estimate and the upper bound of its 95% CI are less than 0, the ASR is on a decreasing trend. In all other cases, the ASR is considered stable over time. To explore factors influencing the EAPC, this study assessed the association between EAPC and ASR at the national level. Decomposition analysis was used to visualize the contributions of three factors driving changes in the number of morbidities and deaths related to CML anomalies between 1990 and 2021. This analysis examined the roles of aging, population growth, and epidemiological changes in driving these trends. Epidemiological changes refer to underlying age- and population-adjusted mortality and morbidity rates [20].

### Autoregressive integrated moving average model

ARIMA is comprised of two constituent models: the autoregressive (AR) model and the moving average (MA) model. The fundamental assumption of the model is that the data series are time-dependent random variables whose autocorrelation can be characterized by the ARIMA model. The latter can predict future values based on past values. The equation can be expressed as follows: Y_t_ = φ_1_Y_t-1_ + φ_2_Y_t-2_ + … + φ_p_Y_t-p_ + e_t_ − θ_1_e_t-1_ − … − θ_q_e_t-q_. The AR model part is represented by the following equation:

where (φ_1_Y_t-1_ + φ_2_Y_t-2_ + … + φ_p_Y_t-p_ + e_t_) is the AR model part, (e_t_ − θ_1_e_t-1_ − … − θ_q_e_t-q_) is the MA model part, Y_t-1_ is the observation of (t-p) period, p and q denote AR and MA, respectively, and e_t_ is the t-period random error [21]. The time series in the ARIMA model is required to be a stationary random series with a zero mean. Stationarity was confirmed through augmented Dickey-Fuller tests (*p* < 0.01), and residual diagnostics (Ljung-Box test: *p* > 0.05) validated the absence of autocorrelation [22, 23].

### Cross-country inequalities analysis

The Slope Index of Inequality (SII) and the Concentration Index (CI) are standardized measures of absolute and relative gradient imbalances, respectively. The SII is calculated using regression analysis, relating a country’s ASIR or ASMR to its relative position on the SDI, as defined by the midpoint of the population in the cumulative distribution sorted by SDI [24]. A weighted regression model was used to address potential heteroscedasticity. National SDI ranks were derived from lag-distributed income, education, and fertility metrics [12]. CIs were calculated by numerically integrating the area under the Lorenz curve, with the cumulative proportions of ASIR, ASMR, and ASPR aligned with the cumulative distribution of the population sorted by SDI [25]. The SII quantified absolute disparities by regressing ASIR/ASMR against national SDI ranks, while the CI measured relative inequality by comparing cumulative disease burden against cumulative population shares. Cross-country inequality trends were validated against historical GBD estimates to ensure methodological consistency [12].

### Uncertainty quantification

UIs represent Bayesian posterior distributions, while CIs are derived from frequentist regression models. Uncertainty in estimates was quantified through a comprehensive approach. UIs were generated using 1000 posterior draws via Bayesian MCMC sampling. The 95% UI represents the 2.5 th and 97.5 th percentiles of the posterior distribution, incorporating variability from three primary sources: data measurement errors (e.g., underreporting in passive surveillance systems, particularly in low SDI regions), model parameter uncertainty (e.g., stochastic variability in regression coefficients and covariate effects), and structural uncertainty (e.g., imputation for sparse data or counterfactual healthcare scenarios) [26, 27]. This approach systematically integrates variability across measurement, modeling, and structural assumptions to provide a robust assessment of uncertainty.

Temporal changes (e.g., 1990 vs. 2021 comparisons) were deemed significant if the 95% UI of the absolute difference excluded zero. Similarly, cross-group differences (e.g., between SDI quintiles) were considered significant if their UIs did not overlap. The width of the UI reflects the precision of estimates, with narrower intervals (e.g., in high SDI regions) indicating higher confidence in data infrastructure, while wider intervals (e.g., sub-Saharan Africa) highlight systemic limitations in congenital anomaly surveillance [12].

To address structural uncertainty, counterfactual analyses were conducted. For instance, reducing surgical capacity by 50% in SDI < 0.5 regions revealed that mortality trends were highly sensitive to healthcare access policies. Model robustness was further validated through tenfold cross-validation (incidence RMSE: 1.2/100,000) and multi-model ensemble simulations to capture parametric and structural uncertainties [14].

### Methodological limitations

Three limitations inherent to the GBD framework should be noted. First, sparse data coverage in low SDI regions introduces residual uncertainties despite hierarchical Bayesian imputation, primarily due to fragmented vital registration systems and underdiagnosis of non-lethal anomalies [16].

Additionally, the interpretation of UIs requires careful consideration. Bayesian UIs and frequentist CIs reflect distinct statistical paradigms: UIs represent posterior probability distributions conditioned on prior assumptions, whereas CIs rely on repeated sampling frameworks. In data-sparse regions, Bayesian UIs may overestimate uncertainty if non-informative priors are used, while frequentist CIs might underestimate variability due to model misspecification [28].

Second, the ARIMA model’s reliance on historical trends constrains its sensitivity to sudden disruptions, such as healthcare fragmentation during the COVID-19 pandemic, potentially affecting post-2021 projection accuracy. Furthermore, ARIMA’s assumption of linear temporal patterns may fail to capture complex interactions between demographic shifts and epidemiological transitions, particularly in rapidly developing regions [29].

Finally, unquantified mediation effects (e.g., environmental toxin exposure via maternal health pathways) may bias attributable burden estimates [30]. Although uncertainty quantification incorporated MCMC and regression techniques, the conceptual divergence between Bayesian UIs and frequentist CIs necessitates clarification. Bayesian UIs reflect posterior distributions integrating prior knowledge and observed data, while frequentist CIs are grounded in hypothetical long-run frequency properties; these differences may lead to divergent interpretations in settings with sparse data or weakly identified models [28]. Furthermore, our models did not adjust for unmeasured confounders, including epigenetic regulation of teratogen susceptibility or gene-environment interplay, which may partially explain residual heterogeneity in risk estimates [31]. To address these limitations, subsequent research could implement sensitivity analyses, such as nonparametric bootstrapping or prior robustness checks, to evaluate the stability of interval estimates across statistical frameworks.

Statistical analyses were conducted using R version 4.3.0, and a *p*-value less than 0.05 indicated statistical significance.

## Result

### Global burden of CML anomalies

**Fig. 1:**
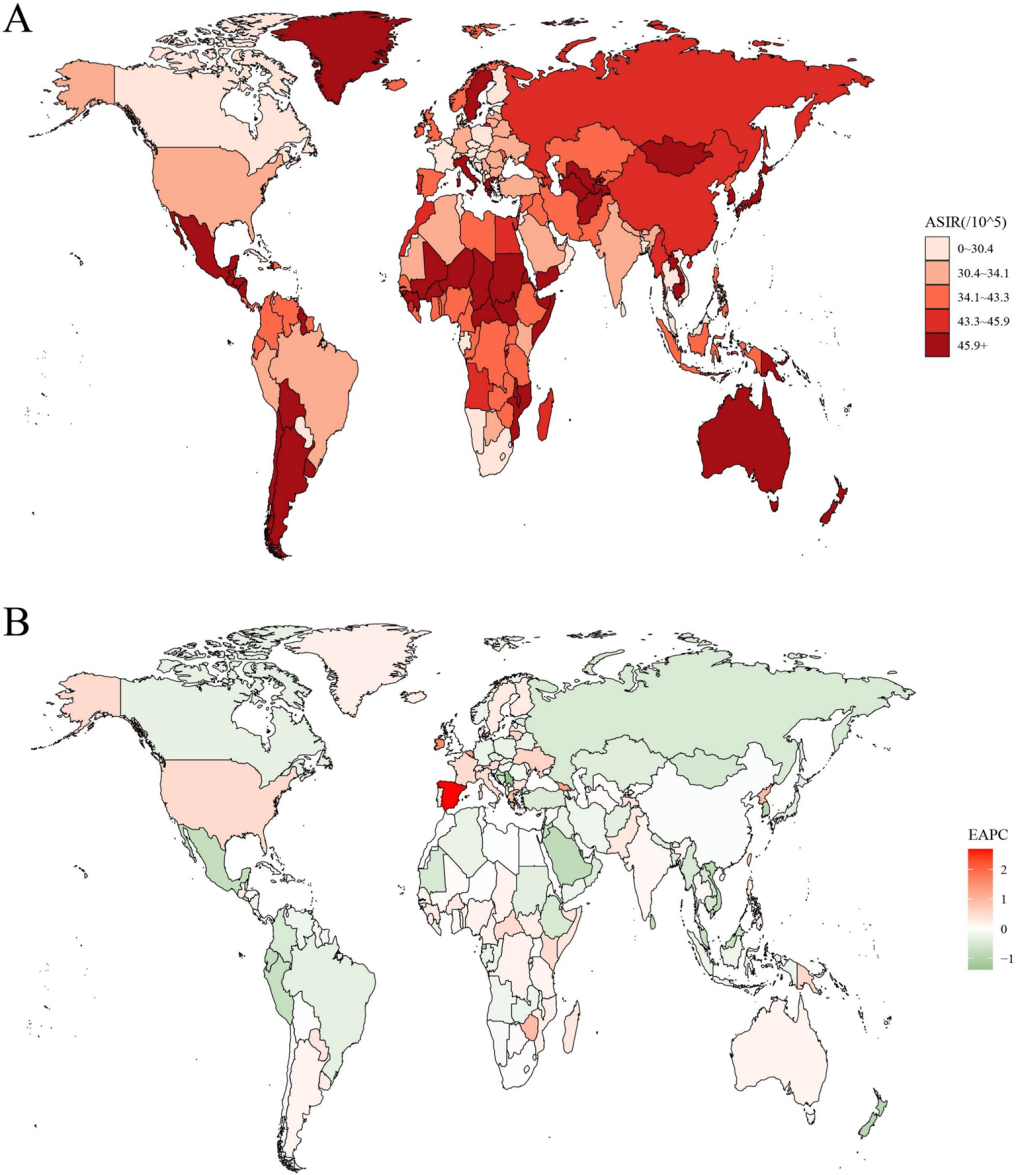
The global disease burden of CML anomalies for both sexes in 204 countries and territories. **A** The ASIR of CML anomalies in 2021; **B** The EAPC of CML anomalies ASIR from 1990 to 2021. *CML* congenital musculoskeletal and limb, *ASIR* age-standardized incidence rate, *EAPC* estimated annual percentage change

ASIR, ASMR and ASPR for CML anomalies exhibited substantial global variation (Fig. 1; Supplementary Figures S1 and S2). In 2021, Brunei Darussalam reported the highest ASIR (76.70 per 100,000; 95% uncertainty interval [UI]: 53.30–109.40), followed by Guatemala (75.60 per 100,000; 95% UI: 50.50–108.60) and Argentina (73.30 per 100,000; 95% UI: 51.70–102.80). In terms of absolute numbers, India recorded the highest incidence of CML anomalies in 2021 (339,702.30; 95% UI: 239,365.50–470,274.40), followed by China (238,561.20; 95% UI: 169,545.20–338,146.80) and Nigeria (170,560.30; 95% UI: 117,846.10–242,681.10) (Supplementary Table S1). In contrast, Afghanistan had the highest ASMR (0.90 per 100,000; 95% UI: 0.30–1.60), followed by Dominica, Sudan, and Yemen (0.70 per 100,000; 95% UI: 0.50–1.00). In absolute numbers, India reported the highest number of CML anomaly deaths in 2021 (2,421.30; 95% UI: 1430.70–4255.90), followed by Nigeria (1,371.00; 95% UI: 857.50–2,118.40) and Pakistan (689.10; 95% UI: 346.80–1524.70) (Supplementary Table S2). In 2021, Mexico had the highest ASPR (492.90 per 100,000; 95% UI: 394.40–605.90), followed by Japan (488.30 per 100,000; 95% UI: 394.40–605.90) and Greece (474.20 per 100,000; 95% UI: 394.40–605.90). In absolute numbers, China reported the highest number of prevalent cases (3,112,683.70; 95% UI: 2,565,594.20–3,820,090.20), followed by India (2,771,851.20; 95% UI: 2,565,594.20–3,820,090.20) and Indonesia (639,780.70; 95% UI: 514,381.60–783,909.00) (Supplementary Table S3). All ASIR, ASMR, and ASPR estimates are reported with 95% UIs (Bayesian posterior intervals), while EAPC trends are accompanied by 95% CIs (frequentist intervals). For example, Brunei’s ASIR UI (53.30–109.40) implies a 95% probability that the true incidence lies within this range, whereas EAPC CIs determine trend directionality (e.g., a CI lower bound > 0 indicates an increasing trend). UI width directly correlates with estimation precision, while CI overlaps with zero dictates statistical significance.

**Fig. 2:**
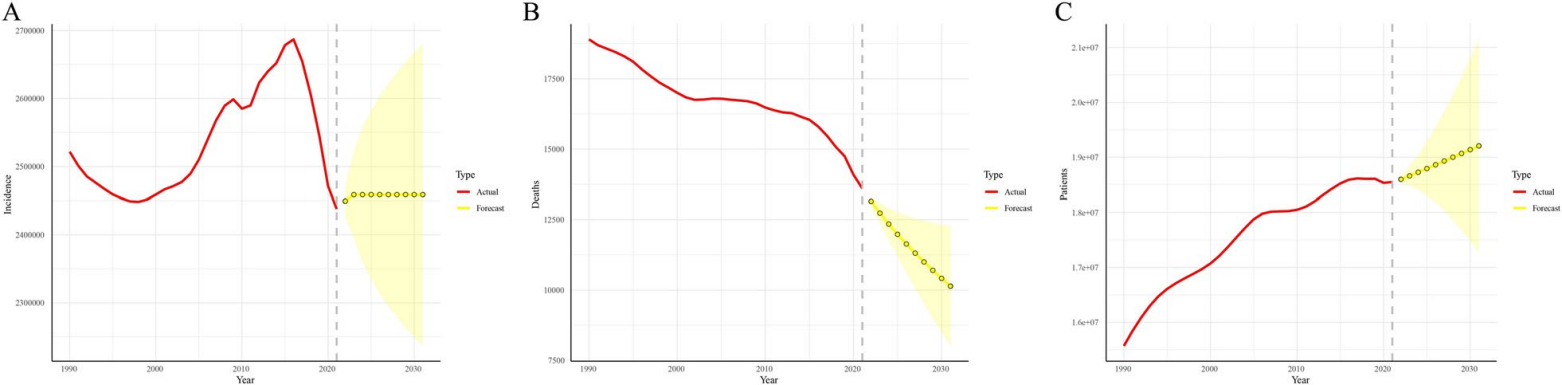
Projected trends of incident cases (**A**), deaths (**B**), and prevalence (**C**) of congenital musculoskeletal and limb anomalies in the next decade (2021–2031). *Red lines* represent the true trend during 1990–2021; *yellow dotted lines* and shaded regions represent the predicted trend and its 95% Bayesian UIs

The ARIMA model projections indicated that the number of incident cases of CML anomalies was expected to increase from 2,437,890.12 in 2021 to 2,449,310.78 (95% uncertainty interval [UI]: 2,427,493.50–2,471,128.00) in 2022. Subsequently, incident cases were predicted to remain relatively stable from 2023 to 2031, indicating a stable trend in overall incidence. In contrast, the model predicted a decreasing trend in the number of deaths from CML anomalies, declining from 13,599.83 in 2021 to 10,137.02 (95% UI: 8013.71–12,260.34) in 2031. Conversely, the number of prevalent cases of CML anomalies was projected to continue increasing from 18,549,408.27 in 2021 to 19,207,414.19 (95% UI: 17,267,321.34–21,147,507.04) in 2031 (Fig. 2; Supplementary Table S4).

### Decomposition analysis of age-standardized incidence number, age-standardized death number, and age-standardized prevalence number

**Fig. 3:**
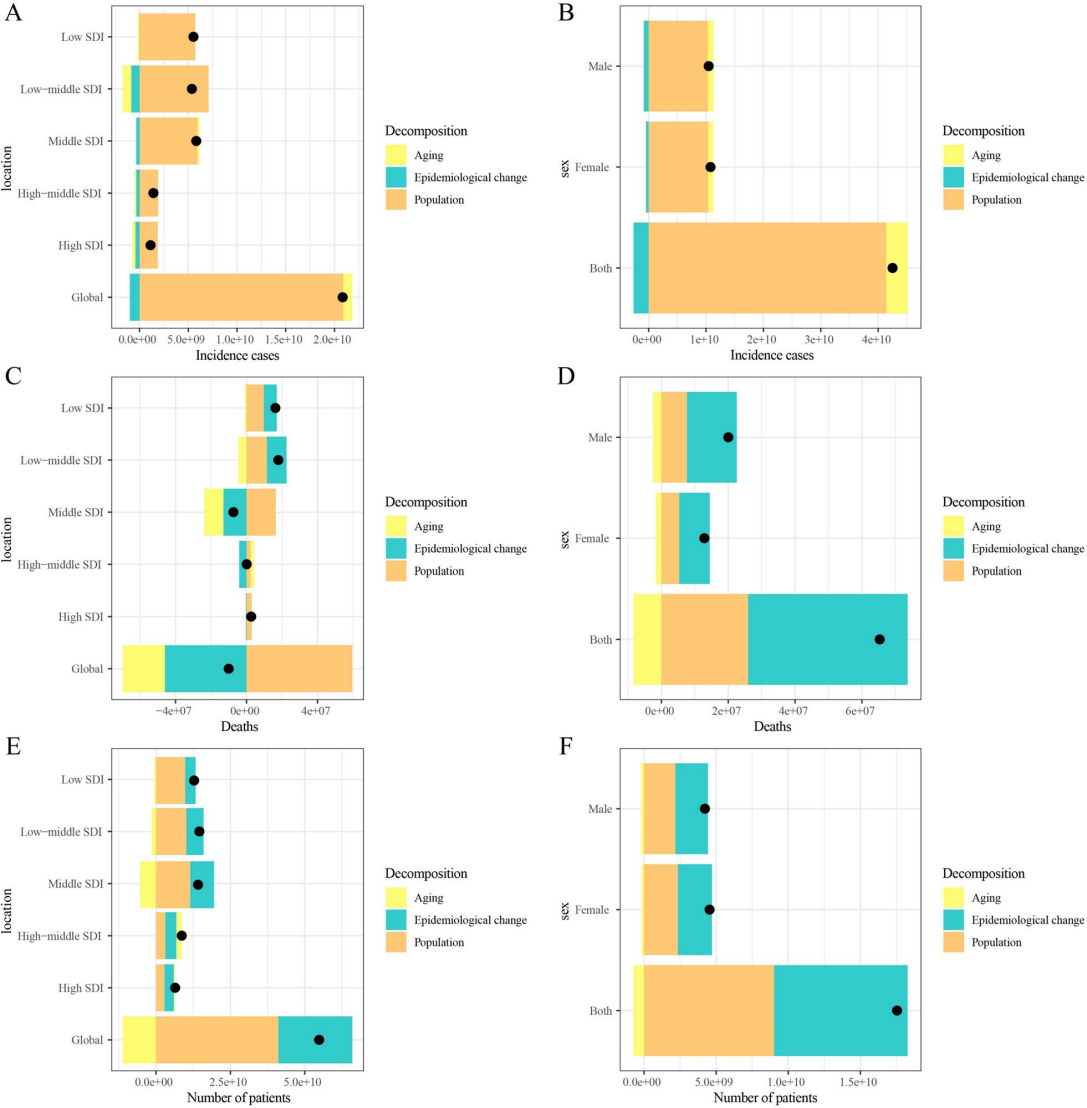
Changes in incidence, mortality and prevalence of CML anomalies from 1990 to 2021 according to population-level determinants of population growth, aging, and epidemiological change across different SDI quintiles and by sex. **A** Decomposition of CML anomalies incidence by SDI quintile; **B** decomposition of CML anomalies incidence by sex; **C** decomposition of CML anomalies mortality by SDI quintile; **D** decomposition of CML anomalies mortality by sex. **E** decomposition of CML anomalies prevalence by SDI quintile; **F** decomposition of CML anomalies prevalence by sex. The *black dot* represents the overall value of change contributed by all three components. *CML* congenital musculoskeletal and limb; *SDI* socio-demographic index

From 1990 to 2021, the observed increase in the global incidence of CML anomalies was primarily influenced by population growth (100.27%). The middle SDI quintile region exhibited the most significant increase in CML anomalies. In this region, population growth accounted for the entire increase (131.86%) (Fig. 3A; Supplementary Table S5). A similar pattern was observed in the overall population, where population growth (97.41%) accounted for the majority of the increase, while aging contributed only 8.77% (Fig. 3B; Supplementary Table S5). Conversely, the global decline in the number of deaths due to CML anomalies was primarily attributed to epidemiological changes (−456.53%), followed by aging (−235.66%). Population growth also influenced CML anomaly mortality (592.20%). The middle SDI quintile exhibited the most significant decline in deaths attributable to CML anomalies, with epidemiological changes contributing the most (−175.92%), followed by aging (−147.02%) (Fig. 3C; Supplementary Table S5). However, when stratified by sex, the decrease in the number of deaths due to CML anomalies was primarily influenced by aging (−12.77%) (Fig. 3D; Supplementary Table S5).

The global increase in the number of CML anomalies was primarily driven by population growth (75.01%), followed by epidemiological changes (45.33%). CML anomalies exhibited the most significant increase in the number of prevalent cases, which was predominantly attributable to population growth (69.98%). Epidemiological changes accounted for 39.68% of this increase. When stratified by sex, the increase in the number of prevalent cases of CML anomalies was primarily influenced by both population growth and epidemiological changes, with respective contributions of 51.40 and 52.76%.

### Factors influencing EAPC

**Fig. 4:**
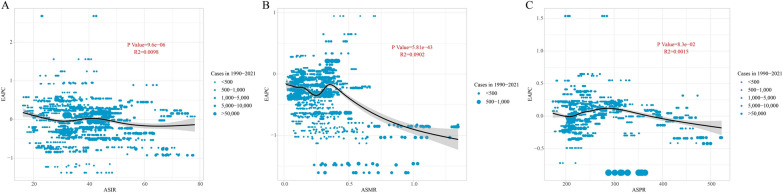
The correlation between EAPC and **A** ASIR, **B** ASMR, and **C** ASPR for CML anomalies. Circles represent CML anomaly cases from 1990 to 2021, with larger circles indicating a higher number of cases. The R^2^ and *p* values were derived from Pearson’s correlation analysis. *EAPC* estimated annual percentage change, *ASIR* age-standardized incidence rates, *ASMR* age-standardized mortality rates, *ASPR* age-standardized prevalence rates, *CML* congenital musculoskeletal and limb

Global trends revealed a significant correlation between EAPC and both ASIR and ASMR (Fig. 1B). Further analysis demonstrated that a significant correlation was observed between EAPC and ASIR, as well as ASMR (*p* < 0.05). The EAPC demonstrated a downward trend, transitioning from positive to negative values as the ASIR fell below 30 per 100,000. Conversely, as the ASIR exceeded 40 per 100,000, the EAPC generally decreased, demonstrating a non-linear relationship (*p* = 9.6E−06, R^2^ = 0.0098). When ASMR < 0.35, the EAPC demonstrates signs of volatility, initially decreasing and subsequently increasing. Concurrently, as the ASMR increased, the EAPC consistently exhibited a downward trajectory (*p* = 5.81E−43, R^2^ = 0.0902). Conversely, no significant correlation was observed between EAPC and ASPR (*p* = 8.3E−02, R^2^ = 0.0015) (Fig. 4).

**Fig. 5:**
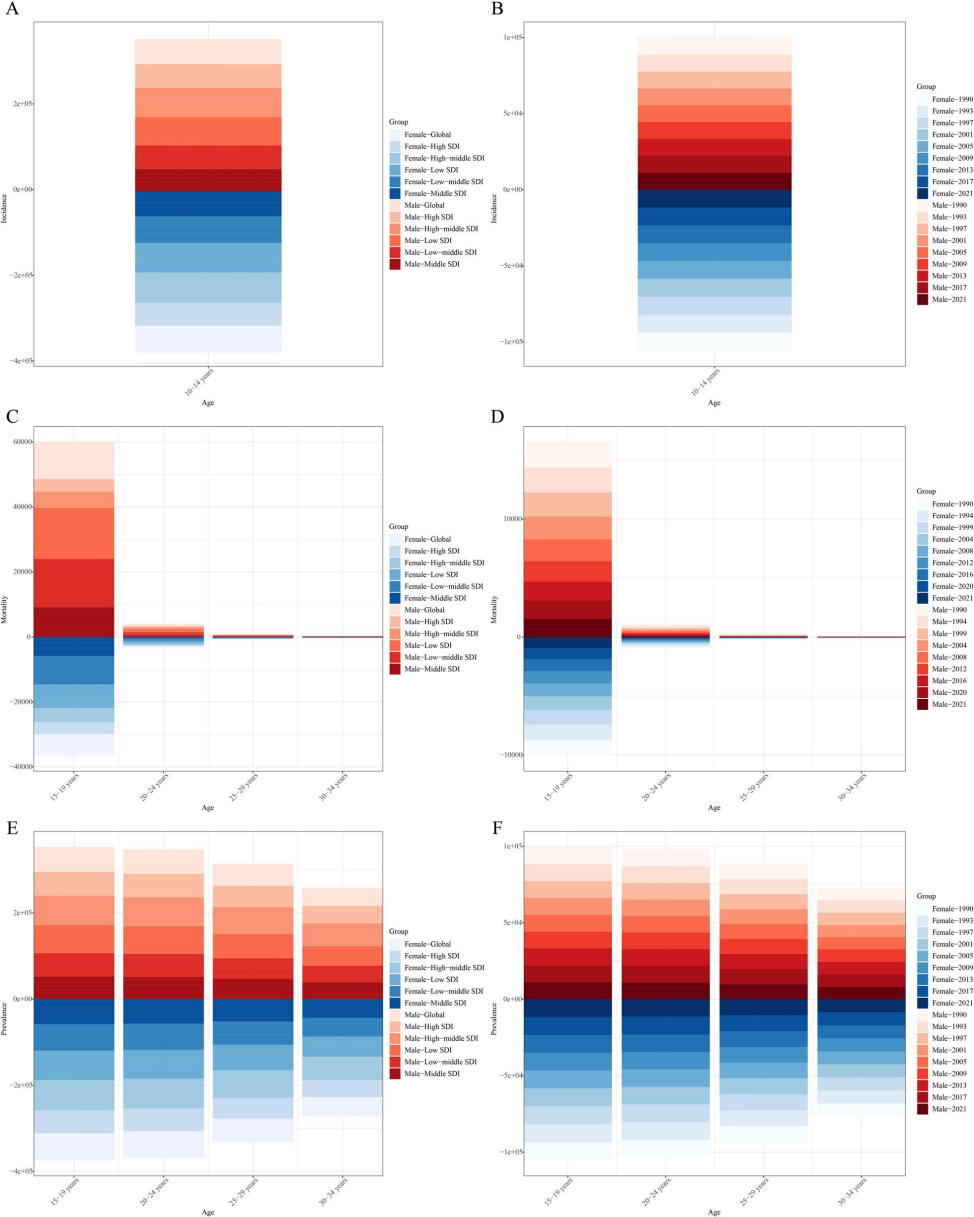
Age-specific incidence (**A**, **B**), mortality (**C**, **D**), and prevalence(**E**, **F**) of congenital musculoskeletal and limb anomalies stratified by sex, SDI levels, and periods. *SDI* the socio-demographic index

The overall incidence of CML anomalies was higher in females than in males (Fig. 5A). Furthermore, the incidence of CML anomalies remained stable across four-year intervals from 1990 to 2021 (Fig. 5B). Conversely, among individuals with CML anomalies of all ages, mortality was higher in males than in females and was concentrated in low and low-middle SDI regions (Fig. 5C). The mortality rate of CML anomalies decreased over time (Fig. 5D). The prevalence of CML anomalies decreased with increasing age. The burden of CML anomalies was more significant in low and high-middle SDI regions (Fig. 5E), and the prevalence remained stable across time periods (Fig. 5F).

### Global health inequality analysis of incidence, mortality and prevalence of CML anomalies from 1990 to 2021

**Fig. 6:**
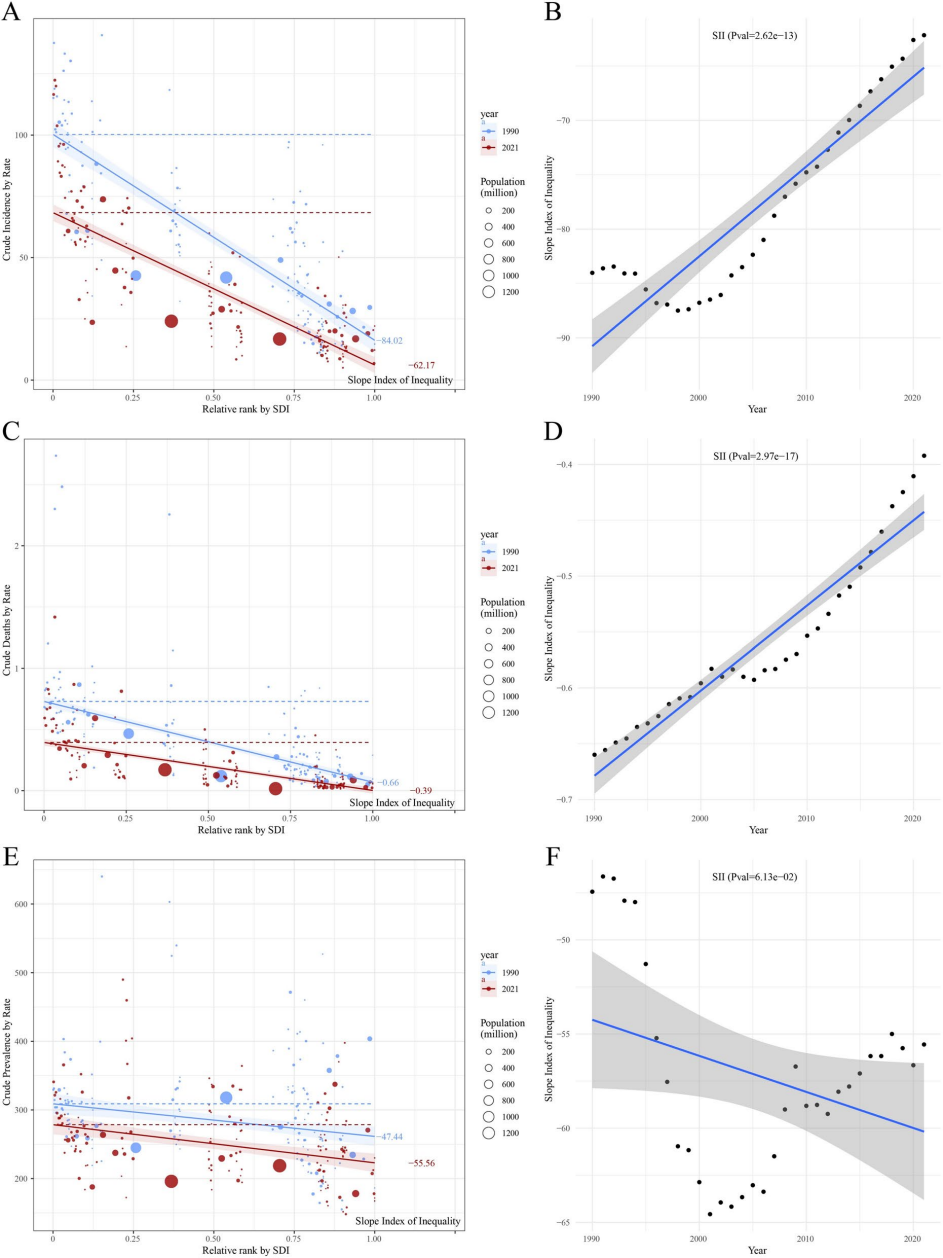
Trends in crude incidence, mortality, and prevalence of CML anomalies by SDI and SII from 1990 to 2021. **A** Crude CML anomalies incidence rate by relative SDI rank, with SII for 1990 and 2021; **B** change in SII over time for crude CML anomalies incidence; **C** crude CML anomalies mortality rate by relative SDI rank, with the SII for 1990 and 2021; **D** change in SII over time for crude CML anomalies mortality; **E** Crude CML anomalies prevalence rate by relative SDI rank, with SII for 1990 and 2021; **F** change in SII over time for crude CML anomalies prevalence. Different circles represent different countries and territories, and the circle size corresponds to the population size. *CML* congenital musculoskeletal and limb, *SDI* the socio-demographic index, *SII* the slope index of Inequality

A reduction in the disparity of incidence and mortality of CML anomalies was observed across different SDI levels between countries and regions in 2021 compared to 1990. The analysis revealed an overall trend of decreasing morbidity rates with increasing SDI levels. SII represents absolute disparities in ASIR across SDI ranks, decreased from 84.02 (95% CI: 75.45–92.60) in 1990 to 62.17 (95% CI: 55.32–69.01) in 2021, indicating reduced inequality. Notably, the negative SII for mortality (−0.39 in 2021) further reflects higher ASMR in lower SDI regions. In 2021, the SII values for incidence were significantly lower than those in 1990, with respective values of 62.17 (95% CI: 55.32–69.01) and 84.02 (95% CI: 75.45–92.60) (Fig. 6A; Supplementary Table S6). Furthermore, the SII values exhibited a downward trend, as evidenced by highly statistically significant regression fitting results (*p* = 2.62e−13), suggesting that inequalities in the incidence of CML anomalies have been decreasing over the past few decades in countries and regions with different levels of social development, and the trend has been very stable (Fig. 6B). A similar decline was observed in the SII values for mortality from CML anomalies in 2021, with values of −0.39 (95% CI: −0.45 to 0.34) and −0.66 (95% CI: −0.740.58), respectively (Fig. 6C; Supplementary Table S6). The regression fits also showed highly statistically significant results (*p* = 2.97e−17). The data demonstrated a decline in the disparity of mortality rates for CML anomalies across different levels of social development between countries and regions over recent decades (Fig. 6D). Conversely, there was an increase in the inequality of the prevalence of CML anomalies, with the SII value decreasing from −47.44 (95% CI: −74.91 to 19.97) in 1990 to −55.56 (95% CI: −79.86 to 31.25) in 2021 (Fig. 6E; Supplementary Table S6). The regression fit for SII was not statistically significant (*p* = 6.13e−02) (Fig. 6F).

**Fig. 7:**
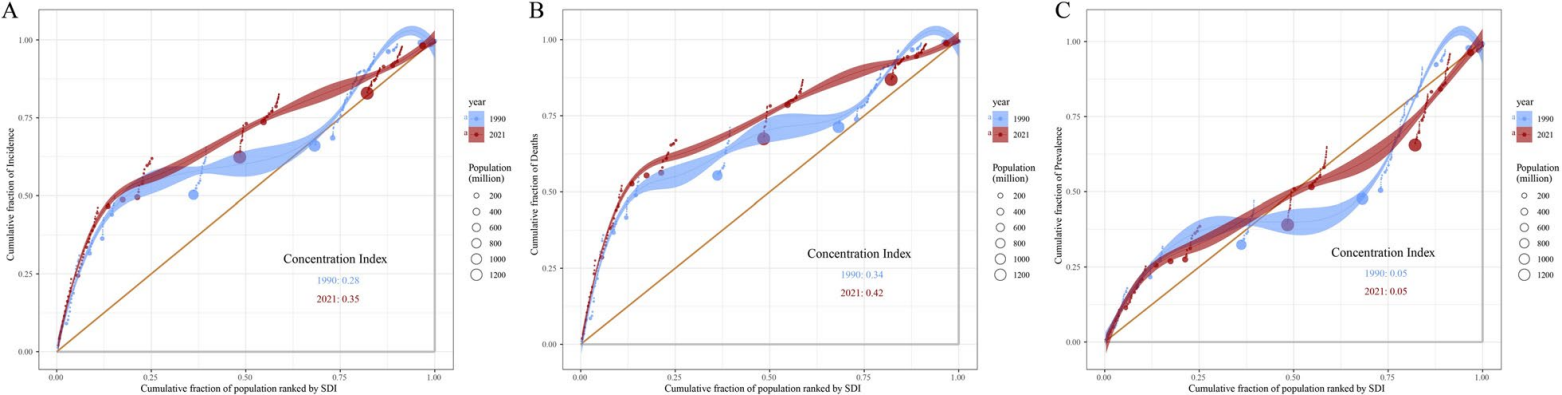
Concentration curves of CML anomaly incidence, mortality and prevalence by SDI in 1990 and 2021. **A** Concentration curve for CML anomaly incidence, with the concentration index for 1990 and 2021; **B** concentration curve for CML anomaly mortality, with the concentration index for 1990 and 2021; **C** concentration curve for CML anomaly prevalence, with the concentration index for 1990 and 2021. The orange diagonal line represents perfect equality, where CML anomalies incidence, mortality and prevalence would be equally distributed across all SDI levels. Shaded areas represent the 95% confidence intervals of CI. *CML* congenital musculoskeletal and limb, *SDI* socio-demographic index, *CI* concentration index

As demonstrated in Fig. 7A, the cumulative curves deviated from the line of equality (orange diagonal) in both 1990 and 2021, indicating that the prevalence of CML anomalies was more concentrated in areas with lower SDI values. The CI was 0.28 in 1990 and 0.35 in 2021, suggesting that inequality in the prevalence of CML anomalies increased with respect to socioeconomic status (Fig. 7A). A similar increase was observed in the CI for CML anomaly mortality, which rose from 0.34 in 1990 to 0.42 in 2021, suggesting that in 2021, individuals residing in areas with lower SDI values experienced higher mortality rates due to CML anomalies. This finding indicated an increase in mortality inequalities by socioeconomic status (Fig. 7B). Conversely, the cumulative curve for the prevalence of CML anomalies exhibited a closer proximity to the line of equality, with CIs of 0.05 in both 1990 and 2021, suggesting that the prevalence of CML anomalies was more equitably distributed across diverse SDI levels, and the degree of inequality remained relatively constant over time (Fig. 7C).

## Discussion

This study provides a comprehensive analysis of temporal trends in the incidence, mortality, and prevalence of CML anomalies at global, regional, and national levels. The analysis revealed fluctuations in the incidence of CML anomalies from 1990 to 2021, with an overall decline in absolute numbers. In contrast, the mortality rate exhibited a downward trend, while the prevalence rate showed an upward trend. Furthermore, the analysis indicated that the incidence and prevalence of CML anomalies were higher in females than in males, whereas the mortality rate was higher in males than in females, suggesting synergistic interactions between biological susceptibility and systemic healthcare inequities. From a biological perspective, sex-linked genetic mechanisms likely contribute to the female predominance in incidence and prevalence. For instance, FLNA is an X-linked gene, and the abnormal function caused by its mutation differs significantly between female and male embryos. Male embryos cannot compensate for the mutation effect due to hemizygosity (carrying only one mutated X chromosome), resulting in fatal developmental defects, while females may survive and manifest skeletal deformities due to random X chromosome inactivation [32]. Conversely, androgen receptor signaling has been implicated in exacerbating musculoskeletal defect severity in males. Experimental studies indicate that testosterone enhances osteoclast activity and suppresses osteoblast differentiation, potentially leading to more severe bone deformities and complications in males [33]. From a healthcare equity perspective, the disproportionate mortality burden among males in low SDI regions highlights structural disparities. In low-income countries, men often delay seeking care due to financial burden or social stigma, which results in delayed access to corrective surgery [34]. Studies have also shown that access to and quality of surgery are often limited by economic, geographical, and other factors. In this setting, males are 1.8 times more likely than females to die while waiting for care, which may be related to the higher health risks and lower access to medical resources that males face in these settings [35, 36]. Due to widespread gender role stereotypes, such as the belief that"males should be tough"or"females need to be protected", family needs for the care of female infants are often more acceptable to healthcare workers. However, this concept may lead medical staff to simplify the examination process for boys in practice, resulting in a relatively high rate of missed diagnoses of limb deformities in boys. In addition, girls are more likely to have access to imaging because they are considered more"vulnerable"in resource-limited settings. However, some abnormal signs in boys may be mistaken for"developmental differences". At the same time, under the"protective tendency"of cultural norms, girls will have priority access to surgery or rehabilitation resources [37]. These findings aligned with our data showing concentrated male mortality in regions with fragmented healthcare systems (Fig. 5C). The morbidity rates of CML anomalies in 1990 and 2021 were predominantly concentrated in lower SDI regions. In 1990 and 2021, the incidence of CML anomalies was predominantly concentrated in lower SDI regions, with a CI of 0.28 in 1990 and 0.35 in 2021. A similar distribution was observed in the mortality rate, which exhibited a CI of 0.34 in 1990 and 0.42 in 2021. This suggests that lower SDI regions experience inadequate prenatal healthcare coverage, insufficient capacity to save deliveries and newborns, and challenges in accessing healthcare resources and managing maternal health [27, 38–40]. These populations are susceptible to suboptimal intake of essential nutrients and exposure to environmental pollutants [41, 42]. In addition, lower SDI regions encounter challenges due to inadequate screening technology adoption and insufficient public awareness, compounded by inadequate policy support, which hinders efforts to regulate the progression of the disease [43]. The issue of growing health inequalities in morbidity and mortality between countries and regions at different levels of social development is of significant concern [44], and trends are more stable. The integration of Bayesian UIs and frequentist CIs offers a comprehensive approach to interpreting uncertainty in epidemiological data [14]. For example, the stable ASIR observed in high SDI countries, reflected by narrow UIs, contrasts sharply with the more fluctuating estimates seen in low SDI regions, where the UIs are broader. By integrating Bayesian UIs and frequentist CIs, this study provides multi-layered uncertainty interpretation. For instance, narrow UIs in high SDI countries (e.g., Japan’s ASPR UI: 394.40–605.90) reflect robust data quality, while wider UIs in low SDI regions (e.g., Afghanistan’s ASMR UI: 0.30–1.60) necessitate cautious interpretation of point estimates. EAPC CIs further distinguish true trends from noise: a mortality EAPC of −25.46% (CI: −30.12% to −20.80%) confirms a sustained decline. This dual-validation framework enhances result reliability, particularly in data-limited settings [3, 27]. This variation highlights the importance of context-specific interventions, tailored to the distinct challenges faced by different regions. Additionally, the use of CIs in the EAPC trend classification helps to temper the overinterpretation of point estimates, particularly in areas with sparse data, where uncertainty is inherently higher. By combining Bayesian and frequentist methods, this study provides a more nuanced understanding of epidemiological trends, ensuring more accurate and reliable interpretations, especially in regions with limited data quality [14]. However, the prevalence of the condition was more evenly distributed across areas with different SDI levels, with a CI of 0.05 for both years, suggesting that the extent of health inequalities in prevalence did not change significantly over time.

The integration of 95% UIs and CIs in this study further elucidates these disparities. Wider UIs for ASIR and ASMR in low SDI regions (e.g., sub-Saharan Africa) reflect systemic limitations in congenital anomaly surveillance, including underdiagnosis and fragmented data registries, whereas narrower intervals in high SDI settings underscore the reliability of estimates derived from robust diagnostic infrastructure [13]. Similarly, the classification of temporal trends through EAPC’s 95% CIs—whether increasing, decreasing, or stable—provides a nuanced understanding of intervention efficacy. For instance, stable trends (CI spanning zero) in certain regions may indicate stagnating preventive efforts despite apparent declines in point estimates, emphasizing the need for context-specific strategies [14]. These methodological insights align with GBD 2021’s emphasis on transparent uncertainty reporting, which is critical for prioritizing resource allocation and monitoring progress in global health agendas [13].

The ARIMA model predicted an increase in CML anomalies from 2,437,890.12 cases in 2021 to 2,449,310.78 cases in 2022, followed by a plateau from 2023 to 2031. Concurrently, the model indicated a decreasing trend in CML-related mortality and a continued increase in morbidity. This observed divergence could be attributed to several factors. Firstly, advancements in medical diagnostic technologies, including prenatal ultrasonography and standardized registration systems, have significantly enhanced the early detection and documentation of CML anomalies [45–47]. Secondly, heightened public awareness of CML, coupled with the effective dissemination of preventative measures, such as genetic testing (e.g., *COL6 A1-3*, *LAMA2* gene screening) and antenatal counseling, has contributed to the stabilization of CML prevalence following a transient period of growth [48–50]. In addition, improvements in public health awareness, widespread implementation of therapeutic interventions, reductions in socio-economic deprivation, and advancements in neonatal and pediatric intensive care have facilitated effective early-stage treatment of CML anomalies. This has resulted in a reduction in complication incidence and mortality risk, explaining the observed downward trend in CML-related deaths [51–53]. However, despite the decline in mortality, the persistent increase in morbidity can be partially explained by several factors. Prenatal exposure to environmental toxins, including pesticides, heavy metals (e.g., mercury), and industrial chemicals, is significantly associated with congenital malformations [54, 55]. Besides, maternal drug use and viral infections can disrupt fetal musculoskeletal development [56]. Inadequate maternal folic acid intake also contributes to the incidence of these anomalies [57]. The aforementioned reduction in mortality, paradoxically, contributes to the accumulation of cases, thereby increasing the overall morbidity.

The transient increase in the global incidence of CML anomalies was primarily driven by population growth, with population aging contributing to a lesser extent. Conversely, epidemiological shifts exerted a negative influence on this trend. The global decline in CML anomalies-related mortality was predominantly attributable to epidemiological changes and population aging, with the most significant reductions observed in middle SDI quintile regions. However, population growth counteracted this decline, contributing to an increase in the absolute number of deaths. Finally, the sustained increase in CML morbidity was primarily influenced by population growth, with a secondary contribution from epidemiological changes, a phenomenon particularly evident in low-middle SDI regions. Population growth directly expands the fertility base, leading to an increase in the absolute number of CML anomaly cases, thus contributing to the transient increase in morbidity and the persistent increase in mortality and overall illness burden. In addition, population expansion may be associated with increased genetic diversity and a higher cumulative risk of low-frequency deleterious mutations [58]. The incidence and prevalence of musculoskeletal malformations are reportedly significantly elevated in developing countries compared to developed countries, largely due to higher fertility rates [59, 60]. Advanced parental age, particularly maternal age, is associated with an increased rate of germ cell mutations and an elevated risk of chromosomal aneuploidy. A Norwegian cohort study demonstrated a 0.43 percentage point increase in the risk of foot malformations when parents were older than 45 years [61]. Advanced maternal age is also linked to metabolic disorders, such as gestational diabetes, which may exacerbate the risk of CML anomalies through mechanisms including chronic hyperglycemia-induced oxidative stress and impaired fetal osteogenesis [62, 63]. A meta-analysis of 12 cohort studies further confirmed that maternal diabetes increases the odds of congenital limb defects by 1.50-fold (95% CI: 1.23–1.92) [64].

Advances in medical technology have prolonged the survival of patients with CML anomalies. Consequently, as these individuals reach geriatric age, the primary cause of mortality has shifted from congenital anomalies to age-related comorbidities. Multi-system complications in elderly CML patients are managed through surgical interventions and rehabilitation therapies, thereby reducing the risk of direct mortality. While aging is associated with decreased bone mineral density, osteoporosis, gait abnormalities, and increased fall risk, these symptoms can be mitigated through pharmacological interventions and physiotherapy, further contributing to the observed decline in CML-related mortality [65–67]. For a more scientific tone, consider changing to “The mechanisms by which epidemiological shifts influence morbidity, mortality, and prevalence have been previously elucidated. Furthermore, this study observed an inverse correlation between the baseline ASIR and the EAPC, with the EAPC decreasing when the baseline ASIR exceeded 40. A plausible explanation for this phenomenon is that CML anomalies may not elicit significant public health attention when the baseline ASIR is low. However, as the disease burden increases and reaches a critical threshold, it attracts increased public attention and prompts targeted control measures. In conclusion, we recommend continued emphasis on early screening and intervention for CML anomalies in high-risk populations, including those with prenatal exposure to chemical substances and heavy metals, as well as those born to older mothers. Furthermore, the promotion of evidence-based treatment strategies is crucial to minimize the disease burden associated with CML anomalies.

The etiology of CML anomalies remains incompletely elucidated, exhibiting a complex interplay of genetic, environmental, and maternal health factors. CML anomalies frequently arise from mutations in specific genes or chromosomal abnormalities. For example, *RYR1* mutations are implicated in central core disease and fetal ankylosing spondylitis [68], gene dosage effects in specific regions of partial chromosomal trisomies affect foot development [69], and Mendelian inheritance patterns are observed in CML abnormalities, with approximately 12% of congenital spinal deformities exhibiting a monogenic etiology, and polydactyly often following an autosomal dominant inheritance pattern [70]. Furthermore, prenatal exposure to opioids and anti-epileptic drugs significantly increases the risk of limb defects, particularly during the second and third trimesters [71]. Similarly, prenatal exposure to pesticides and heavy metals is significantly associated with congenital limb deformities [72, 73]. Exposure to particulate matter with an aerodynamic diameter of 2.5 µm or less (PM_2.5_) has been identified as a potential causative factor for musculoskeletal deformities [42]. Nicotine and alcohol consumption during pregnancy are also associated with increased risk [74, 75], and an observed increase in CML anomalies occurred during viral epidemics [56]. Finally, maternal health status plays a crucial role in CML anomaly etiology. It has been suggested that gestational diabetes and hyperlipidaemia may affect fetal bone development through oxidative stress mechanisms [63], and maternal hypertension is associated with an elevated risk of fetal limb defects [64]. Insufficient maternal folic acid intake has been linked to neural tube defects; a Tanzanian study demonstrated a doubled risk of congenital anomalies in areas with low folic acid supplementation rates [57]. Overall, the etiology of CML anomalies is complex and requires a comprehensive diagnostic approach that integrates genetic testing, assessment of environmental exposures, and maternal health management. Perinatal nutritional interventions and the avoidance of teratogenic exposures are important preventive strategies in clinical practice.

Notably, our temporal analysis, spanning 1990–2021, encompassed the COVID-19 pandemic (2019–2021), a period that introduced unprecedented challenges to global CML anomaly surveillance systems. The redirection of resources within overburdened healthcare infrastructures, particularly in low- and middle-income countries, compromised critical services, including antenatal ultrasonography and time-sensitive orthopedic interventions (e.g., digit duplication corrections). This disruption potentially skewed incidence documentation and exacerbated disability-related sequelae [76, 77]. Although epidemiological investigations have established correlations between gestational SARS-CoV-2 exposure and adverse perinatal outcomes (e.g., prematurity), contemporary meta-analyses have refuted causal relationships with congenital musculoskeletal malformations [78, 79]. These pandemic-induced disruptions underscore the necessity of assessing crisis-driven healthcare fragmentation mechanisms that may perpetuate disparities in congenital disorder management.

This investigation provides a systematic characterization of the global epidemiological landscape of CML anomalies across three decades. However, several inherent limitations require explicit acknowledgement. The analytical approach, while leveraging standardized GBD metrics, is constrained by its reliance on aggregated datasets, which may inadequately represent disease burden in resource-limited settings. These limitations are particularly pronounced in low SDI regions, where diagnostic capacity gaps and fragmented vital registration systems compromise data completeness. Furthermore, the current paradigm’s inability to incorporate patient-specific clinical and molecular profiles precludes mechanistic exploration of critical biological interactions, notably the synergistic effects between gestational toxicant exposure and hereditary predisposition, which are essential for precision medicine applications. The existing nosological framework’s insufficient granularity in anomaly subtyping (e.g., the failure to distinguish syndromic from isolated limb defects) further obscures differential etiological pathways and therapeutic response patterns. Of particular concern is the predictive modeling architecture’s exclusion of contemporary environmental covariates, including climate-mediated toxicant dispersion patterns and novel industrial compound exposures. Besides, methodological constraints exist; while the ARIMA model effectively captures long-term trends and autocorrelations in time series data, its sensitivity to seasonal fluctuations and sudden events is relatively limited, thereby restricting its utility for sustainable health system planning. Advancing this field necessitates a dual focus on methodological innovation and implementation science.

The significant burden of CML anomalies in low-SDI countries urgently demands comprehensive, action-oriented strategies to systematically address healthcare disparities and environmental risk factors. Our findings on the concentration of mortality in resource-limited settings (Fig. 6C) and the role of population growth in driving case burdens (Fig. 3A) underscore the need for decentralized prenatal screening networks. Such systems could integrate task-sharing models and streamlined diagnostic technologies at the community level, prioritizing capacity-building programs to train frontline healthcare workers in identifying critical gestational windows for congenital anomalies. For instance, Ghana’s implementation of standardized screening protocols at 13 weeks and 14–23 weeks of pregnancy, coupled with nationwide health worker training, directly increased anomaly detection rates by 62% over two years, demonstrating the scalability of community-driven screening systems [77].

Governments worldwide should enforce mandatory food fortification policies and develop stringent supply chain oversight mechanisms to ensure universal access to micronutrient supplements, particularly in addressing deficiencies associated with congenital malformations. Mali’s success in achieving 80–90% coverage of folic acid-fortified wheat and corn flour across its population serves as a model, not only reducing neural tube defects but also creating a protective nutritional environment against CML anomalies through improved maternal folate status [80].

Healthcare systems must adopt standardized diagnostic tools and implement tiered referral pathways to accelerate intervention timelines for congenital abnormalities. By integrating the Ponseti method into primary care networks and training multidisciplinary teams, Haiti achieved corrective outcomes for 80% of congenital foot deformities within three months, while reducing relapse rates from 35 to 12% through structured follow-up protocols [81]. To ensure sustainable postoperative recovery, policymakers must institutionalize free surgical care policies alongside community rehabilitation networks. Thirteen African nations demonstrated this approach’s effectiveness by implementing free surgical care policies that boosted functional recovery rates by 40%, while Somaliland’s creation of localized rehabilitation networks reduced postoperative mortality from 12 to 5% through continuous care coordination [82].

Stringent environmental regulations paired with agricultural reforms are critical to mitigate prenatal exposure to teratogenic substances. The Bangladesh-Nigeria joint initiative on pesticide risk classification reduced CML anomalies-related Disability-Adjusted Life Years (DALYs) by 5–8% through phased bans on high-risk chemicals, while Pakistan’s prohibition of specific agrochemicals and promotion of alternative crops decreased annual limb defect incidence by 1.2%, showcasing the dual impact of regulatory action and sustainable farming practices [83, 84]. Global partnerships to adapt international standards to local realities are equally vital. Beyond national efforts, international collaborations have proven critical, as evidenced by Nepal’s 50% improvement in birth defect identification through UNICEF-supported training modules and telemedicine integration [85].

By synergizing scalable screening, nutritional security, accelerated care delivery, rehabilitation infrastructure, environmental governance, and cross-border capacity-building, these proactive measures collectively target the multifactorial drivers of CML anomalies. Aligned with SDG health equity objectives, this integrated framework empowers low-SDI countries to systematically reduce preventable disability burdens, transform fragmented services into coordinated health systems, and ultimately break the cycle of disparities perpetuating CML anomalies through evidence-based, population-wide interventions anchored in sustainability and equity.

Optimizing data capture in marginalized regions could be achieved through decentralized digital surveillance networks, synergized with blockchain-based validation mechanisms to ensure data integrity. Establishing federated learning systems that interface GBD repositories with regional birth defect registries would facilitate multi-scale analyses of exposure-disease relationships, particularly in populations with occupational or environmental toxicant burdens. The development of consensus-driven phenotyping standards through international collaborations represents an urgent priority to enable comparative effectiveness research across anomaly subtypes. From a methodological perspective, incorporating temporal environmental exposure gradients and longitudinal biospecimen-derived biomarkers into prognostic algorithms could substantially enhance predictive validity in dynamic risk environments. Policy formulation should be informed by comparative analyses of intervention scalability, particularly evaluating nutritional fortification protocols and surgical care delivery models optimized for resource-variant contexts. International health governance bodies must prioritize capacity-building initiatives to standardize diagnostic protocols and expand access to multidisciplinary rehabilitation services. Through such multidimensional advancements, subsequent research endeavors stand to transform epidemiological insights into precision prevention strategies, ultimately attenuating the multidimensional health inequities perpetuated by these congenital disorders.

## Conclusion

This study estimated temporal trends in morbidity and mortality associated with CML anomalies at global, national, and regional levels from 1990 to 2021. The analysis revealed unfavorable trends in countries with lower SDIs, indicating a need for targeted and context-specific strategies to address the burden of CML anomalies in these regions.

## Supplementary Information

Additional file 1.

## Data Availability

The Global Health Data Exchange (GHDx) query tool (http://ghdx.healthdata.org/gbd-results-tool) was utilised to collate data on the number of cases of congenital musculoskeletal and limb anomalies from 1990 to 2021.
