# Supplementary material for: Global, regional, and national burden of congenital musculoskeletal and limb anomalies, 1990–2021: a systematic analysis of the global burden of disease in 2021
Source: Trop Med Health. 2025 May 12;53:68. doi: 10.1186/s41182-025-00750-4 (PMC12067968; doi:10.1186/s41182-025-00750-4)
Supplement: Supplementary file 1 — Additional file 1. [file 41182_2025_750_MOESM1_ESM.docx]

Table S1. The congenital musculoskeletal and limb anomalies cases and age standardized incidence from 1990 to 2021 in 204 countries and territories.

| Cause | Location | 1990 | | 2021 | | 1990-2021 |
| --- | --- | --- | --- | --- | --- | --- |
|  |  | Incident cases  No. ×10^3^ (95% UI) | ASIR per 100,000  No. (95% UI) | Incident cases  No. ×10^3^ (95% UI) | ASIR per 100,000  No. (95% UI) | EAPC  No. (95% CI) |
| Congenital musculoskeletal and limb anomalies | Afghanistan | 13246.8 (9451.7-18438.3) | 62.6 (44.7-87.1) | 30034.6 (21020.8-42568.2) | 51.7 (36.2-73.3) | -0.36 (-0.45--0.27) |
| Congenital musculoskeletal and limb anomalies | American Samoa | 19.1 (13.3-28.1) | 22.7 (15.8-33.4) | 9.6 (6.4-14.2) | 29.7 (19.9-44) | 0.96 (0.8-1.12) |
| Congenital musculoskeletal and limb anomalies | Angola | 11175.7 (7544.4-16434.6) | 46.4 (31.3-68.2) | 25786.9 (17296.2-37672.4) | 44.5 (29.8-65) | -0.21 (-0.24--0.18) |
| Congenital musculoskeletal and limb anomalies | Antigua and Barbuda | 13.5 (9.3-19.7) | 23 (15.9-33.6) | 12.9 (8.9-18.8) | 25.7 (17.7-37.6) | 0.19 (0.12-0.25) |
| Congenital musculoskeletal and limb anomalies | Arab Republic of Egypt | 41251.6 (28480.6-58723) | 45.7 (31.5-65) | 53601.5 (36994.4-78637.4) | 43.3 (29.9-63.6) | -0.05 (-0.14-0.03) |
| Congenital musculoskeletal and limb anomalies | Argentine Republic | 23195.6 (16102.1-32045.4) | 69.5 (48.3-96) | 18869.9 (13305.4-26485.6) | 73.3 (51.7-102.8) | 0.16 (0.08-0.24) |
| Congenital musculoskeletal and limb anomalies | Australia | 5778.3 (3972.8-8027.6) | 46.8 (32.1-65) | 7055.6 (4895.7-9943.1) | 49.5 (34.4-69.8) | 0.16 (0.12-0.2) |
| Congenital musculoskeletal and limb anomalies | Bangladesh | 66585.4 (45156.9-95329.1) | 32.7 (22.2-46.8) | 38827.8 (26845.8-56567.1) | 28.9 (20-42.1) | -0.37 (-0.46--0.28) |
| Congenital musculoskeletal and limb anomalies | Barbados | 44 (29.9-64.3) | 22 (14.9-32.1) | 30.8 (21.2-45.1) | 24.4 (16.8-35.7) | 0.16 (0.06-0.25) |
| Congenital musculoskeletal and limb anomalies | Belize | 94.8 (64.8-138.7) | 31.3 (21.4-45.9) | 129.6 (89-194.1) | 35.2 (24.2-52.7) | 0.04 (-0.14-0.22) |
| Congenital musculoskeletal and limb anomalies | Benin | 4601.9 (3097.3-6533.6) | 40.3 (27.1-57.3) | 10394.5 (6886.1-15144.7) | 41.4 (27.4-60.4) | 0.2 (0.14-0.25) |
| Congenital musculoskeletal and limb anomalies | Bermuda | 7.6 (5.3-11.4) | 17.9 (12.5-26.7) | 4.7 (3.3-6.9) | 20 (13.9-29.4) | 0.24 (0.18-0.29) |
| Congenital musculoskeletal and limb anomalies | Bhutan | 360.3 (242.3-510.2) | 33.8 (22.7-47.8) | 189.2 (126.7-274) | 31.6 (21.2-45.8) | -0.29 (-0.35--0.23) |
| Congenital musculoskeletal and limb anomalies | Bolivarian Republic of Venezuela | 12106.6 (7997.3-17843.6) | 45.9 (30.3-67.6) | 8850.5 (5909.1-12842.8) | 40.8 (27.2-59.2) | -0.21 (-0.35--0.07) |
| Congenital musculoskeletal and limb anomalies | Bosnia and Herzegovina | 904.7 (627.9-1309.2) | 27.8 (19.3-40.3) | 363.8 (252.9-506) | 27.2 (18.9-37.9) | -0.61 (-0.81--0.41) |
| Congenital musculoskeletal and limb anomalies | Botswana | 718.4 (475.9-1058.8) | 32.3 (21.4-47.6) | 756.3 (503.5-1128.4) | 32.5 (21.6-48.5) | 0.07 (0.01-0.13) |
| Congenital musculoskeletal and limb anomalies | Brunei Darussalam | 248.9 (170-361) | 73.7 (50.3-106.9) | 226.4 (157.3-323) | 76.7 (53.3-109.4) | 0.08 (0.04-0.12) |
| Congenital musculoskeletal and limb anomalies | Burkina Faso | 9639.5 (6322.2-14027.5) | 43.4 (28.5-63.1) | 21725.9 (14384.3-30382.6) | 47.7 (31.6-66.7) | 0.3 (0.22-0.39) |
| Congenital musculoskeletal and limb anomalies | Burundi | 5880.8 (4035.4-8428.7) | 47.1 (32.3-67.5) | 11185.3 (7732.1-16266.9) | 49.9 (34.5-72.6) | 0.13 (0.05-0.21) |
| Congenital musculoskeletal and limb anomalies | Cabo Verde | 121.5 (81.3-185.6) | 20.2 (13.5-30.8) | 76.5 (51.1-119.8) | 18.8 (12.6-29.4) | -0.34 (-0.44--0.24) |
| Congenital musculoskeletal and limb anomalies | Cameroon | 8807.6 (5781.8-12949.3) | 38.5 (25.2-56.5) | 19983.2 (13046.3-28878.8) | 40.2 (26.2-58.1) | 0.18 (0.1-0.26) |
| Congenital musculoskeletal and limb anomalies | Canada | 5883 (4122.6-8419.7) | 30.2 (21.1-43.2) | 4526.1 (3109.5-6577.6) | 25.5 (17.5-37.1) | -0.33 (-0.38--0.27) |
| Congenital musculoskeletal and limb anomalies | Central African Republic | 3120.9 (2129.9-4463.5) | 51.5 (35.2-73.7) | 5292.5 (3702.7-7532.8) | 58.2 (40.7-82.8) | 0.5 (0.46-0.54) |
| Congenital musculoskeletal and limb anomalies | Chad | 7166.1 (4826.6-10243.8) | 46.7 (31.4-66.7) | 21296.8 (14577.5-31539.9) | 51.4 (35.2-76.2) | 0.17 (0.1-0.23) |
| Congenital musculoskeletal and limb anomalies | Commonwealth of Dominica | 26.6 (18.1-38.3) | 29.7 (20.2-42.8) | 11.1 (7.4-16.4) | 35.8 (24-52.9) | 0.51 (0.46-0.56) |
| Congenital musculoskeletal and limb anomalies | Commonwealth of the Bahamas | 71.4 (49.4-104.6) | 26.9 (18.6-39.4) | 56.1 (39.2-81.7) | 29.2 (20.4-42.6) | 0.17 (0.11-0.23) |
| Congenital musculoskeletal and limb anomalies | Comoros | 342.3 (231.2-491.5) | 36.2 (24.4-51.9) | 302 (205-427.2) | 37 (25.1-52.4) | 0.06 (-0.02-0.14) |
| Congenital musculoskeletal and limb anomalies | Congo | 1724.5 (1147.5-2450.5) | 39.4 (26.2-56) | 2341.2 (1550.5-3466.4) | 38.3 (25.4-56.8) | -0.23 (-0.34--0.12) |
| Congenital musculoskeletal and limb anomalies | Cook Islands | 3.8 (2.6-5.6) | 17.9 (12.1-26.6) | 1.9 (1.3-3) | 18.2 (12.1-28.8) | 0.22 (0.09-0.36) |
| Congenital musculoskeletal and limb anomalies | C么te d'Ivoire | 10181.2 (6800.6-14847.6) | 38.3 (25.6-55.9) | 17787.2 (11874.5-25871.2) | 39.2 (26.2-57) | 0.01 (-0.07-0.09) |
| Congenital musculoskeletal and limb anomalies | Czech Republic | 1649 (1145.1-2328.9) | 26.6 (18.5-37.6) | 1357.6 (945.9-1941.3) | 26.5 (18.5-38) | -0.32 (-0.65-0) |
| Congenital musculoskeletal and limb anomalies | Democratic People's Republic of Korea | 7926 (5216.2-11948.4) | 30.7 (20.2-46.3) | 5676.6 (3725.4-8275.2) | 40.2 (26.4-58.6) | 0.98 (0.78-1.18) |
| Congenital musculoskeletal and limb anomalies | Democratic Republic of the Congo | 37497.7 (25004.2-53415.6) | 43.6 (29.1-62.1) | 58941.6 (39229.1-85456.9) | 43.1 (28.7-62.5) | 0.17 (0.09-0.26) |
| Congenital musculoskeletal and limb anomalies | Democratic Republic of Timor-Leste | 893 (619.7-1268.1) | 53.5 (37.2-76) | 921.9 (634.4-1297.8) | 47.1 (32.4-66.3) | -0.52 (-0.61--0.43) |
| Congenital musculoskeletal and limb anomalies | Democratic Socialist Republic of Sri Lanka | 4563.2 (3066.1-6692.3) | 26.4 (17.8-38.8) | 3060.1 (2075-4652.5) | 21.1 (14.3-32.1) | -0.76 (-0.88--0.64) |
| Congenital musculoskeletal and limb anomalies | Djibouti | 239.5 (162.1-350.9) | 32 (21.7-46.9) | 448.8 (293.9-659) | 31 (20.3-45.5) | -0.26 (-0.38--0.14) |
| Congenital musculoskeletal and limb anomalies | Dominican Republic | 4230.7 (2921-6171) | 39.3 (27.1-57.3) | 3989.7 (2798.1-5781.6) | 38.9 (27.3-56.4) | 0.07 (-0.04-0.18) |
| Congenital musculoskeletal and limb anomalies | Eastern Republic of Uruguay | 1659.2 (1167.6-2296) | 61.8 (43.5-85.5) | 1144.8 (805.4-1582.3) | 67 (47.1-92.6) | 0.25 (0.21-0.3) |
| Congenital musculoskeletal and limb anomalies | Equatorial Guinea | 426.5 (285-605.1) | 43.3 (28.9-61.4) | 588 (386.7-867.4) | 32.7 (21.5-48.3) | -1.22 (-1.37--1.08) |
| Congenital musculoskeletal and limb anomalies | Eritrea | 3210.3 (2136-4638.9) | 45.2 (30.1-65.3) | 4474.8 (3018.8-6449.8) | 47.8 (32.3-68.9) | 0.03 (-0.05-0.1) |
| Congenital musculoskeletal and limb anomalies | Eswatini | 504.4 (342.7-709.3) | 32.6 (22.1-45.8) | 514.6 (352.8-719.4) | 37.1 (25.4-51.9) | 0.31 (0.18-0.43) |
| Congenital musculoskeletal and limb anomalies | Ethiopia | 53224.4 (36507.5-75058.5) | 45.6 (31.3-64.3) | 66301.4 (45769.3-93730.1) | 39.7 (27.4-56.1) | -0.52 (-0.57--0.47) |
| Congenital musculoskeletal and limb anomalies | Federal Republic of Germany | 14709 (12160.5-17619.3) | 34.7 (28.7-41.6) | 12286.1 (8327.6-17852.3) | 32.5 (22-47.2) | -0.27 (-0.55-0.01) |
| Congenital musculoskeletal and limb anomalies | Federated States of Micronesia | 54.3 (36.8-80.5) | 35.9 (24.3-53.2) | 31.6 (21.7-44.6) | 35.4 (24.3-49.9) | -0.11 (-0.16--0.05) |
| Congenital musculoskeletal and limb anomalies | Federative Republic of Brazil | 54522.1 (42184.6-71066.5) | 35.2 (27.2-45.9) | 51161.5 (36455.5-72061.3) | 32.2 (22.9-45.3) | -0.34 (-0.45--0.23) |
| Congenital musculoskeletal and limb anomalies | French Republic | 8871 (8306.8-9452.9) | 24.1 (22.5-25.7) | 9114.5 (7142.4-11290) | 27.5 (21.5-34) | 0.49 (0.3-0.68) |
| Congenital musculoskeletal and limb anomalies | Gabon | 541.7 (357.1-816.3) | 31.7 (20.9-47.8) | 617.6 (407.8-919) | 29.9 (19.8-44.5) | -0.28 (-0.33--0.23) |
| Congenital musculoskeletal and limb anomalies | Gambia | 754.1 (501-1125) | 34.8 (23.1-51.9) | 1318.3 (852.6-2015) | 35.6 (23-54.4) | 0.1 (0.03-0.17) |
| Congenital musculoskeletal and limb anomalies | Georgia | 1317.1 (899.8-1913.3) | 31.8 (21.7-46.1) | 842 (702.1-986.4) | 39.2 (32.7-45.9) | 1.28 (0.98-1.58) |
| Congenital musculoskeletal and limb anomalies | Ghana | 10361.7 (6941.1-15213.2) | 35.8 (24-52.5) | 17043.6 (10916.5-25245.7) | 36.7 (23.5-54.3) | 0.09 (0.04-0.14) |
| Congenital musculoskeletal and limb anomalies | Global | 2521672.1 (1793995.9-3516239.1) | 39.4 (28-54.9) | 2437890.1 (1737729.8-3355568.4) | 39.4 (28.1-54.2) | 0.01 (-0.01-0.02) |
| Congenital musculoskeletal and limb anomalies | Grand Duchy of Luxembourg | 114.5 (81.1-159.6) | 47.3 (33.5-65.9) | 135 (93.3-191.6) | 41.5 (28.7-58.9) | -0.18 (-0.29--0.08) |
| Congenital musculoskeletal and limb anomalies | Greenland | 26.8 (18.5-37.6) | 46.3 (32-65) | 17.2 (12.2-23.2) | 45.9 (32.6-62.2) | 0.23 (0.1-0.35) |
| Congenital musculoskeletal and limb anomalies | Grenada | 36.6 (25.3-53.3) | 31.6 (21.8-46) | 20.7 (14.3-31.1) | 32 (22.1-47.9) | 0.24 (0.17-0.32) |
| Congenital musculoskeletal and limb anomalies | Guam | 32.4 (22.2-48.4) | 17.8 (12.2-26.6) | 24.2 (15.6-38.2) | 19.1 (12.3-30.1) | 0.33 (0.25-0.4) |
| Congenital musculoskeletal and limb anomalies | Guinea | 6518.6 (4507.6-9584.2) | 46.7 (32.3-68.7) | 11765.5 (7921.7-17000.6) | 49.4 (33.2-71.3) | 0.28 (0.18-0.38) |
| Congenital musculoskeletal and limb anomalies | Guinea-Bissau | 1053 (688-1511.2) | 47.8 (31.2-68.6) | 1793.6 (1230.6-2656.8) | 52.2 (35.8-77.3) | 0.28 (0.24-0.31) |
| Congenital musculoskeletal and limb anomalies | Hashemite Kingdom of Jordan | 2025.6 (1389.2-2987.9) | 31.7 (21.7-46.8) | 2926.5 (2013.9-4424.9) | 28.3 (19.4-42.7) | -0.74 (-0.89--0.59) |
| Congenital musculoskeletal and limb anomalies | Hellenic Republic | 2640.9 (1889.4-3682.7) | 52.5 (37.5-73.1) | 2563 (1897.8-3384.7) | 64.5 (47.7-85.1) | 0.91 (0.79-1.02) |
| Congenital musculoskeletal and limb anomalies | Hungary | 1172.8 (802.8-1695) | 19.4 (13.3-28) | 847.9 (576.4-1250.1) | 19.9 (13.5-29.3) | -0.21 (-0.3--0.11) |
| Congenital musculoskeletal and limb anomalies | Independent State of Papua New Guinea | 2838.7 (1979.6-4105.5) | 40.1 (28-58) | 7632.7 (5211.3-10366.5) | 46.6 (31.8-63.3) | 0.55 (0.49-0.62) |
| Congenital musculoskeletal and limb anomalies | Independent State of Samoa | 74.2 (49.9-108.6) | 28.7 (19.3-42) | 87 (58.2-129.5) | 29.3 (19.6-43.6) | 0.23 (0.16-0.3) |
| Congenital musculoskeletal and limb anomalies | India | 363759.9 (257820.3-504394.2) | 30.2 (21.4-41.9) | 339702.3 (239365.5-470274.4) | 32.4 (22.8-44.8) | 0.11 (0.06-0.17) |
| Congenital musculoskeletal and limb anomalies | Ireland | 767.1 (692.3-852.4) | 29.3 (26.4-32.5) | 1008.5 (726.1-1222.9) | 37.2 (26.8-45.1) | 1.61 (1.31-1.9) |
| Congenital musculoskeletal and limb anomalies | Islamic Republic of Iran | 27977.5 (19309.5-39783.9) | 37.6 (26-53.5) | 18235.1 (12735.7-26012.7) | 36.8 (25.7-52.5) | -0.21 (-0.41--0.01) |
| Congenital musculoskeletal and limb anomalies | Jamaica | 626 (425-924.3) | 22.6 (15.4-33.4) | 431.4 (303-607.5) | 27.2 (19.1-38.3) | 0.3 (0.17-0.42) |
| Congenital musculoskeletal and limb anomalies | Japan | 37306.5 (26553.2-51642.6) | 62.3 (44.3-86.2) | 24262.8 (17834.4-33136.4) | 58.7 (43.1-80.1) | -0.37 (-0.44--0.3) |
| Congenital musculoskeletal and limb anomalies | Kenya | 14092.5 (9880.2-20224.3) | 29.9 (20.9-42.8) | 18918.7 (13216.3-26787.5) | 33.4 (23.3-47.3) | 0.49 (0.44-0.54) |
| Congenital musculoskeletal and limb anomalies | Kingdom of Bahrain | 191.5 (129.2-285.6) | 30 (20.2-44.7) | 206.3 (143-304) | 24.4 (16.9-35.9) | -0.78 (-0.86--0.71) |
| Congenital musculoskeletal and limb anomalies | Kingdom of Belgium | 1348.8 (1140.3-1720.4) | 22.4 (18.9-28.5) | 1597.9 (1195.5-2032.5) | 29 (21.7-36.9) | 1.18 (1.05-1.3) |
| Congenital musculoskeletal and limb anomalies | Kingdom of Cambodia | 10584.5 (7290.4-14933) | 51.5 (35.5-72.6) | 7892.8 (5378.7-11312.4) | 46.1 (31.4-66.1) | -0.54 (-0.62--0.46) |
| Congenital musculoskeletal and limb anomalies | Kingdom of Denmark | 678.3 (567.3-817.3) | 22.1 (18.5-26.7) | 774.1 (534.8-1100) | 25.2 (17.4-35.9) | 0.32 (0.22-0.42) |
| Congenital musculoskeletal and limb anomalies | Kingdom of Norway | 1002.3 (714.9-1398.9) | 35 (25-48.9) | 948.6 (683.5-1345.6) | 35.4 (25.5-50.3) | -0.24 (-0.45--0.02) |
| Congenital musculoskeletal and limb anomalies | Kingdom of Spain | 4352.8 (3943.9-4909.1) | 22.7 (20.6-25.6) | 6869 (4922.4-9401.2) | 42.6 (30.5-58.3) | 2.77 (2.32-3.21) |
| Congenital musculoskeletal and limb anomalies | Kingdom of Sweden | 2634.2 (1863-3671.6) | 44.1 (31.2-61.5) | 2670.3 (1911.7-3732) | 48.7 (34.8-68) | 0.28 (0.2-0.36) |
| Congenital musculoskeletal and limb anomalies | Kingdom of Thailand | 12199 (8203.6-17909.7) | 24.5 (16.5-35.9) | 6053 (4144.8-8903.1) | 23.5 (16.1-34.5) | 0.11 (-0.03-0.25) |
| Congenital musculoskeletal and limb anomalies | Kingdom of the Netherlands | 2480.1 (2235-2792) | 26.1 (23.5-29.4) | 2273.9 (1617.1-3194.6) | 26.6 (18.9-37.3) | 0.41 (0.09-0.72) |
| Congenital musculoskeletal and limb anomalies | Kingdom of Tonga | 39.5 (26.5-58.5) | 25.3 (17-37.5) | 38.7 (25.9-58) | 27.1 (18.1-40.6) | 0.3 (0.26-0.34) |
| Congenital musculoskeletal and limb anomalies | Kyrgyz Republic | 2796.6 (1918.6-3970.4) | 44 (30.2-62.5) | 3207.3 (2171.3-4688.3) | 42.7 (28.9-62.4) | 0.01 (-0.08-0.11) |
| Congenital musculoskeletal and limb anomalies | Lao People's Democratic Republic | 4744.7 (3333-6702.9) | 56.9 (40-80.4) | 4307.6 (2990.6-6123.2) | 51.1 (35.5-72.7) | -0.5 (-0.55--0.45) |
| Congenital musculoskeletal and limb anomalies | Lebanon | 1245.1 (847.6-1870.1) | 30.4 (20.7-45.7) | 989.4 (664.5-1458.3) | 26.1 (17.5-38.4) | -0.79 (-0.91--0.67) |
| Congenital musculoskeletal and limb anomalies | Lesotho | 895.7 (627.9-1256.6) | 34.9 (24.5-49) | 850.4 (580-1191.7) | 41.9 (28.6-58.8) | 0.59 (0.53-0.65) |
| Congenital musculoskeletal and limb anomalies | Liberia | 2390.8 (1572.9-3440.4) | 44.3 (29.1-63.7) | 3229.4 (2147.9-4657.5) | 41.4 (27.5-59.7) | -0.21 (-0.34--0.08) |
| Congenital musculoskeletal and limb anomalies | Libya | 2198.9 (1476.2-3183.4) | 35.2 (23.6-50.9) | 1388.7 (967.1-2014.6) | 36.1 (25.1-52.3) | -0.04 (-0.15-0.08) |
| Congenital musculoskeletal and limb anomalies | Madagascar | 10411.5 (7200.3-15375.2) | 41.6 (28.8-61.5) | 18564.5 (12329.4-26052.5) | 45.8 (30.4-64.3) | 0.32 (0.2-0.44) |
| Congenital musculoskeletal and limb anomalies | Malawi | 11130.9 (7434.1-15879.8) | 46.4 (31-66.2) | 12845.7 (8670.2-18623.8) | 47.1 (31.8-68.3) | -0.13 (-0.2--0.06) |
| Congenital musculoskeletal and limb anomalies | Malaysia | 6229.6 (4205.7-9345.1) | 26.1 (17.6-39.1) | 5071 (3401.8-7372.6) | 22.1 (14.8-32.1) | -0.71 (-0.84--0.59) |
| Congenital musculoskeletal and limb anomalies | Mali | 10028.7 (7029.9-14261.7) | 46.8 (32.8-66.6) | 25016.4 (16636-36507) | 49 (32.6-71.5) | 0.07 (-0.03-0.17) |
| Congenital musculoskeletal and limb anomalies | Mauritania | 1503.3 (1005.2-2175.2) | 36.2 (24.2-52.4) | 1989.7 (1317-2984.9) | 30.7 (20.3-46) | -0.52 (-0.59--0.44) |
| Congenital musculoskeletal and limb anomalies | Mauritius | 237.6 (160-357.2) | 21.4 (14.4-32.2) | 126.7 (85-194.1) | 20.8 (13.9-31.8) | 0.1 (-0.01-0.21) |
| Congenital musculoskeletal and limb anomalies | Monaco | 4.5 (3.1-6.3) | 34.5 (23.7-48.6) | 4.6 (3.2-6.6) | 30.1 (20.9-42.7) | -0.49 (-0.55--0.43) |
| Congenital musculoskeletal and limb anomalies | Mongolia | 2048 (1428.4-2944.1) | 59.1 (41.2-85) | 2000.6 (1369.3-2816.7) | 54.6 (37.3-76.8) | -0.54 (-0.65--0.43) |
| Congenital musculoskeletal and limb anomalies | Montenegro | 92.9 (63-136.5) | 19.4 (13.2-28.5) | 85.4 (61.4-123.2) | 25.3 (18.2-36.4) | 0.52 (0.4-0.64) |
| Congenital musculoskeletal and limb anomalies | Morocco | 16492.1 (11332.6-23908.9) | 43.5 (29.9-63.1) | 13296.8 (9067.5-19027.8) | 43.4 (29.6-62.2) | 0.03 (-0.05-0.1) |
| Congenital musculoskeletal and limb anomalies | Mozambique | 13672.5 (9259.4-19826.4) | 46.7 (31.6-67.7) | 25839.9 (17472.7-36942) | 48.7 (33-69.7) | 0.21 (0.11-0.3) |
| Congenital musculoskeletal and limb anomalies | Namibia | 770.9 (535.4-1113.8) | 31.3 (21.8-45.3) | 835.8 (556.4-1221.8) | 30.4 (20.2-44.4) | -0.1 (-0.18--0.03) |
| Congenital musculoskeletal and limb anomalies | Nauru | 6.3 (4.3-9.2) | 37.3 (25.3-54.2) | 5.3 (3.6-7.6) | 38.1 (26.2-54.9) | 0.29 (0.13-0.45) |
| Congenital musculoskeletal and limb anomalies | Nepal | 13237.7 (8907.8-18951.1) | 35.1 (23.6-50.2) | 10223.4 (6849-14878) | 33.2 (22.2-48.3) | -0.33 (-0.39--0.27) |
| Congenital musculoskeletal and limb anomalies | New Zealand | 1796.6 (1247.3-2602.3) | 62 (43-89.8) | 1584.5 (1225.3-2021.5) | 53.6 (41.4-68.3) | -0.91 (-1.16--0.67) |
| Congenital musculoskeletal and limb anomalies | Niger | 11051.9 (7493.6-16208.8) | 51.7 (35-75.8) | 30646.9 (20727.2-44250.3) | 54.1 (36.6-78.1) | -0.01 (-0.16-0.14) |
| Congenital musculoskeletal and limb anomalies | Nigeria | 79475.6 (55008.5-111948.2) | 40.6 (28.1-57.1) | 170560.3 (117846.1-242681.1) | 43.1 (29.8-61.3) | 0.17 (0.13-0.22) |
| Congenital musculoskeletal and limb anomalies | Niue | 0.5 (0.3-0.7) | 23 (15.6-33) | 0.3 (0.2-0.4) | 25.1 (17-36.8) | 0.18 (0.13-0.23) |
| Congenital musculoskeletal and limb anomalies | North Macedonia | 497 (344.2-727.2) | 30.5 (21.1-44.6) | 267.6 (184.6-380.2) | 29.5 (20.3-41.9) | -0.35 (-0.44--0.26) |
| Congenital musculoskeletal and limb anomalies | Northern Mariana Islands | 9.3 (6.4-14.1) | 15.8 (10.8-23.9) | 5.2 (3.5-8) | 18.3 (12.3-27.8) | 0.38 (0.32-0.45) |
| Congenital musculoskeletal and limb anomalies | Oman | 1165 (779-1719.6) | 33.8 (22.6-49.8) | 1132.7 (754.6-1682.1) | 29.8 (19.8-44.2) | -0.43 (-0.49--0.37) |
| Congenital musculoskeletal and limb anomalies | Pakistan | 67207.5 (47359.5-94565.9) | 33.3 (23.5-46.9) | 105291.7 (72628.1-143684.7) | 35.2 (24.3-48.1) | 0.32 (0.22-0.42) |
| Congenital musculoskeletal and limb anomalies | Palau | 3.6 (2.4-5.3) | 23.7 (16.1-35.4) | 2.2 (1.5-3.3) | 25.9 (17.6-38.3) | 0.3 (0.22-0.38) |
| Congenital musculoskeletal and limb anomalies | Palestine | 1002.9 (678.6-1484.5) | 23.7 (16-35.1) | 1362.5 (934.6-2006.2) | 23.6 (16.2-34.8) | 0.05 (-0.05-0.14) |
| Congenital musculoskeletal and limb anomalies | People's Democratic Republic of Algeria | 14087.3 (9817.5-20461.8) | 38.1 (26.5-55.3) | 14856.4 (9911.7-21703.2) | 34 (22.7-49.7) | -0.27 (-0.32--0.23) |
| Congenital musculoskeletal and limb anomalies | People's Republic of China | 492326 (337690.5-701398) | 44.5 (30.5-63.4) | 238561.2 (169545.2-338146.8) | 45 (32-63.8) | -0.05 (-0.18-0.08) |
| Congenital musculoskeletal and limb anomalies | Plurinational State of Bolivia | 5838.4 (4041.9-8485.7) | 53.4 (36.9-77.6) | 6151.8 (4142.7-8736.3) | 52.5 (35.4-74.6) | -0.03 (-0.09-0.04) |
| Congenital musculoskeletal and limb anomalies | Portuguese Republic | 2416.2 (1693-3377) | 43.6 (30.6-61) | 1715.7 (1277.1-2233.2) | 43.5 (32.4-56.6) | 0.24 (0.09-0.39) |
| Congenital musculoskeletal and limb anomalies | Principality of Andorra | 9.7 (6.8-13.6) | 38.3 (26.8-53.7) | 8.6 (6-12.2) | 38.9 (27.3-55) | 0.54 (0.33-0.76) |
| Congenital musculoskeletal and limb anomalies | Puerto Rico | 556.6 (380.4-819.1) | 17.7 (12.1-26) | 165.6 (111.8-244.9) | 18.8 (12.7-27.7) | 0.17 (0.09-0.26) |
| Congenital musculoskeletal and limb anomalies | Qatar | 243 (166.3-362.3) | 44.4 (30.4-66.2) | 658.3 (460.7-964.4) | 35.5 (24.8-52) | -1.18 (-1.31--1.05) |
| Congenital musculoskeletal and limb anomalies | Republic of Albania | 1316.4 (905.9-1815) | 33.9 (23.3-46.7) | 420.4 (288.8-596.9) | 31.5 (21.6-44.7) | -0.42 (-0.55--0.3) |
| Congenital musculoskeletal and limb anomalies | Republic of Armenia | 1214.1 (833-1811.3) | 33.4 (22.9-49.9) | 522.2 (356.1-768.6) | 31.5 (21.5-46.4) | -0.3 (-0.39--0.22) |
| Congenital musculoskeletal and limb anomalies | Republic of Austria | 1099.3 (844.8-1513.5) | 24.6 (18.9-33.9) | 1176.8 (815.3-1696.3) | 28.8 (19.9-41.5) | 0.5 (0.15-0.85) |
| Congenital musculoskeletal and limb anomalies | Republic of Azerbaijan | 4096.8 (2784-5994.2) | 46 (31.3-67.3) | 2892.2 (1961.1-4182.8) | 44.9 (30.4-64.9) | -0.22 (-0.36--0.07) |
| Congenital musculoskeletal and limb anomalies | Republic of Belarus | 2380.5 (1635.8-3531.8) | 34.8 (23.9-51.7) | 1260.8 (853.9-1831.6) | 31.6 (21.4-45.9) | -0.31 (-0.39--0.23) |
| Congenital musculoskeletal and limb anomalies | Republic of Bulgaria | 1437.9 (1022.8-2057) | 30.1 (21.4-43) | 896.8 (611.9-1269.7) | 32.1 (21.9-45.5) | 0.36 (0.25-0.47) |
| Congenital musculoskeletal and limb anomalies | Republic of Chile | 9536 (6434.5-13597.2) | 65.1 (43.9-92.8) | 6456.5 (4789.8-8578.7) | 66.2 (49.1-88) | 0.09 (0.02-0.16) |
| Congenital musculoskeletal and limb anomalies | Republic of Colombia | 20107.6 (13115.6-29472.3) | 45.5 (29.7-66.6) | 13388 (8927.6-19869.5) | 41.4 (27.6-61.4) | -0.5 (-0.58--0.41) |
| Congenital musculoskeletal and limb anomalies | Republic of Costa Rica | 1228 (800.1-1844.6) | 31.4 (20.4-47.1) | 896.2 (575.5-1314.8) | 34.1 (21.9-50.1) | 0.14 (0.05-0.24) |
| Congenital musculoskeletal and limb anomalies | Republic of Croatia | 584.2 (484.9-672.5) | 21.9 (18.2-25.2) | 320.4 (217.5-467.4) | 19 (12.9-27.6) | -0.64 (-0.7--0.58) |
| Congenital musculoskeletal and limb anomalies | Republic of Cuba | 1651 (1122.3-2437.5) | 19.3 (13.1-28.5) | 952.4 (655.1-1376.7) | 19.8 (13.6-28.6) | 0.05 (0-0.1) |
| Congenital musculoskeletal and limb anomalies | Republic of Cyprus | 234.9 (162.1-328.4) | 35.6 (24.5-49.7) | 210.6 (145.2-304.9) | 29 (20-41.9) | -0.73 (-0.88--0.58) |
| Congenital musculoskeletal and limb anomalies | Republic of Ecuador | 6413.4 (4730.9-8585.8) | 44.3 (32.7-59.3) | 5565.1 (4634-6589.6) | 36.1 (30.1-42.8) | -0.81 (-0.95--0.67) |
| Congenital musculoskeletal and limb anomalies | Republic of El Salvador | 4591.5 (3066.5-6633.9) | 54.9 (36.7-79.4) | 2663.7 (1768.7-4047.7) | 47.6 (31.6-72.4) | -0.73 (-0.84--0.63) |
| Congenital musculoskeletal and limb anomalies | Republic of Estonia | 322 (218.3-475) | 31.1 (21.1-45.9) | 178.3 (121.5-252.6) | 28.6 (19.5-40.5) | -0.48 (-0.6--0.37) |
| Congenital musculoskeletal and limb anomalies | Republic of Fiji | 261.6 (174.3-380) | 28.9 (19.3-42) | 261.3 (172-383.7) | 30.1 (19.8-44.2) | 0.26 (0.17-0.34) |
| Congenital musculoskeletal and limb anomalies | Republic of Finland | 791.5 (651.1-1078.9) | 25.1 (20.6-34.2) | 660.4 (458.3-944.4) | 28.4 (19.7-40.6) | 0.28 (0.22-0.35) |
| Congenital musculoskeletal and limb anomalies | Republic of Guatemala | 11800.8 (7981.1-17204.1) | 71 (48-103.6) | 10771.6 (7195.4-15479.8) | 75.6 (50.5-108.6) | 0.24 (0.13-0.34) |
| Congenital musculoskeletal and limb anomalies | Republic of Guyana | 575.5 (397.6-833.8) | 46 (31.8-66.6) | 352.9 (241.1-503.7) | 49.2 (33.6-70.3) | 0.09 (0.05-0.12) |
| Congenital musculoskeletal and limb anomalies | Republic of Haiti | 6761.4 (4775.3-9572) | 55.8 (39.4-79) | 9929.3 (7066.1-13781.5) | 60.6 (43.1-84) | 0.05 (-0.02-0.12) |
| Congenital musculoskeletal and limb anomalies | Republic of Honduras | 5577.4 (3694.7-8290.7) | 64.9 (43-96.5) | 6984.4 (4809-9952.4) | 65.4 (45-93.2) | -0.02 (-0.06-0.01) |
| Congenital musculoskeletal and limb anomalies | Republic of Iceland | 74.7 (51.8-104.8) | 33.5 (23.3-47.1) | 77.3 (54.7-106.8) | 34.8 (24.6-48.1) | 0.23 (0.13-0.33) |
| Congenital musculoskeletal and limb anomalies | Republic of Indonesia | 90651.7 (62406.5-128640.4) | 40.3 (27.7-57.1) | 80676.8 (56188.8-114180.6) | 38.1 (26.5-53.9) | -0.24 (-0.27--0.2) |
| Congenital musculoskeletal and limb anomalies | Republic of Iraq | 14440.2 (9736.8-21120.4) | 43.5 (29.3-63.6) | 16106.8 (10896.5-23657.9) | 40.5 (27.4-59.4) | -0.28 (-0.38--0.18) |
| Congenital musculoskeletal and limb anomalies | Republic of Italy | 14674.1 (10752.6-20257.9) | 54.5 (40-75.3) | 11922.8 (9205-15587.8) | 61.4 (47.4-80.3) | 0.46 (0.36-0.56) |
| Congenital musculoskeletal and limb anomalies | Republic of Kazakhstan | 7027.7 (4699-10184.4) | 39.6 (26.5-57.4) | 7125.8 (4964.1-10291) | 36.3 (25.3-52.4) | -0.41 (-0.59--0.23) |
| Congenital musculoskeletal and limb anomalies | Republic of Kiribati | 58.1 (39.6-80.9) | 44.8 (30.5-62.3) | 67.5 (46.9-96.3) | 48 (33.4-68.5) | 0.16 (0.12-0.2) |
| Congenital musculoskeletal and limb anomalies | Republic of Korea | 23349.7 (16681.8-32494.8) | 72.3 (51.7-100.6) | 7801.7 (5422.8-11270.8) | 60.7 (42.2-87.7) | -0.94 (-1.05--0.83) |
| Congenital musculoskeletal and limb anomalies | Republic of Latvia | 582.6 (388.6-848.5) | 33 (22-48) | 275 (190.7-398.2) | 33.9 (23.5-49.1) | 0.23 (0.15-0.31) |
| Congenital musculoskeletal and limb anomalies | Republic of Lithuania | 837.1 (567.5-1208.9) | 30.9 (20.9-44.6) | 375.2 (251.9-530.1) | 32.8 (22-46.3) | 0.58 (0.43-0.73) |
| Congenital musculoskeletal and limb anomalies | Republic of Maldives | 150.3 (101-221.8) | 35.3 (23.7-52.1) | 70.5 (47-105.7) | 24.4 (16.3-36.6) | -1.27 (-1.51--1.03) |
| Congenital musculoskeletal and limb anomalies | Republic of Malta | 76.4 (60.5-99.6) | 28.6 (22.6-37.3) | 61.6 (44.2-83.5) | 29.4 (21.1-39.9) | 0.26 (0.14-0.38) |
| Congenital musculoskeletal and limb anomalies | Republic of Moldova | 1569.4 (1060.8-2222.6) | 41.2 (27.8-58.3) | 540.8 (365.8-782.9) | 39.8 (26.9-57.7) | 0.01 (-0.05-0.07) |
| Congenital musculoskeletal and limb anomalies | Republic of Nicaragua | 3286.5 (2168.9-4919) | 48.1 (31.8-72) | 2932.6 (1922.4-4316.9) | 47.7 (31.2-70.2) | -0.23 (-0.36--0.1) |
| Congenital musculoskeletal and limb anomalies | Republic of Panama | 1109 (740.2-1633.6) | 39.1 (26.1-57.6) | 1350.6 (871.2-1988.3) | 39.8 (25.7-58.7) | 0.08 (-0.01-0.16) |
| Congenital musculoskeletal and limb anomalies | Republic of Paraguay | 1478.7 (989-2208.5) | 23.8 (15.9-35.6) | 1516 (980.5-2235.9) | 24.5 (15.8-36.1) | 0.36 (0.18-0.53) |
| Congenital musculoskeletal and limb anomalies | Republic of Peru | 12192.9 (8041.6-17780.3) | 39.5 (26-57.6) | 10241.2 (6885.6-15157.5) | 31.7 (21.3-46.9) | -0.75 (-0.87--0.63) |
| Congenital musculoskeletal and limb anomalies | Republic of Poland | 5678.4 (3921-8122.6) | 21.6 (14.9-30.9) | 3321.5 (2325.6-4703.9) | 20.2 (14.2-28.6) | -0.29 (-0.34--0.25) |
| Congenital musculoskeletal and limb anomalies | Republic of Serbia | 2576 (1729.5-3637.4) | 39 (26.2-55) | 1072.3 (757.5-1508.8) | 33.2 (23.5-46.7) | -1.41 (-1.86--0.96) |
| Congenital musculoskeletal and limb anomalies | Republic of Singapore | 1715.6 (1178.9-2408.7) | 70.8 (48.7-99.4) | 1465.8 (1004.2-2067.1) | 55.4 (37.9-78.1) | -0.74 (-0.92--0.57) |
| Congenital musculoskeletal and limb anomalies | Republic of Slovenia | 250.8 (174.2-351.4) | 23.4 (16.3-32.9) | 198.1 (141.8-283.4) | 22.2 (15.9-31.7) | -0.03 (-0.2-0.15) |
| Congenital musculoskeletal and limb anomalies | Republic of Suriname | 167.2 (114.1-240.7) | 38 (26-54.8) | 174.2 (120-252.9) | 40.8 (28.1-59.3) | 0.1 (0.02-0.17) |
| Congenital musculoskeletal and limb anomalies | Republic of Tajikistan | 5214.5 (3649.9-7690.8) | 52.1 (36.5-76.9) | 7542.2 (5319.3-10403.3) | 55.5 (39.1-76.5) | 0.37 (0.3-0.43) |
| Congenital musculoskeletal and limb anomalies | Republic of the Marshall Islands | 25.4 (17.1-36.5) | 35.3 (23.8-50.8) | 21.8 (14.4-31.4) | 39.7 (26.4-57.2) | 0.28 (0.2-0.37) |
| Congenital musculoskeletal and limb anomalies | Republic of the Philippines | 33377 (23148.4-47753.5) | 34.4 (23.9-49.2) | 39546.1 (27407.6-55967.2) | 36.3 (25.1-51.3) | 0.26 (0.18-0.34) |
| Congenital musculoskeletal and limb anomalies | Republic of the Union of Myanmar | 28328.1 (19118.2-39947) | 52.9 (35.7-74.5) | 23580.5 (16504.4-32984.4) | 45.8 (32.1-64.1) | -0.5 (-0.55--0.45) |
| Congenital musculoskeletal and limb anomalies | Republic of Trinidad and Tobago | 338.3 (235.7-490) | 29.5 (20.5-42.7) | 216.6 (150.1-311.4) | 30.3 (21-43.6) | -0.19 (-0.31--0.07) |
| Congenital musculoskeletal and limb anomalies | Republic of Uzbekistan | 15199.4 (10732.3-22091.6) | 44.6 (31.5-64.8) | 17783.3 (12157.4-25806.7) | 46.5 (31.8-67.4) | 0.04 (-0.03-0.1) |
| Congenital musculoskeletal and limb anomalies | Republic of Vanuatu | 106 (70.9-152.3) | 36.4 (24.3-52.2) | 170.2 (115.6-248.4) | 40.5 (27.5-59.1) | 0.39 (0.32-0.46) |
| Congenital musculoskeletal and limb anomalies | Romania | 4556 (3108.1-6391.2) | 31.4 (21.4-44) | 2607.4 (1765.9-3635.5) | 30.4 (20.6-42.4) | -0.17 (-0.21--0.13) |
| Congenital musculoskeletal and limb anomalies | Russian Federation | 46901.1 (32693.2-65873.5) | 49.3 (34.4-69.2) | 29072.5 (20331.3-41121.4) | 44.7 (31.2-63.2) | -0.5 (-0.6--0.4) |
| Congenital musculoskeletal and limb anomalies | Rwanda | 6986.1 (4660.6-10101.6) | 45.8 (30.5-66.2) | 7620.1 (4964-10842.3) | 43 (28-61.2) | -0.41 (-0.52--0.3) |
| Congenital musculoskeletal and limb anomalies | Saint Kitts and Nevis | 15.6 (10.7-22.7) | 34.9 (23.9-50.9) | 8.6 (6-12.7) | 31 (21.4-45.6) | -0.5 (-0.59--0.41) |
| Congenital musculoskeletal and limb anomalies | Saint Lucia | 49.8 (33.8-74.1) | 29.1 (19.7-43.3) | 24.1 (16.2-35.4) | 29.8 (20-43.7) | -0.07 (-0.14-0.01) |
| Congenital musculoskeletal and limb anomalies | Saint Vincent and the Grenadines | 35.1 (23.7-50.9) | 28.4 (19.2-41.2) | 20.4 (14-29.1) | 32.6 (22.3-46.4) | 0.24 (0.11-0.36) |
| Congenital musculoskeletal and limb anomalies | San Marino | 3.5 (2.3-5) | 29.8 (19.9-42.9) | 4 (2.7-5.7) | 36.5 (24.8-51.8) | 0.72 (0.65-0.79) |
| Congenital musculoskeletal and limb anomalies | Sao Tome and Principe | 67.3 (44.4-99.9) | 30.3 (20-45) | 66.6 (44.5-98.6) | 28.1 (18.8-41.6) | -0.28 (-0.35--0.2) |
| Congenital musculoskeletal and limb anomalies | Saudi Arabia | 9559.1 (6401.2-13904.5) | 38.9 (26.1-56.6) | 6863.6 (4636.8-10267.4) | 30.8 (20.8-46.1) | -0.86 (-0.91--0.8) |
| Congenital musculoskeletal and limb anomalies | Senegal | 6383.2 (4174.2-9633.5) | 38.5 (25.2-58.1) | 8926.7 (5842.9-13266.6) | 38.7 (25.3-57.5) | 0.14 (0.07-0.21) |
| Congenital musculoskeletal and limb anomalies | Seychelles | 20.6 (13.2-30.9) | 26 (16.6-39) | 18.5 (12.3-27.1) | 24.4 (16.2-35.8) | -0.21 (-0.31--0.11) |
| Congenital musculoskeletal and limb anomalies | Sierra Leone | 4155 (2754.2-5764.2) | 42.7 (28.3-59.2) | 6595 (4457.2-9732.5) | 45.6 (30.8-67.2) | 0.2 (0.13-0.28) |
| Congenital musculoskeletal and limb anomalies | Slovak Republic | 968.8 (683.3-1370.4) | 25.4 (17.9-35.9) | 715.3 (493.5-1029) | 26.7 (18.4-38.4) | 0.24 (0.17-0.31) |
| Congenital musculoskeletal and limb anomalies | Socialist Republic of Viet Nam | 28803.6 (19253.8-41655.9) | 31 (20.7-44.8) | 18217.8 (12513.9-27837.4) | 24.5 (16.8-37.4) | -0.87 (-0.94--0.8) |
| Congenital musculoskeletal and limb anomalies | Solomon Islands | 272.6 (187.5-388.7) | 41.7 (28.6-59.4) | 424.3 (293.7-618.1) | 43.4 (30.1-63.2) | 0.13 (0.07-0.19) |
| Congenital musculoskeletal and limb anomalies | Somalia | 9141 (6277.7-12912) | 49.3 (33.8-69.6) | 25186.9 (16842-35734.7) | 54.7 (36.5-77.5) | 0.33 (0.26-0.4) |
| Congenital musculoskeletal and limb anomalies | South Africa | 12679 (8925.7-18126.2) | 25.2 (17.7-36) | 12324.1 (8614-17631.3) | 26.1 (18.2-37.3) | -0.02 (-0.11-0.06) |
| Congenital musculoskeletal and limb anomalies | South Sudan | 5466.9 (3790.9-7802.3) | 44.5 (30.9-63.6) | 8633.5 (5650.6-12475.1) | 47.1 (30.8-68.1) | 0.23 (0.11-0.35) |
| Congenital musculoskeletal and limb anomalies | State of Israel | 1908.4 (1311.8-2661.4) | 37.7 (25.9-52.6) | 3176.6 (2184.3-4530.9) | 35.8 (24.6-51.1) | -0.08 (-0.12--0.04) |
| Congenital musculoskeletal and limb anomalies | State of Kuwait | 363.1 (246-533.8) | 22.5 (15.3-33.1) | 501.1 (337.6-768.3) | 20.9 (14.1-32.1) | -0.24 (-0.35--0.13) |
| Congenital musculoskeletal and limb anomalies | Sudan | 26078.6 (17724.8-36690.9) | 62.2 (42.3-87.5) | 30485.7 (21061-42606.9) | 55.3 (38.2-77.3) | -0.36 (-0.42--0.3) |
| Congenital musculoskeletal and limb anomalies | Swiss Confederation | 983.5 (831.2-1205) | 24.3 (20.5-29.8) | 1155.1 (827.4-1599.9) | 27.3 (19.6-37.8) | 0.29 (-0.18-0.75) |
| Congenital musculoskeletal and limb anomalies | Syrian Arab Republic | 8092.6 (5458.2-11729) | 36.7 (24.8-53.2) | 3239 (2182.3-4870.9) | 34.3 (23.1-51.6) | -0.1 (-0.27-0.07) |
| Congenital musculoskeletal and limb anomalies | Taiwan (Province of China) | 2793.9 (1829.9-4316.5) | 17.9 (11.7-27.6) | 1612.2 (1073.6-2394.4) | 21.3 (14.2-31.6) | 0.61 (0.43-0.79) |
| Congenital musculoskeletal and limb anomalies | Togo | 2889.2 (1902-4269.3) | 37.6 (24.7-55.5) | 4641 (3094.5-6722.8) | 39.9 (26.6-57.8) | 0.16 (0.1-0.22) |
| Congenital musculoskeletal and limb anomalies | Tokelau | 0.5 (0.3-0.8) | 29.2 (19.4-42.7) | 0.2 (0.2-0.4) | 28.2 (18.9-43) | -0.34 (-0.45--0.23) |
| Congenital musculoskeletal and limb anomalies | Tunisia | 3188.4 (2130.2-4779.5) | 30.2 (20.2-45.3) | 2376.7 (1648.1-3487.5) | 29.6 (20.6-43.5) | -0.06 (-0.12-0) |
| Congenital musculoskeletal and limb anomalies | Turkey | 25280.5 (16901.8-36308.6) | 35.6 (23.8-51.1) | 15179.7 (10501.1-21628.6) | 31.3 (21.7-44.6) | -0.47 (-0.55--0.4) |
| Congenital musculoskeletal and limb anomalies | Turkmenistan | 2848.5 (1929.3-4175.9) | 47.3 (32-69.4) | 2597.4 (1776.6-3724.4) | 49.1 (33.6-70.4) | 0.08 (0-0.15) |
| Congenital musculoskeletal and limb anomalies | Tuvalu | 7 (4.8-9.8) | 42.4 (29-59.9) | 4.9 (3.3-7) | 38 (25.7-54.7) | -0.33 (-0.44--0.22) |
| Congenital musculoskeletal and limb anomalies | Uganda | 17980 (12199-25835.1) | 40.7 (27.6-58.5) | 31661.3 (21473.2-45005.2) | 41.9 (28.4-59.5) | 0.01 (-0.08-0.09) |
| Congenital musculoskeletal and limb anomalies | Ukraine | 9289.6 (6419.9-13260.4) | 28.8 (19.9-41.2) | 4331.1 (2928.4-6269.9) | 32.8 (22.2-47.5) | 0.61 (0.49-0.72) |
| Congenital musculoskeletal and limb anomalies | United Arab Emirates | 810.1 (562.4-1207.1) | 34.9 (24.2-52) | 1154 (785.8-1646.4) | 31.5 (21.4-44.9) | -0.05 (-0.2-0.11) |
| Congenital musculoskeletal and limb anomalies | United Kingdom of Great Britain and Northern Ireland | 14003.7 (9951.3-19536) | 36.6 (26-51.1) | 12145.7 (8643.5-17017.9) | 36.3 (25.8-50.9) | -0.02 (-0.06-0.02) |
| Congenital musculoskeletal and limb anomalies | United Mexican States | 81136.5 (56098.2-114260.7) | 66.6 (46.1-93.8) | 51385.4 (36731.1-72292.2) | 56.7 (40.5-79.8) | -0.78 (-0.89--0.66) |
| Congenital musculoskeletal and limb anomalies | United Republic of Tanzania | 22101.4 (15225.8-31332.9) | 38.3 (26.4-54.3) | 36752.9 (24735.9-55258.3) | 40.7 (27.4-61.2) | 0.14 (0.09-0.18) |
| Congenital musculoskeletal and limb anomalies | United States of America | 71664 (49882.5-98900.6) | 35.8 (24.9-49.4) | 56061 (41920.6-73984) | 31.6 (23.7-41.8) | 0.52 (0.18-0.87) |
| Congenital musculoskeletal and limb anomalies | United States Virgin Islands | 26.6 (18.3-38.9) | 23.9 (16.4-34.9) | 9.2 (6.5-13.2) | 26.3 (18.7-37.8) | 0.57 (0.47-0.67) |
| Congenital musculoskeletal and limb anomalies | Yemen | 17210.3 (12019.7-23858.8) | 54.6 (38.1-75.7) | 23740.8 (17007.4-33888) | 51 (36.5-72.7) | -0.23 (-0.28--0.18) |
| Congenital musculoskeletal and limb anomalies | Zambia | 8038.5 (5483.7-11360.7) | 43.9 (30-62.1) | 12433.4 (8345.4-17514.2) | 42.9 (28.8-60.5) | -0.22 (-0.33--0.12) |
| Congenital musculoskeletal and limb anomalies | Zimbabwe | 5723 (3950.2-8285.4) | 30.8 (21.3-44.6) | 8829.1 (6040-12614.5) | 39.4 (27-56.3) | 0.96 (0.88-1.04) |

Table S2. The congenital musculoskeletal and limb anomalies cases and age standardized mortality from 1990 to 2021 in 204 countries and territories.

| Cause | Location | 1990 | | 2021 | | 1990-2021 |
| --- | --- | --- | --- | --- | --- | --- |
|  |  | Number of deaths  No. ×10^3^ (95% UI) | ASMR per 100,000  No. (95% UI) | Number of deaths  No. ×10^3^ (95% UI) | ASMR per 100,000  No. (95% UI) | EAPC  No. (95% CI) |
| Congenital musculoskeletal and limb anomalies | Afghanistan | 272 (45.5-561.6) | 1.6 (0.3-3.2) | 442.4 (168.7-791.9) | 0.9 (0.3-1.6) | -1.59 (-1.79--1.4) |
| Congenital musculoskeletal and limb anomalies | American Samoa | 0.1 (0-0.1) | 0.1 (0.1-0.1) | 0 (0-0) | 0.1 (0-0.1) | -0.19 (-0.5-0.11) |
| Congenital musculoskeletal and limb anomalies | Angola | 76.6 (35.4-154.6) | 0.4 (0.2-0.7) | 134.1 (75.9-265.6) | 0.2 (0.1-0.5) | -1.1 (-1.21--0.98) |
| Congenital musculoskeletal and limb anomalies | Antigua and Barbuda | 0.1 (0.1-0.1) | 0.1 (0.1-0.2) | 0.1 (0-0.1) | 0.1 (0.1-0.2) | -0.26 (-0.42--0.11) |
| Congenital musculoskeletal and limb anomalies | Arab Republic of Egypt | 861.5 (265-1437.4) | 1 (0.3-1.7) | 357.8 (246.8-492.9) | 0.3 (0.2-0.4) | -3.93 (-4.07--3.8) |
| Congenital musculoskeletal and limb anomalies | Argentine Republic | 46.5 (37.3-61.1) | 0.1 (0.1-0.2) | 39.1 (29.1-53) | 0.1 (0.1-0.2) | 0.24 (0.06-0.42) |
| Congenital musculoskeletal and limb anomalies | Australia | 28.5 (21.9-32.9) | 0.2 (0.2-0.2) | 13.8 (10.7-17.4) | 0.1 (0.1-0.1) | -3.1 (-3.3--2.9) |
| Congenital musculoskeletal and limb anomalies | Bangladesh | 945.9 (382-1967.2) | 0.5 (0.2-1) | 335.7 (150.2-556) | 0.2 (0.1-0.4) | -2.33 (-2.46--2.19) |
| Congenital musculoskeletal and limb anomalies | Barbados | 0.5 (0.4-0.6) | 0.2 (0.2-0.3) | 0.3 (0.2-0.4) | 0.2 (0.1-0.3) | -0.56 (-0.79--0.34) |
| Congenital musculoskeletal and limb anomalies | Belize | 0.5 (0.4-0.7) | 0.2 (0.1-0.2) | 0.5 (0.4-0.7) | 0.1 (0.1-0.2) | -0.35 (-0.56--0.14) |
| Congenital musculoskeletal and limb anomalies | Benin | 35.6 (19.3-60) | 0.4 (0.2-0.6) | 66.3 (40.2-106.1) | 0.3 (0.2-0.5) | -0.51 (-0.67--0.35) |
| Congenital musculoskeletal and limb anomalies | Bermuda | 0.1 (0-0.1) | 0.1 (0.1-0.2) | 0 (0-0) | 0.1 (0-0.1) | -2.16 (-2.36--1.95) |
| Congenital musculoskeletal and limb anomalies | Bhutan | 3.4 (1.3-8.6) | 0.4 (0.1-0.9) | 1.3 (0.6-2) | 0.2 (0.1-0.3) | -1.99 (-2.13--1.85) |
| Congenital musculoskeletal and limb anomalies | Bolivarian Republic of Venezuela | 26.1 (18.1-32) | 0.1 (0.1-0.1) | 23.5 (15.8-34.9) | 0.1 (0.1-0.2) | -0.15 (-0.48-0.19) |
| Congenital musculoskeletal and limb anomalies | Bosnia and Herzegovina | 4.4 (2-9.1) | 0.1 (0.1-0.3) | 1 (0.6-1.7) | 0.1 (0-0.1) | -2.3 (-2.62--1.99) |
| Congenital musculoskeletal and limb anomalies | Botswana | 7.6 (3.8-12.1) | 0.4 (0.2-0.6) | 8.2 (4.4-13.5) | 0.4 (0.2-0.6) | 0.39 (0.24-0.53) |
| Congenital musculoskeletal and limb anomalies | Brunei Darussalam | 2.2 (1.5-3.1) | 0.7 (0.5-0.9) | 1.5 (1.1-2.1) | 0.5 (0.3-0.7) | -0.6 (-0.76--0.44) |
| Congenital musculoskeletal and limb anomalies | Burkina Faso | 84.2 (45-138.9) | 0.4 (0.2-0.7) | 154.5 (97.6-245.5) | 0.4 (0.2-0.6) | -0.14 (-0.3-0.03) |
| Congenital musculoskeletal and limb anomalies | Burundi | 35.3 (15.6-71) | 0.3 (0.1-0.6) | 51.1 (28.4-85.4) | 0.2 (0.1-0.4) | -0.5 (-0.76--0.25) |
| Congenital musculoskeletal and limb anomalies | Cabo Verde | 1.2 (0.7-2.3) | 0.2 (0.1-0.4) | 0.6 (0.2-1.3) | 0.1 (0-0.3) | -1.65 (-1.73--1.56) |
| Congenital musculoskeletal and limb anomalies | Cameroon | 56.8 (32.2-92.9) | 0.3 (0.2-0.5) | 127.8 (77.3-219.6) | 0.3 (0.2-0.5) | 0.24 (0.1-0.38) |
| Congenital musculoskeletal and limb anomalies | Canada | 38.2 (32.9-47.9) | 0.2 (0.2-0.2) | 26.7 (20-32) | 0.1 (0.1-0.1) | -1.09 (-1.33--0.85) |
| Congenital musculoskeletal and limb anomalies | Central African Republic | 20.7 (9.6-43.1) | 0.4 (0.2-0.8) | 31.9 (16.8-57.7) | 0.4 (0.2-0.7) | 0.22 (0.11-0.33) |
| Congenital musculoskeletal and limb anomalies | Chad | 40.6 (19.4-78.2) | 0.3 (0.2-0.6) | 118.3 (67.9-199.3) | 0.4 (0.2-0.6) | 0.41 (0.35-0.48) |
| Congenital musculoskeletal and limb anomalies | Commonwealth of Dominica | 0.3 (0.2-0.4) | 0.3 (0.2-0.5) | 0.3 (0.2-0.4) | 0.7 (0.5-1) | 2.87 (2.61-3.14) |
| Congenital musculoskeletal and limb anomalies | Commonwealth of the Bahamas | 0.5 (0.3-0.7) | 0.2 (0.1-0.3) | 0.4 (0.2-0.6) | 0.2 (0.1-0.2) | -0.51 (-0.63--0.38) |
| Congenital musculoskeletal and limb anomalies | Comoros | 3 (1.4-5.6) | 0.3 (0.2-0.6) | 2.3 (1.2-4.3) | 0.3 (0.2-0.5) | -0.39 (-0.57--0.21) |
| Congenital musculoskeletal and limb anomalies | Congo | 10.7 (6.2-19.2) | 0.3 (0.2-0.5) | 12.2 (6.6-26.4) | 0.2 (0.1-0.4) | -0.85 (-0.98--0.72) |
| Congenital musculoskeletal and limb anomalies | Cook Islands | 0 (0-0) | 0.1 (0-0.1) | 0 (0-0) | 0 (0-0.1) | -4.33 (-4.78--3.88) |
| Congenital musculoskeletal and limb anomalies | C么te d'Ivoire | 73.5 (42.6-121.6) | 0.3 (0.2-0.5) | 134.2 (77.1-256.2) | 0.3 (0.2-0.6) | 0.29 (0.15-0.42) |
| Congenital musculoskeletal and limb anomalies | Czech Republic | 13 (9-16) | 0.2 (0.1-0.2) | 1.8 (1.2-2.6) | 0 (0-0) | -5.5 (-6.14--4.85) |
| Congenital musculoskeletal and limb anomalies | Democratic People's Republic of Korea | 19.5 (10.6-34.6) | 0.1 (0-0.1) | 8.9 (5.7-13.7) | 0 (0-0.1) | -1.52 (-1.72--1.32) |
| Congenital musculoskeletal and limb anomalies | Democratic Republic of the Congo | 255.6 (134.6-453.2) | 0.3 (0.2-0.6) | 291.2 (171.1-508.1) | 0.2 (0.1-0.4) | -0.93 (-1.08--0.78) |
| Congenital musculoskeletal and limb anomalies | Democratic Republic of Timor-Leste | 4.7 (0.8-10.8) | 0.3 (0.1-0.7) | 3.9 (1.7-6.5) | 0.2 (0.1-0.3) | -1.39 (-1.47--1.31) |
| Congenital musculoskeletal and limb anomalies | Democratic Socialist Republic of Sri Lanka | 15.8 (8.4-26.9) | 0.1 (0-0.2) | 6.8 (4.2-10.5) | 0 (0-0.1) | -2.36 (-2.64--2.08) |
| Congenital musculoskeletal and limb anomalies | Djibouti | 1.8 (1.1-3.7) | 0.3 (0.2-0.5) | 3.2 (1.4-6.9) | 0.2 (0.1-0.5) | -0.4 (-0.65--0.14) |
| Congenital musculoskeletal and limb anomalies | Dominican Republic | 9.1 (5.5-16) | 0.1 (0.1-0.2) | 6.1 (3.5-12.5) | 0.1 (0-0.1) | -0.72 (-1.05--0.39) |
| Congenital musculoskeletal and limb anomalies | Eastern Republic of Uruguay | 4.9 (4-6.7) | 0.2 (0.1-0.2) | 2.9 (2-3.9) | 0.1 (0.1-0.2) | -0.57 (-0.79--0.36) |
| Congenital musculoskeletal and limb anomalies | Equatorial Guinea | 2.7 (1.3-5.2) | 0.3 (0.1-0.6) | 3.7 (1.6-11) | 0.2 (0.1-0.6) | -1.49 (-1.63--1.34) |
| Congenital musculoskeletal and limb anomalies | Eritrea | 18.3 (8.3-37.8) | 0.3 (0.1-0.6) | 22.7 (12.8-42.3) | 0.3 (0.1-0.5) | -0.27 (-0.43--0.1) |
| Congenital musculoskeletal and limb anomalies | Eswatini | 5.8 (2.8-10.1) | 0.4 (0.2-0.7) | 4.5 (2.2-7.6) | 0.3 (0.2-0.6) | -0.29 (-0.41--0.16) |
| Congenital musculoskeletal and limb anomalies | Ethiopia | 362.3 (140-747.9) | 0.4 (0.1-0.7) | 374.7 (219.7-674.1) | 0.2 (0.1-0.4) | -1.31 (-1.35--1.27) |
| Congenital musculoskeletal and limb anomalies | Federal Republic of Germany | 108.3 (88.1-135.1) | 0.2 (0.2-0.3) | 39.2 (32.1-48.5) | 0.1 (0.1-0.1) | -2.96 (-3.36--2.55) |
| Congenital musculoskeletal and limb anomalies | Federated States of Micronesia | 0.2 (0.1-0.4) | 0.2 (0.1-0.3) | 0.1 (0-0.1) | 0.1 (0-0.1) | -2 (-2.13--1.87) |
| Congenital musculoskeletal and limb anomalies | Federative Republic of Brazil | 244.7 (205.2-289.7) | 0.2 (0.1-0.2) | 232.4 (184.7-281.8) | 0.1 (0.1-0.2) | 0.04 (-0.21-0.28) |
| Congenital musculoskeletal and limb anomalies | French Republic | 50.2 (41.7-59.6) | 0.1 (0.1-0.1) | 23.5 (18.7-32.7) | 0.1 (0-0.1) | -2.28 (-2.64--1.92) |
| Congenital musculoskeletal and limb anomalies | Gabon | 3.4 (1.7-6.9) | 0.2 (0.1-0.4) | 3.7 (1.8-9.9) | 0.2 (0.1-0.5) | -0.02 (-0.26-0.22) |
| Congenital musculoskeletal and limb anomalies | Gambia | 4.7 (2.7-8.8) | 0.3 (0.2-0.5) | 7.8 (4.2-14.4) | 0.2 (0.1-0.4) | -0.21 (-0.34--0.07) |
| Congenital musculoskeletal and limb anomalies | Georgia | 2 (1.4-3.5) | 0 (0-0.1) | 0.6 (0.4-1.4) | 0 (0-0.1) | -1.16 (-1.75--0.58) |
| Congenital musculoskeletal and limb anomalies | Ghana | 67.9 (40-110.2) | 0.3 (0.2-0.5) | 97.5 (54.8-191.4) | 0.2 (0.1-0.4) | -0.03 (-0.2-0.13) |
| Congenital musculoskeletal and limb anomalies | Global | 18901.9 (10324.4-30380.6) | 0.3 (0.2-0.5) | 13599.8 (10506.2-18111.4) | 0.2 (0.2-0.3) | -1.15 (-1.18--1.11) |
| Congenital musculoskeletal and limb anomalies | Grand Duchy of Luxembourg | 1.2 (0.7-1.4) | 0.4 (0.3-0.5) | 0.5 (0.3-0.7) | 0.1 (0.1-0.2) | -3.89 (-4.54--3.24) |
| Congenital musculoskeletal and limb anomalies | Greenland | 0.2 (0.1-0.3) | 0.3 (0.2-0.5) | 0.1 (0-0.1) | 0.1 (0.1-0.2) | -2.47 (-2.59--2.34) |
| Congenital musculoskeletal and limb anomalies | Grenada | 0.2 (0.1-0.2) | 0.2 (0.1-0.2) | 0.1 (0.1-0.1) | 0.1 (0.1-0.1) | -0.42 (-0.61--0.22) |
| Congenital musculoskeletal and limb anomalies | Guam | 0.1 (0.1-0.2) | 0.1 (0-0.1) | 0.1 (0.1-0.1) | 0.1 (0-0.1) | 0.82 (0.51-1.14) |
| Congenital musculoskeletal and limb anomalies | Guinea | 55.4 (25.3-95.1) | 0.5 (0.2-0.8) | 78.8 (44-132.6) | 0.4 (0.2-0.6) | -0.39 (-0.5--0.28) |
| Congenital musculoskeletal and limb anomalies | Guinea-Bissau | 8.3 (4.3-13.7) | 0.5 (0.2-0.8) | 10.5 (5.5-18.8) | 0.4 (0.2-0.6) | -0.52 (-0.71--0.33) |
| Congenital musculoskeletal and limb anomalies | Hashemite Kingdom of Jordan | 33.4 (21.6-51.4) | 0.6 (0.4-0.8) | 23.6 (16.8-33) | 0.2 (0.1-0.3) | -3.34 (-3.54--3.15) |
| Congenital musculoskeletal and limb anomalies | Hellenic Republic | 6.5 (5.4-8.8) | 0.1 (0.1-0.1) | 2.4 (1.8-4.1) | 0 (0-0.1) | -2.4 (-2.9--1.89) |
| Congenital musculoskeletal and limb anomalies | Hungary | 11.9 (8.8-17.1) | 0.2 (0.1-0.3) | 3.1 (2.3-4.1) | 0.1 (0-0.1) | -3.3 (-3.47--3.13) |
| Congenital musculoskeletal and limb anomalies | Independent State of Papua New Guinea | 16.2 (4.3-37.9) | 0.2 (0.1-0.6) | 32.2 (12.9-69.6) | 0.2 (0.1-0.4) | -0.51 (-0.59--0.42) |
| Congenital musculoskeletal and limb anomalies | Independent State of Samoa | 0.3 (0.2-0.5) | 0.1 (0.1-0.2) | 0.2 (0.1-0.3) | 0.1 (0-0.1) | -1.42 (-1.58--1.26) |
| Congenital musculoskeletal and limb anomalies | India | 3979.3 (1377.8-8289.2) | 0.4 (0.1-0.7) | 2421.3 (1430.7-4255.9) | 0.2 (0.1-0.4) | -1.52 (-1.57--1.46) |
| Congenital musculoskeletal and limb anomalies | Ireland | 10 (7.8-11.6) | 0.3 (0.3-0.4) | 3.6 (2.6-4.5) | 0.1 (0.1-0.1) | -3.38 (-3.54--3.21) |
| Congenital musculoskeletal and limb anomalies | Islamic Republic of Iran | 772.6 (406.9-1197.7) | 1 (0.5-1.6) | 118.2 (83.2-150.7) | 0.2 (0.1-0.2) | -4.68 (-5.24--4.11) |
| Congenital musculoskeletal and limb anomalies | Jamaica | 4.2 (3.3-5.4) | 0.2 (0.1-0.2) | 2.1 (1.5-3) | 0.1 (0.1-0.2) | -0.63 (-0.87--0.4) |
| Congenital musculoskeletal and limb anomalies | Japan | 65 (56.5-98) | 0.1 (0.1-0.1) | 34.2 (20.7-55.8) | 0.1 (0-0.1) | -1.09 (-1.27--0.91) |
| Congenital musculoskeletal and limb anomalies | Kenya | 89.2 (54-161.1) | 0.2 (0.1-0.4) | 106.5 (43.6-238.6) | 0.2 (0.1-0.4) | 0.31 (0.08-0.55) |
| Congenital musculoskeletal and limb anomalies | Kingdom of Bahrain | 4.3 (3.1-6) | 0.7 (0.5-1) | 3 (2.1-3.9) | 0.3 (0.2-0.3) | -2.8 (-3.05--2.56) |
| Congenital musculoskeletal and limb anomalies | Kingdom of Belgium | 14.4 (12.1-18.2) | 0.2 (0.2-0.3) | 6 (4.7-7.5) | 0.1 (0.1-0.1) | -3.24 (-3.54--2.94) |
| Congenital musculoskeletal and limb anomalies | Kingdom of Cambodia | 73.5 (10.6-173.4) | 0.4 (0.1-0.9) | 33.9 (14.6-67.4) | 0.2 (0.1-0.4) | -2.29 (-2.43--2.15) |
| Congenital musculoskeletal and limb anomalies | Kingdom of Denmark | 9.9 (8.1-12.3) | 0.3 (0.2-0.4) | 3.2 (2.6-3.9) | 0.1 (0.1-0.1) | -3.89 (-4.35--3.43) |
| Congenital musculoskeletal and limb anomalies | Kingdom of Norway | 2.7 (2.3-4) | 0.1 (0.1-0.1) | 2.8 (1.2-3.5) | 0.1 (0-0.1) | -1.54 (-3.58-0.55) |
| Congenital musculoskeletal and limb anomalies | Kingdom of Spain | 37.1 (31.4-44.6) | 0.2 (0.1-0.2) | 12 (8.8-15.4) | 0 (0-0.1) | -3.89 (-4.27--3.5) |
| Congenital musculoskeletal and limb anomalies | Kingdom of Sweden | 10.8 (8.2-12.6) | 0.2 (0.1-0.2) | 4.9 (3.7-6.1) | 0.1 (0.1-0.1) | -2.05 (-2.6--1.5) |
| Congenital musculoskeletal and limb anomalies | Kingdom of Thailand | 186.5 (106.2-323.9) | 0.4 (0.2-0.6) | 34.6 (20.9-45.1) | 0.1 (0.1-0.2) | -3.93 (-4.18--3.69) |
| Congenital musculoskeletal and limb anomalies | Kingdom of the Netherlands | 28.9 (23.3-33.6) | 0.3 (0.2-0.3) | 11.1 (9.2-13.8) | 0.1 (0.1-0.1) | -3.26 (-3.58--2.95) |
| Congenital musculoskeletal and limb anomalies | Kingdom of Tonga | 0.1 (0.1-0.2) | 0.1 (0.1-0.1) | 0.1 (0-0.1) | 0.1 (0-0.1) | -1.16 (-1.23--1.1) |
| Congenital musculoskeletal and limb anomalies | Kyrgyz Republic | 7.5 (5.3-10.6) | 0.1 (0.1-0.2) | 4.9 (3.5-7.3) | 0.1 (0-0.1) | -1.64 (-2.04--1.25) |
| Congenital musculoskeletal and limb anomalies | Lao People's Democratic Republic | 30.6 (3.8-79.8) | 0.4 (0.1-1) | 20.5 (8.1-42.3) | 0.2 (0.1-0.5) | -1.52 (-1.67--1.38) |
| Congenital musculoskeletal and limb anomalies | Lebanon | 6.1 (2.6-12.2) | 0.2 (0.1-0.3) | 2.9 (1.8-4.2) | 0.1 (0-0.1) | -2.85 (-2.99--2.72) |
| Congenital musculoskeletal and limb anomalies | Lesotho | 13.5 (6.3-26.1) | 0.5 (0.3-1) | 11.3 (6-19.3) | 0.6 (0.3-1) | 0.23 (0.11-0.36) |
| Congenital musculoskeletal and limb anomalies | Liberia | 20.8 (9.1-38.8) | 0.4 (0.2-0.8) | 20.5 (11.9-34) | 0.3 (0.2-0.5) | -1.06 (-1.36--0.76) |
| Congenital musculoskeletal and limb anomalies | Libya | 48.3 (32-69) | 0.8 (0.6-1.2) | 31.1 (21.9-46.1) | 0.6 (0.4-0.9) | -0.49 (-0.82--0.17) |
| Congenital musculoskeletal and limb anomalies | Madagascar | 69.2 (37.1-137.7) | 0.3 (0.2-0.6) | 113.3 (58-200.7) | 0.3 (0.1-0.5) | 0.29 (0.12-0.45) |
| Congenital musculoskeletal and limb anomalies | Malawi | 95 (47.8-180.5) | 0.4 (0.2-0.8) | 77.9 (39.5-165) | 0.3 (0.1-0.6) | -0.91 (-1.12--0.71) |
| Congenital musculoskeletal and limb anomalies | Malaysia | 90.4 (51.4-135.7) | 0.4 (0.2-0.6) | 62.4 (36.9-90.2) | 0.3 (0.2-0.4) | -0.23 (-0.79-0.32) |
| Congenital musculoskeletal and limb anomalies | Mali | 104.1 (57.9-185.3) | 0.6 (0.3-1) | 199.6 (100.3-358.5) | 0.5 (0.2-0.9) | -0.64 (-0.72--0.56) |
| Congenital musculoskeletal and limb anomalies | Mauritania | 10.8 (6-19.8) | 0.3 (0.2-0.5) | 13.7 (6.1-28.6) | 0.2 (0.1-0.5) | -0.72 (-0.88--0.57) |
| Congenital musculoskeletal and limb anomalies | Mauritius | 1.4 (1-2.2) | 0.1 (0.1-0.2) | 0.8 (0.5-1.5) | 0.1 (0.1-0.2) | 0.38 (0-0.75) |
| Congenital musculoskeletal and limb anomalies | Monaco | 0 (0-0) | 0.2 (0.1-0.3) | 0 (0-0) | 0.1 (0.1-0.2) | -2.5 (-2.79--2.2) |
| Congenital musculoskeletal and limb anomalies | Mongolia | 13.6 (5.9-25.4) | 0.4 (0.2-0.8) | 3.2 (1.9-5) | 0.1 (0.1-0.1) | -5.45 (-5.91--4.98) |
| Congenital musculoskeletal and limb anomalies | Montenegro | 0.8 (0.5-1.3) | 0.2 (0.1-0.3) | 0.1 (0.1-0.2) | 0 (0-0.1) | -5.19 (-5.59--4.8) |
| Congenital musculoskeletal and limb anomalies | Morocco | 135.6 (74.9-220) | 0.4 (0.2-0.6) | 42.8 (20.9-108.3) | 0.1 (0.1-0.3) | -3.37 (-3.54--3.21) |
| Congenital musculoskeletal and limb anomalies | Mozambique | 111.5 (45.4-238.1) | 0.4 (0.2-0.9) | 150.9 (90.6-250.6) | 0.3 (0.2-0.5) | -0.54 (-0.73--0.36) |
| Congenital musculoskeletal and limb anomalies | Namibia | 8.9 (4.7-15.4) | 0.4 (0.2-0.6) | 8.2 (3.6-14.2) | 0.3 (0.1-0.5) | -0.25 (-0.43--0.06) |
| Congenital musculoskeletal and limb anomalies | Nauru | 0 (0-0) | 0.1 (0.1-0.2) | 0 (0-0) | 0.1 (0.1-0.2) | -0.63 (-0.9--0.36) |
| Congenital musculoskeletal and limb anomalies | Nepal | 92.1 (37.3-191.9) | 0.3 (0.1-0.5) | 29.8 (14.3-64.8) | 0.1 (0-0.2) | -3.51 (-3.71--3.32) |
| Congenital musculoskeletal and limb anomalies | New Zealand | 9.2 (7.4-10.5) | 0.3 (0.2-0.3) | 4.9 (3.9-6.2) | 0.1 (0.1-0.2) | -2.36 (-2.57--2.14) |
| Congenital musculoskeletal and limb anomalies | Niger | 65.9 (27.3-117.4) | 0.4 (0.2-0.7) | 133.4 (72.6-241.9) | 0.3 (0.2-0.5) | -1.18 (-1.37--0.98) |
| Congenital musculoskeletal and limb anomalies | Nigeria | 560.9 (332.4-961.9) | 0.3 (0.2-0.6) | 1371 (857.5-2118.4) | 0.4 (0.2-0.6) | 0.8 (0.66-0.94) |
| Congenital musculoskeletal and limb anomalies | Niue | 0 (0-0) | 0.1 (0.1-0.2) | 0 (0-0) | 0.3 (0.2-0.4) | 0.19 (-0.55-0.93) |
| Congenital musculoskeletal and limb anomalies | North Macedonia | 8.8 (5.1-14.5) | 0.5 (0.3-0.9) | 0.8 (0.5-1.2) | 0.1 (0-0.1) | -5.1 (-5.63--4.55) |
| Congenital musculoskeletal and limb anomalies | Northern Mariana Islands | 0 (0-0.1) | 0.1 (0-0.1) | 0 (0-0) | 0 (0-0.1) | -0.5 (-0.81--0.2) |
| Congenital musculoskeletal and limb anomalies | Oman | 9.2 (5.6-14.5) | 0.3 (0.2-0.5) | 5.3 (3.4-8) | 0.1 (0.1-0.2) | -1.87 (-2.42--1.31) |
| Congenital musculoskeletal and limb anomalies | Pakistan | 622.1 (293-1317.4) | 0.3 (0.2-0.7) | 689.1 (346.8-1524.7) | 0.2 (0.1-0.5) | -0.82 (-0.97--0.67) |
| Congenital musculoskeletal and limb anomalies | Palau | 0 (0-0) | 0.2 (0.1-0.3) | 0 (0-0) | 0.1 (0.1-0.2) | -1.55 (-1.71--1.4) |
| Congenital musculoskeletal and limb anomalies | Palestine | 16.4 (9.4-26.7) | 0.4 (0.3-0.7) | 9 (5.9-13) | 0.2 (0.1-0.2) | -2.98 (-3.23--2.73) |
| Congenital musculoskeletal and limb anomalies | People's Democratic Republic of Algeria | 322.8 (144.4-544.3) | 0.9 (0.4-1.5) | 166.5 (116.5-232.5) | 0.4 (0.3-0.5) | -2.69 (-2.81--2.57) |
| Congenital musculoskeletal and limb anomalies | People's Republic of China | 1402 (755.1-2324) | 0.1 (0.1-0.2) | 237.6 (151.5-318.9) | 0 (0-0) | -5.49 (-6.01--4.97) |
| Congenital musculoskeletal and limb anomalies | Plurinational State of Bolivia | 34.2 (12.1-61.1) | 0.3 (0.1-0.6) | 23.5 (15.6-33.6) | 0.2 (0.1-0.3) | -1.43 (-1.51--1.35) |
| Congenital musculoskeletal and limb anomalies | Portuguese Republic | 20.8 (17.4-24.5) | 0.3 (0.3-0.4) | 4.2 (3.4-5) | 0.1 (0.1-0.1) | -5.1 (-5.64--4.56) |
| Congenital musculoskeletal and limb anomalies | Principality of Andorra | 0 (0-0.1) | 0.1 (0.1-0.2) | 0 (0-0) | 0 (0-0) | -3.84 (-4.22--3.46) |
| Congenital musculoskeletal and limb anomalies | Puerto Rico | 3.4 (2.7-5.3) | 0.1 (0.1-0.2) | 0.8 (0.6-1.1) | 0.1 (0-0.1) | -1.3 (-2.31--0.28) |
| Congenital musculoskeletal and limb anomalies | Qatar | 3.5 (2.1-4.8) | 0.7 (0.4-0.9) | 4.2 (2.7-5.8) | 0.2 (0.1-0.3) | -3.88 (-4.15--3.6) |
| Congenital musculoskeletal and limb anomalies | Republic of Albania | 5.6 (3.3-8.5) | 0.1 (0.1-0.2) | 0.8 (0.4-1.4) | 0.1 (0-0.1) | -2.81 (-3.01--2.62) |
| Congenital musculoskeletal and limb anomalies | Republic of Armenia | 23 (14.5-34.3) | 0.6 (0.4-0.9) | 4.1 (2.8-6) | 0.2 (0.2-0.3) | -2.68 (-3.42--1.92) |
| Congenital musculoskeletal and limb anomalies | Republic of Austria | 12.2 (10.1-14) | 0.2 (0.2-0.3) | 4 (3.2-5.1) | 0.1 (0.1-0.1) | -3.14 (-3.55--2.74) |
| Congenital musculoskeletal and limb anomalies | Republic of Azerbaijan | 22.1 (13.7-34.8) | 0.3 (0.2-0.4) | 12.3 (7.1-22.6) | 0.2 (0.1-0.3) | -0.99 (-1.26--0.71) |
| Congenital musculoskeletal and limb anomalies | Republic of Belarus | 6 (3.3-11.9) | 0.1 (0-0.2) | 2.1 (1.5-3.4) | 0 (0-0.1) | -2.61 (-2.99--2.23) |
| Congenital musculoskeletal and limb anomalies | Republic of Bulgaria | 14.5 (11-17.9) | 0.3 (0.2-0.3) | 2.8 (2.1-3.7) | 0.1 (0.1-0.1) | -4.36 (-4.56--4.16) |
| Congenital musculoskeletal and limb anomalies | Republic of Chile | 28.2 (23-37.3) | 0.2 (0.2-0.3) | 14.1 (10.5-17.4) | 0.1 (0.1-0.2) | -0.45 (-0.98-0.09) |
| Congenital musculoskeletal and limb anomalies | Republic of Colombia | 57.7 (38.7-70.4) | 0.1 (0.1-0.2) | 23.7 (17.3-35.1) | 0.1 (0-0.1) | -1.9 (-2.51--1.28) |
| Congenital musculoskeletal and limb anomalies | Republic of Costa Rica | 10.8 (7.9-13.1) | 0.3 (0.2-0.4) | 9.3 (6.5-11.2) | 0.3 (0.2-0.3) | -0.37 (-0.59--0.14) |
| Congenital musculoskeletal and limb anomalies | Republic of Croatia | 5.2 (3.9-7.8) | 0.2 (0.1-0.3) | 1.8 (1.3-2.5) | 0.1 (0.1-0.1) | -1.99 (-2.2--1.77) |
| Congenital musculoskeletal and limb anomalies | Republic of Cuba | 16.4 (13.3-20.9) | 0.2 (0.1-0.2) | 6.2 (4.9-8.5) | 0.1 (0.1-0.1) | -2.29 (-2.67--1.91) |
| Congenital musculoskeletal and limb anomalies | Republic of Cyprus | 1 (0.5-1.8) | 0.1 (0.1-0.3) | 0.3 (0.2-0.5) | 0 (0-0.1) | -4.57 (-4.75--4.39) |
| Congenital musculoskeletal and limb anomalies | Republic of Ecuador | 7.7 (5.9-10.7) | 0.1 (0-0.1) | 20.7 (11.2-28.9) | 0.1 (0.1-0.2) | 3.59 (3.01-4.17) |
| Congenital musculoskeletal and limb anomalies | Republic of El Salvador | 14.8 (8.7-21.5) | 0.2 (0.1-0.3) | 6.5 (3-10.6) | 0.1 (0-0.2) | -1.44 (-1.65--1.23) |
| Congenital musculoskeletal and limb anomalies | Republic of Estonia | 3.3 (2.5-4.1) | 0.3 (0.2-0.3) | 0.5 (0.3-0.6) | 0.1 (0-0.1) | -5.93 (-6.32--5.55) |
| Congenital musculoskeletal and limb anomalies | Republic of Fiji | 0.8 (0.5-1.5) | 0.1 (0.1-0.2) | 0.6 (0.3-1.2) | 0.1 (0-0.1) | -1.21 (-1.42--1.01) |
| Congenital musculoskeletal and limb anomalies | Republic of Finland | 10.6 (8.1-13.1) | 0.3 (0.2-0.3) | 3.4 (2.7-4) | 0.1 (0.1-0.1) | -3.39 (-3.65--3.13) |
| Congenital musculoskeletal and limb anomalies | Republic of Guatemala | 15.8 (10.4-21.3) | 0.1 (0.1-0.1) | 17 (12-24.9) | 0.1 (0.1-0.2) | 0.77 (0.35-1.18) |
| Congenital musculoskeletal and limb anomalies | Republic of Guyana | 1.2 (0.9-1.6) | 0.1 (0.1-0.1) | 0.7 (0.5-1) | 0.1 (0.1-0.1) | 0.24 (0.04-0.44) |
| Congenital musculoskeletal and limb anomalies | Republic of Haiti | 64.8 (25.6-135.9) | 0.6 (0.2-1.2) | 70.1 (31.2-141.6) | 0.4 (0.2-0.9) | -0.65 (-0.86--0.43) |
| Congenital musculoskeletal and limb anomalies | Republic of Honduras | 18.3 (10.8-26.7) | 0.2 (0.1-0.3) | 13.4 (8-19.7) | 0.1 (0.1-0.2) | -1.94 (-2.04--1.84) |
| Congenital musculoskeletal and limb anomalies | Republic of Iceland | 0.5 (0.3-0.6) | 0.2 (0.1-0.3) | 0.2 (0.1-0.3) | 0.1 (0.1-0.1) | -3.41 (-3.74--3.08) |
| Congenital musculoskeletal and limb anomalies | Republic of Indonesia | 509.7 (223.4-907.8) | 0.2 (0.1-0.4) | 349.8 (194.4-507.5) | 0.2 (0.1-0.2) | -1.1 (-1.16--1.03) |
| Congenital musculoskeletal and limb anomalies | Republic of Iraq | 158.3 (92.1-267.1) | 0.5 (0.3-0.8) | 99.7 (66.5-159.3) | 0.2 (0.2-0.4) | -2.58 (-2.83--2.33) |
| Congenital musculoskeletal and limb anomalies | Republic of Italy | 68.5 (53.3-74.8) | 0.2 (0.2-0.2) | 16.3 (13-24) | 0 (0-0.1) | -4.48 (-4.8--4.15) |
| Congenital musculoskeletal and limb anomalies | Republic of Kazakhstan | 48.4 (32.4-72.9) | 0.3 (0.2-0.4) | 36.6 (25-53.8) | 0.2 (0.1-0.3) | -1.57 (-2.36--0.78) |
| Congenital musculoskeletal and limb anomalies | Republic of Kiribati | 0.3 (0.1-0.6) | 0.2 (0.1-0.5) | 0.2 (0.1-0.4) | 0.2 (0.1-0.3) | -1.41 (-1.51--1.31) |
| Congenital musculoskeletal and limb anomalies | Republic of Korea | 74.1 (49.5-113.5) | 0.2 (0.1-0.3) | 12.3 (8.2-17.6) | 0.1 (0-0.1) | -2.63 (-2.97--2.3) |
| Congenital musculoskeletal and limb anomalies | Republic of Latvia | 8.2 (6.3-10) | 0.4 (0.3-0.5) | 1 (0.7-1.2) | 0.1 (0.1-0.1) | -5.64 (-5.9--5.39) |
| Congenital musculoskeletal and limb anomalies | Republic of Lithuania | 9.4 (7.3-11.4) | 0.3 (0.2-0.4) | 1.5 (1.1-1.8) | 0.1 (0.1-0.1) | -4.4 (-4.68--4.11) |
| Congenital musculoskeletal and limb anomalies | Republic of Maldives | 1.7 (0.3-3.4) | 0.4 (0.1-0.8) | 0.5 (0.3-0.8) | 0.2 (0.1-0.3) | -2.52 (-2.67--2.37) |
| Congenital musculoskeletal and limb anomalies | Republic of Malta | 1 (0.8-1.2) | 0.3 (0.3-0.4) | 0.4 (0.3-0.6) | 0.2 (0.1-0.2) | -1.77 (-2.2--1.33) |
| Congenital musculoskeletal and limb anomalies | Republic of Moldova | 22.4 (15.4-28.8) | 0.6 (0.4-0.7) | 3.9 (2.7-5.2) | 0.2 (0.2-0.3) | -3.35 (-3.75--2.93) |
| Congenital musculoskeletal and limb anomalies | Republic of Nicaragua | 14.3 (8.5-22.9) | 0.2 (0.1-0.4) | 7.2 (4.4-10.5) | 0.1 (0.1-0.2) | -1.82 (-2.08--1.56) |
| Congenital musculoskeletal and limb anomalies | Republic of Panama | 6.6 (5.2-8.2) | 0.2 (0.2-0.3) | 7.8 (5.7-10) | 0.2 (0.2-0.3) | -0.34 (-0.56--0.11) |
| Congenital musculoskeletal and limb anomalies | Republic of Paraguay | 18.4 (12.5-27.9) | 0.3 (0.2-0.5) | 29.8 (14.8-45.5) | 0.5 (0.2-0.7) | 2.29 (1.89-2.7) |
| Congenital musculoskeletal and limb anomalies | Republic of Peru | 60.4 (29.5-101.7) | 0.2 (0.1-0.3) | 37.5 (21.8-56.7) | 0.1 (0.1-0.2) | -0.72 (-1.16--0.29) |
| Congenital musculoskeletal and limb anomalies | Republic of Poland | 73.9 (53.3-84.5) | 0.3 (0.2-0.3) | 11 (8.1-14.9) | 0.1 (0-0.1) | -5.47 (-6.34--4.6) |
| Congenital musculoskeletal and limb anomalies | Republic of Serbia | 26.9 (15.3-46.6) | 0.4 (0.2-0.7) | 3.7 (2.4-5.2) | 0.1 (0.1-0.1) | -4.72 (-5.22--4.22) |
| Congenital musculoskeletal and limb anomalies | Republic of Singapore | 4.6 (3.8-5.9) | 0.2 (0.2-0.2) | 1.5 (0.9-2.2) | 0 (0-0.1) | -3.2 (-3.67--2.72) |
| Congenital musculoskeletal and limb anomalies | Republic of Slovenia | 1.3 (1-2.1) | 0.1 (0.1-0.2) | 0.3 (0.2-0.4) | 0 (0-0) | -4.27 (-4.53--4.01) |
| Congenital musculoskeletal and limb anomalies | Republic of Suriname | 0.6 (0.4-0.9) | 0.1 (0.1-0.2) | 0.6 (0.4-0.8) | 0.1 (0.1-0.2) | -0.54 (-0.69--0.38) |
| Congenital musculoskeletal and limb anomalies | Republic of Tajikistan | 11 (6.6-19.1) | 0.1 (0.1-0.2) | 15.7 (9.1-26.6) | 0.1 (0.1-0.2) | 0.22 (0.09-0.35) |
| Congenital musculoskeletal and limb anomalies | Republic of the Marshall Islands | 0.1 (0-0.2) | 0.1 (0.1-0.3) | 0.1 (0-0.1) | 0.1 (0.1-0.2) | -0.91 (-1.02--0.8) |
| Congenital musculoskeletal and limb anomalies | Republic of the Philippines | 337.2 (196.7-543.2) | 0.4 (0.2-0.6) | 301.5 (221.6-394.4) | 0.3 (0.2-0.4) | -0.53 (-0.65--0.41) |
| Congenital musculoskeletal and limb anomalies | Republic of the Union of Myanmar | 215.3 (38.3-491.3) | 0.4 (0.1-0.9) | 151.4 (65.9-298.7) | 0.3 (0.1-0.6) | -1.13 (-1.34--0.93) |
| Congenital musculoskeletal and limb anomalies | Republic of Trinidad and Tobago | 1.9 (1.5-2.3) | 0.2 (0.1-0.2) | 1.1 (0.8-1.5) | 0.1 (0.1-0.2) | -0.68 (-0.94--0.41) |
| Congenital musculoskeletal and limb anomalies | Republic of Uzbekistan | 18.8 (12.6-28.6) | 0.1 (0-0.1) | 13.1 (7.3-24) | 0 (0-0.1) | -1.26 (-1.63--0.9) |
| Congenital musculoskeletal and limb anomalies | Republic of Vanuatu | 0.3 (0.1-0.6) | 0.1 (0.1-0.2) | 0.3 (0.2-0.6) | 0.1 (0.1-0.1) | -1.14 (-1.24--1.03) |
| Congenital musculoskeletal and limb anomalies | Romania | 31.6 (18.8-65.9) | 0.2 (0.1-0.4) | 7.5 (5.7-10.9) | 0.1 (0.1-0.1) | -2.94 (-3.11--2.78) |
| Congenital musculoskeletal and limb anomalies | Russian Federation | 116.1 (96-159.6) | 0.1 (0.1-0.1) | 42.7 (32.4-64) | 0 (0-0.1) | -3.14 (-3.4--2.89) |
| Congenital musculoskeletal and limb anomalies | Rwanda | 46.1 (20.7-90) | 0.3 (0.2-0.7) | 38.3 (19.4-75.3) | 0.2 (0.1-0.4) | -1.47 (-1.67--1.27) |
| Congenital musculoskeletal and limb anomalies | Saint Kitts and Nevis | 0.1 (0.1-0.1) | 0.2 (0.1-0.2) | 0 (0-0.1) | 0.1 (0.1-0.2) | -0.32 (-0.43--0.21) |
| Congenital musculoskeletal and limb anomalies | Saint Lucia | 0.3 (0.2-0.4) | 0.2 (0.1-0.2) | 0.1 (0.1-0.2) | 0.1 (0.1-0.2) | -0.58 (-0.74--0.42) |
| Congenital musculoskeletal and limb anomalies | Saint Vincent and the Grenadines | 0.2 (0.1-0.3) | 0.2 (0.1-0.2) | 0.1 (0.1-0.1) | 0.1 (0.1-0.1) | -1.62 (-1.81--1.43) |
| Congenital musculoskeletal and limb anomalies | San Marino | 0.1 (0-0.1) | 0.5 (0.3-0.7) | 0 (0-0) | 0.1 (0.1-0.2) | -3.88 (-4.09--3.67) |
| Congenital musculoskeletal and limb anomalies | Sao Tome and Principe | 0.6 (0.4-0.9) | 0.3 (0.2-0.5) | 0.4 (0.1-1) | 0.2 (0.1-0.4) | -1.53 (-1.77--1.3) |
| Congenital musculoskeletal and limb anomalies | Saudi Arabia | 33.6 (18.7-57.5) | 0.1 (0.1-0.2) | 16.9 (10.3-27) | 0 (0-0.1) | -3.29 (-3.44--3.13) |
| Congenital musculoskeletal and limb anomalies | Senegal | 49.5 (28.5-84.5) | 0.4 (0.2-0.6) | 61.6 (28.9-121.2) | 0.3 (0.1-0.6) | -0.26 (-0.46--0.06) |
| Congenital musculoskeletal and limb anomalies | Seychelles | 0.1 (0.1-0.2) | 0.2 (0.1-0.3) | 0.1 (0.1-0.2) | 0.2 (0.1-0.3) | 0.71 (0.51-0.91) |
| Congenital musculoskeletal and limb anomalies | Sierra Leone | 37.9 (16.9-69.6) | 0.5 (0.2-0.8) | 47.4 (28.9-80.3) | 0.4 (0.2-0.6) | -0.56 (-0.67--0.45) |
| Congenital musculoskeletal and limb anomalies | Slovak Republic | 17.3 (12.9-21.8) | 0.4 (0.3-0.5) | 5.1 (3.2-7.2) | 0.2 (0.1-0.2) | -3.01 (-3.22--2.79) |
| Congenital musculoskeletal and limb anomalies | Socialist Republic of Viet Nam | 83.5 (16.4-210) | 0.1 (0-0.2) | 35.2 (20.4-61.5) | 0 (0-0.1) | -1.96 (-2.23--1.69) |
| Congenital musculoskeletal and limb anomalies | Solomon Islands | 1 (0.5-1.7) | 0.2 (0.1-0.3) | 1 (0.6-1.6) | 0.1 (0.1-0.2) | -1.13 (-1.27--0.99) |
| Congenital musculoskeletal and limb anomalies | Somalia | 54.3 (22.1-110.8) | 0.3 (0.1-0.7) | 128.3 (59.7-246) | 0.3 (0.2-0.6) | 0.28 (0.04-0.53) |
| Congenital musculoskeletal and limb anomalies | South Africa | 163.3 (113.6-214.4) | 0.3 (0.2-0.4) | 177.4 (108-248.4) | 0.4 (0.2-0.5) | 0.63 (0.3-0.97) |
| Congenital musculoskeletal and limb anomalies | South Sudan | 40.3 (16.2-87) | 0.4 (0.2-0.8) | 76.5 (34-142.6) | 0.5 (0.2-0.8) | 1.19 (0.88-1.5) |
| Congenital musculoskeletal and limb anomalies | State of Israel | 17.2 (13.2-22.1) | 0.3 (0.3-0.4) | 9.1 (6.8-11.4) | 0.1 (0.1-0.1) | -3.28 (-3.57--2.98) |
| Congenital musculoskeletal and limb anomalies | State of Kuwait | 6 (4.9-7.9) | 0.3 (0.3-0.5) | 4.8 (3.8-6.3) | 0.2 (0.1-0.2) | -1.64 (-2.18--1.1) |
| Congenital musculoskeletal and limb anomalies | Sudan | 497 (118.1-973) | 1.4 (0.3-2.6) | 352.9 (201.9-551) | 0.7 (0.4-1) | -2.08 (-2.23--1.94) |
| Congenital musculoskeletal and limb anomalies | Swiss Confederation | 17.5 (13.8-21.9) | 0.4 (0.3-0.5) | 6.1 (4.7-7.5) | 0.1 (0.1-0.1) | -3.54 (-4--3.07) |
| Congenital musculoskeletal and limb anomalies | Syrian Arab Republic | 286.9 (133.9-568.8) | 1.4 (0.7-2.7) | 70.2 (44.5-97.5) | 0.6 (0.4-0.9) | -1.68 (-2.14--1.23) |
| Congenital musculoskeletal and limb anomalies | Taiwan (Province of China) | 21.6 (18.1-24.2) | 0.1 (0.1-0.1) | 5.6 (4.4-8.8) | 0 (0-0.1) | -4.81 (-5.73--3.89) |
| Congenital musculoskeletal and limb anomalies | Togo | 21.5 (13.1-34.9) | 0.3 (0.2-0.5) | 27.6 (15.8-49.4) | 0.3 (0.1-0.5) | -0.58 (-0.65--0.5) |
| Congenital musculoskeletal and limb anomalies | Tokelau | 0 (0-0) | 0.1 (0.1-0.2) | 0 (0-0) | 0.3 (0.1-0.6) | -0.72 (-2.12-0.7) |
| Congenital musculoskeletal and limb anomalies | Tunisia | 70.8 (30.6-123.4) | 0.7 (0.3-1.2) | 19.8 (12.7-28.6) | 0.2 (0.1-0.3) | -3.65 (-3.72--3.58) |
| Congenital musculoskeletal and limb anomalies | Turkey | 602.4 (223.6-1092.6) | 0.9 (0.3-1.6) | 102.3 (75.4-134.6) | 0.2 (0.1-0.2) | -4.89 (-5.06--4.72) |
| Congenital musculoskeletal and limb anomalies | Turkmenistan | 6.5 (4.1-10.5) | 0.1 (0.1-0.2) | 8.4 (5.4-13) | 0.2 (0.1-0.2) | 2.45 (1.8-3.1) |
| Congenital musculoskeletal and limb anomalies | Tuvalu | 0 (0-0.1) | 0.2 (0.1-0.5) | 0 (0-0) | 0.1 (0.1-0.2) | -2.69 (-2.9--2.49) |
| Congenital musculoskeletal and limb anomalies | Uganda | 102 (43.5-204.3) | 0.3 (0.1-0.5) | 175.5 (99.7-312.7) | 0.2 (0.1-0.4) | 0.2 (0.01-0.38) |
| Congenital musculoskeletal and limb anomalies | Ukraine | 45.6 (34.3-72.9) | 0.1 (0.1-0.2) | 12.2 (7.6-21.4) | 0.1 (0-0.1) | -2.83 (-3.05--2.62) |
| Congenital musculoskeletal and limb anomalies | United Arab Emirates | 15.3 (9.2-22.5) | 0.7 (0.4-1) | 11 (5.6-16.6) | 0.2 (0.1-0.3) | -3.12 (-3.51--2.73) |
| Congenital musculoskeletal and limb anomalies | United Kingdom of Great Britain and Northern Ireland | 70.8 (60.2-83.4) | 0.2 (0.1-0.2) | 36.2 (27.8-42) | 0.1 (0.1-0.1) | -1.6 (-1.84--1.36) |
| Congenital musculoskeletal and limb anomalies | United Mexican States | 260.7 (182.7-299.9) | 0.2 (0.2-0.3) | 137.6 (111.8-181.7) | 0.1 (0.1-0.2) | -1.92 (-2.29--1.55) |
| Congenital musculoskeletal and limb anomalies | United Republic of Tanzania | 189.2 (95.2-370.9) | 0.4 (0.2-0.7) | 271.4 (161.3-509.8) | 0.3 (0.2-0.6) | 0.08 (-0.13-0.28) |
| Congenital musculoskeletal and limb anomalies | United States of America | 290 (264.8-392.7) | 0.1 (0.1-0.2) | 294.6 (208.7-334.7) | 0.1 (0.1-0.2) | 0.33 (0.18-0.48) |
| Congenital musculoskeletal and limb anomalies | United States Virgin Islands | 0.1 (0.1-0.2) | 0.1 (0.1-0.2) | 0 (0-0.1) | 0.1 (0-0.1) | -0.84 (-1.06--0.62) |
| Congenital musculoskeletal and limb anomalies | Yemen | 313.7 (71.5-617.9) | 1.1 (0.3-2.2) | 292.2 (158.2-437.5) | 0.7 (0.4-1) | -1.81 (-1.89--1.73) |
| Congenital musculoskeletal and limb anomalies | Zambia | 52.4 (26.6-111.1) | 0.3 (0.2-0.7) | 76.1 (38.4-160.6) | 0.3 (0.1-0.6) | -0.21 (-0.39--0.04) |
| Congenital musculoskeletal and limb anomalies | Zimbabwe | 56.9 (25-95.3) | 0.3 (0.1-0.5) | 103.1 (47.9-167) | 0.5 (0.2-0.8) | 1.91 (1.66-2.17) |

Table S3 The congenital musculoskeletal and limb anomalies cases and age standardized prevalence from 1990 to 2021 in 204 countries and territories.

| Cause | Location | 1990 | | 2021 | | 1990-2021 |
| --- | --- | --- | --- | --- | --- | --- |
|  |  | Number of patients  No. ×10^3^ (95% UI) | ASPR per 100,000  No. (95% UI) | Number of patients  No. ×10^3^ (95% UI) | ASPR per 100,000  No. (95% UI) | EAPC  No. (95% CI) |
| Congenital musculoskeletal and limb anomalies | Afghanistan | 38179.2 (29684.1-49575.2) | 280.2 (221.8-358.5) | 114179.1 (89975.1-145937.7) | 277.1 (218.6-349) | -0.03 (-0.05--0.01) |
| Congenital musculoskeletal and limb anomalies | American Samoa | 117.3 (90.6-147.7) | 207.1 (161.8-261.1) | 105.5 (83-131.2) | 219.1 (172.4-273.6) | 0.21 (0.18-0.24) |
| Congenital musculoskeletal and limb anomalies | Angola | 32143.4 (25288-42175.1) | 221.9 (178.2-279.5) | 90545.7 (70238.8-113826.9) | 209.7 (166.1-260) | -0.2 (-0.22--0.18) |
| Congenital musculoskeletal and limb anomalies | Antigua and Barbuda | 172.3 (136.2-215.7) | 281.9 (222.9-352.2) | 235.9 (187.7-290.1) | 296.7 (233.9-366.6) | 0.14 (0.11-0.17) |
| Congenital musculoskeletal and limb anomalies | Arab Republic of Egypt | 179952.9 (140423.9-225726.6) | 266.6 (210-330.3) | 312053.9 (246356.2-384989.8) | 271 (214.8-334.4) | 0.11 (0.08-0.13) |
| Congenital musculoskeletal and limb anomalies | Argentine Republic | 129722.1 (104424.3-160802.2) | 385.5 (310.8-477.1) | 154836.3 (124527.2-191921.4) | 392.5 (312-483.5) | 0.06 (0.05-0.07) |
| Congenital musculoskeletal and limb anomalies | Australia | 43295.6 (34963.5-52567.4) | 278.4 (225.2-337.5) | 61981.7 (50216-76471) | 282.4 (228.5-346.2) | 0.03 (0.01-0.05) |
| Congenital musculoskeletal and limb anomalies | Bangladesh | 282498.1 (220612.1-355107.3) | 205.7 (162.9-257.8) | 309238.3 (244672.6-384758.3) | 191.8 (152.1-237.9) | -0.24 (-0.24--0.23) |
| Congenital musculoskeletal and limb anomalies | Barbados | 677.7 (540-839.1) | 281.8 (223.6-349.4) | 736.4 (592.6-907.1) | 296.3 (234-367.1) | 0.19 (0.17-0.2) |
| Congenital musculoskeletal and limb anomalies | Belize | 649.7 (497.6-818.9) | 285.7 (223.1-355.5) | 1319.6 (1036.3-1636.8) | 304.9 (239-378.4) | 0.18 (0.14-0.21) |
| Congenital musculoskeletal and limb anomalies | Benin | 14270.9 (11006.7-17896.3) | 209.4 (164.8-261.2) | 36113.9 (27674-46204.8) | 204.7 (160.1-256) | -0.08 (-0.11--0.06) |
| Congenital musculoskeletal and limb anomalies | Bermuda | 146.9 (115.3-179.7) | 264.9 (205.6-326.3) | 148.2 (117.2-183) | 282.2 (222.4-351.8) | 0.23 (0.22-0.24) |
| Congenital musculoskeletal and limb anomalies | Bhutan | 1557 (1221-1943.3) | 203.8 (160.4-253.8) | 1392.3 (1102.4-1725.7) | 192.2 (152.8-240.5) | -0.21 (-0.22--0.2) |
| Congenital musculoskeletal and limb anomalies | Bolivarian Republic of Venezuela | 92539.1 (69620.1-119277.7) | 410.4 (314.1-521.9) | 105466.7 (82035.7-133805.5) | 430.2 (333.7-550.3) | 0.04 (0-0.09) |
| Congenital musculoskeletal and limb anomalies | Bosnia and Herzegovina | 10655.2 (8567.8-13248.5) | 246.5 (198.5-304.8) | 7367.4 (5837.6-8983) | 264 (211.2-324.6) | 0.21 (0.16-0.25) |
| Congenital musculoskeletal and limb anomalies | Botswana | 3474.3 (2670.7-4476.6) | 210.5 (164.2-264.5) | 5081.1 (3917.5-6290.4) | 208.4 (160.7-258.4) | -0.09 (-0.13--0.06) |
| Congenital musculoskeletal and limb anomalies | Brunei Darussalam | 1366.6 (1043.2-1737.1) | 449.3 (344.7-564.9) | 1669.8 (1304.6-2067.5) | 447.9 (348.3-560.6) | 0.01 (0-0.03) |
| Congenital musculoskeletal and limb anomalies | Burkina Faso | 28212.5 (22004.6-36101.9) | 209.2 (166.1-265.1) | 65549.8 (50976.5-83251.8) | 211 (168.8-265.3) | 0.03 (-0.01-0.07) |
| Congenital musculoskeletal and limb anomalies | Burundi | 17423.2 (13660.1-22351.3) | 222.2 (176.7-278.6) | 38080.3 (29787.2-48839.2) | 220.4 (174.8-280.6) | -0.06 (-0.08--0.04) |
| Congenital musculoskeletal and limb anomalies | Cabo Verde | 775.7 (606.4-995.1) | 188.9 (148.4-236.9) | 964.1 (750.1-1198.9) | 177.8 (138.2-221.7) | -0.24 (-0.27--0.21) |
| Congenital musculoskeletal and limb anomalies | Cameroon | 29198.3 (22857.9-37613.3) | 205.9 (163.4-258.9) | 79927.4 (62738.9-101911.3) | 204 (161.1-258.3) | -0.01 (-0.04-0.01) |
| Congenital musculoskeletal and limb anomalies | Canada | 62280.5 (48992-76178.3) | 244 (194-298.3) | 77695.4 (61701.2-96057.9) | 235.8 (189.5-291.5) | -0.13 (-0.15--0.11) |
| Congenital musculoskeletal and limb anomalies | Central African Republic | 9059.6 (6996.5-11740.3) | 235.1 (184.9-295.2) | 17335.8 (13412.4-22450.5) | 242.7 (190.3-309.8) | 0.11 (0.1-0.12) |
| Congenital musculoskeletal and limb anomalies | Chad | 18953 (14738.9-24461.3) | 215.1 (172.1-268.4) | 57804.9 (45203.2-75343.1) | 218.3 (174.6-274.5) | 0.04 (0.01-0.06) |
| Congenital musculoskeletal and limb anomalies | Commonwealth of Dominica | 231.9 (181.1-289.7) | 295.4 (231.2-366.9) | 183.9 (145.3-227) | 317 (246.6-391.1) | 0.23 (0.21-0.24) |
| Congenital musculoskeletal and limb anomalies | Commonwealth of the Bahamas | 791.4 (618.5-976.9) | 294.1 (231.1-363.6) | 1090.1 (853.1-1346) | 315.3 (246.1-394.3) | 0.18 (0.16-0.2) |
| Congenital musculoskeletal and limb anomalies | Comoros | 1262.1 (996.9-1601.6) | 210 (167.2-263.4) | 1628.2 (1294.4-2030.9) | 206.7 (164-257.7) | -0.11 (-0.15--0.07) |
| Congenital musculoskeletal and limb anomalies | Congo | 6729.8 (5315.5-8573.9) | 218.1 (173.8-272.9) | 12311.4 (9703.9-15429.4) | 207.2 (163.4-258.5) | -0.18 (-0.2--0.16) |
| Congenital musculoskeletal and limb anomalies | Cook Islands | 39.8 (30.9-49.4) | 197.2 (153.5-244) | 32.9 (25.8-41) | 203.1 (160.3-252.7) | 0.16 (0.12-0.2) |
| Congenital musculoskeletal and limb anomalies | C么te d'Ivoire | 34314.5 (26664.2-44157) | 207.8 (166.1-263) | 69849.9 (54360.2-88405.6) | 203.2 (160.6-255.3) | -0.07 (-0.09--0.05) |
| Congenital musculoskeletal and limb anomalies | Czech Republic | 26037 (20853.8-31706.5) | 274.8 (220-335.8) | 27624.6 (21778.3-33741.9) | 298.7 (235.7-364.6) | 0.24 (0.12-0.36) |
| Congenital musculoskeletal and limb anomalies | Democratic People's Republic of Korea | 45876.8 (36084.8-57222.9) | 211.1 (166.1-262.1) | 53699.5 (42086.3-66263.9) | 241.5 (188.9-299.4) | 0.46 (0.41-0.51) |
| Congenital musculoskeletal and limb anomalies | Democratic Republic of the Congo | 113068.5 (87732.2-144045) | 213.4 (168.6-268.4) | 235376.6 (183119.2-295650.2) | 211.5 (167.2-265.2) | -0.01 (-0.05-0.02) |
| Congenital musculoskeletal and limb anomalies | Democratic Republic of Timor-Leste | 2605 (2033.7-3323.9) | 246.6 (194.9-310.5) | 3865.4 (3011.2-4852.6) | 238.3 (187.5-296.3) | -0.17 (-0.21--0.13) |
| Congenital musculoskeletal and limb anomalies | Democratic Socialist Republic of Sri Lanka | 41565.9 (32505.6-51246.8) | 232 (182-285.3) | 44763.3 (35380.8-55544.2) | 216.3 (169.7-267.3) | -0.25 (-0.31--0.2) |
| Congenital musculoskeletal and limb anomalies | Djibouti | 1062.9 (837-1362.1) | 205.9 (162.6-258.9) | 2763.9 (2132.3-3461.8) | 203.4 (157.1-254.9) | -0.08 (-0.1--0.05) |
| Congenital musculoskeletal and limb anomalies | Dominican Republic | 24420.1 (18770.1-30644) | 292.9 (227.8-361.9) | 32902.3 (26057.6-41217) | 304.4 (240.3-381.7) | 0.15 (0.12-0.19) |
| Congenital musculoskeletal and limb anomalies | Eastern Republic of Uruguay | 11192.5 (8937.2-13772.8) | 374.4 (298-461.3) | 10774.8 (8570.7-13240.7) | 382.4 (304.8-474.2) | 0.03 (0.02-0.05) |
| Congenital musculoskeletal and limb anomalies | Equatorial Guinea | 1330.5 (1040.8-1701.3) | 224.4 (175.7-285.1) | 3196.9 (2493.2-4040.7) | 187.7 (148.1-235.8) | -0.72 (-0.8--0.65) |
| Congenital musculoskeletal and limb anomalies | Eritrea | 10548.8 (8310.3-13519.5) | 224.7 (179-285.8) | 17274.1 (13517-21739.7) | 219 (172.1-274.4) | -0.07 (-0.08--0.06) |
| Congenital musculoskeletal and limb anomalies | Eswatini | 2242.4 (1771.7-2819.3) | 211.2 (168.3-263.9) | 2769 (2138.8-3508.7) | 213.6 (165.4-267.8) | 0.05 (0.02-0.08) |
| Congenital musculoskeletal and limb anomalies | Ethiopia | 166500.3 (129927.7-214540) | 233.7 (185.3-293.6) | 278867.4 (221129.5-351271.4) | 210.9 (169-261.5) | -0.35 (-0.37--0.33) |
| Congenital musculoskeletal and limb anomalies | Federal Republic of Germany | 147781.5 (122243.7-178738.3) | 240.5 (198.3-291.9) | 142619.5 (114377.5-173646.1) | 234.5 (185.6-291.6) | -0.07 (-0.17-0.02) |
| Congenital musculoskeletal and limb anomalies | Federated States of Micronesia | 296.6 (228.6-374.8) | 231.8 (181.7-290.5) | 242.9 (189.8-306.5) | 233.1 (182.6-293.5) | 0.02 (-0.01-0.04) |
| Congenital musculoskeletal and limb anomalies | Federative Republic of Brazil | 357016.9 (295289-426037.4) | 223.1 (184.8-264.6) | 498985.3 (400500.1-609939) | 248.7 (200.1-303.2) | 0.38 (0.33-0.43) |
| Congenital musculoskeletal and limb anomalies | French Republic | 90411.4 (76996.2-107251.4) | 184.4 (157.6-217.1) | 98641.7 (80668.2-119607.2) | 193.8 (159.6-235.5) | 0.23 (0.13-0.32) |
| Congenital musculoskeletal and limb anomalies | Gabon | 2456.5 (1903.6-3113.3) | 205.8 (161.3-255.6) | 3857.7 (3004.1-4884.5) | 195.6 (153.5-246) | -0.16 (-0.17--0.14) |
| Congenital musculoskeletal and limb anomalies | Gambia | 2672.7 (2069.8-3429.4) | 204.6 (159.2-257) | 5919.9 (4571.9-7599.2) | 204.5 (160.6-254.8) | 0 (-0.02-0.01) |
| Congenital musculoskeletal and limb anomalies | Georgia | 15439 (12278-19056.1) | 292.3 (233.3-362.1) | 10288.8 (8888.9-11956.6) | 324.6 (282.4-372.5) | 0.55 (0.43-0.67) |
| Congenital musculoskeletal and limb anomalies | Ghana | 39067.2 (30376-50096.7) | 202.7 (159.6-255.3) | 78386.1 (61044.8-98115) | 199.4 (156.1-248.4) | 0 (-0.02-0.01) |
| Congenital musculoskeletal and limb anomalies | Global | 15570720.8 (12578431.7-19071446.4) | 273.5 (221.5-333.3) | 18549408.3 (15159832.6-22636856.8) | 249.1 (203.5-304.1) | -0.38 (-0.43--0.32) |
| Congenital musculoskeletal and limb anomalies | Grand Duchy of Luxembourg | 1073.7 (867.5-1328.2) | 351.9 (278.4-440.2) | 1684.5 (1353.6-2068.7) | 343 (271.8-422.8) | -0.26 (-0.34--0.19) |
| Congenital musculoskeletal and limb anomalies | Greenland | 144.8 (115.4-180.9) | 258.6 (206.5-323.1) | 130.8 (103.8-161.2) | 259.4 (206.9-316.5) | 0 (0-0.01) |
| Congenital musculoskeletal and limb anomalies | Grenada | 290.1 (223.7-364.8) | 297.1 (232.1-370.8) | 285.7 (227.2-355.1) | 306.9 (243.7-383.5) | 0.12 (0.11-0.13) |
| Congenital musculoskeletal and limb anomalies | Guam | 278.1 (216.1-345.6) | 192.4 (150.6-238.9) | 303 (238.4-375.1) | 202.7 (158.6-250.8) | 0.15 (0.14-0.17) |
| Congenital musculoskeletal and limb anomalies | Guinea | 17984.8 (13704.4-23156.6) | 214.6 (166.1-270.1) | 37677.6 (29435.2-48383.3) | 213.2 (170.9-268.7) | 0.01 (-0.01-0.03) |
| Congenital musculoskeletal and limb anomalies | Guinea-Bissau | 3257.3 (2512.2-4247.7) | 229.2 (179.2-291.6) | 6055.4 (4749.1-7833) | 225 (179.2-283.5) | -0.05 (-0.06--0.04) |
| Congenital musculoskeletal and limb anomalies | Hashemite Kingdom of Jordan | 11518.3 (8892.1-14430.8) | 254.7 (198.1-318.1) | 32404.2 (25605-40401.3) | 260.7 (205.8-325.5) | -0.03 (-0.06-0.01) |
| Congenital musculoskeletal and limb anomalies | Hellenic Republic | 38128.4 (30564.6-46631.9) | 441.4 (349.9-540) | 33131.6 (27888.3-39535.9) | 474.2 (394.3-569.8) | 0.32 (0.26-0.37) |
| Congenital musculoskeletal and limb anomalies | Hungary | 17100.7 (13545.7-21380.1) | 182.1 (144-226.1) | 15968.1 (12666.7-19589.1) | 196 (155-241.2) | 0.21 (0.15-0.27) |
| Congenital musculoskeletal and limb anomalies | Independent State of Papua New Guinea | 11006.5 (8634.3-14075.8) | 214.3 (170.9-272.8) | 28654.3 (22725.3-36232.9) | 228 (183.3-286) | 0.23 (0.22-0.25) |
| Congenital musculoskeletal and limb anomalies | Independent State of Samoa | 427.1 (330.2-535) | 211.6 (165.6-263) | 521.6 (406.9-658.8) | 216.7 (170-273.1) | 0.12 (0.08-0.15) |
| Congenital musculoskeletal and limb anomalies | India | 2089250.2 (1668789-2611191.5) | 214.1 (171.5-265.6) | 2771851.2 (2209735.5-3438770.9) | 204.8 (163.8-253.8) | -0.17 (-0.19--0.15) |
| Congenital musculoskeletal and limb anomalies | Ireland | 8011.9 (6727-9539.3) | 232 (196.7-274.7) | 10493.5 (8540-12661.1) | 260.5 (212-317.2) | 0.62 (0.53-0.72) |
| Congenital musculoskeletal and limb anomalies | Islamic Republic of Iran | 188513.4 (147879.5-234132.4) | 275.6 (218.4-338.5) | 233048 (184354.6-283579.5) | 294.1 (232.3-361.2) | 0.2 (0.09-0.31) |
| Congenital musculoskeletal and limb anomalies | Jamaica | 7085.6 (5602.7-8962.7) | 279.3 (220.5-351.3) | 7538.7 (6055-9194.7) | 297.3 (237.4-364) | 0.22 (0.2-0.23) |
| Congenital musculoskeletal and limb anomalies | Japan | 507939 (406436.7-628202.9) | 523.2 (408.2-656.5) | 345599.2 (281971.2-414980.8) | 488.3 (385.2-603.2) | -0.33 (-0.43--0.24) |
| Congenital musculoskeletal and limb anomalies | Kenya | 62421.3 (49133-78425) | 209.3 (167.2-259.1) | 118050.2 (93959-145589.2) | 212.3 (169.7-260.8) | 0.11 (0.07-0.14) |
| Congenital musculoskeletal and limb anomalies | Kingdom of Bahrain | 1419.6 (1109.8-1760.4) | 260.8 (205.5-323.8) | 3748.8 (2924.6-4627.7) | 262 (203.4-321.7) | -0.04 (-0.06--0.02) |
| Congenital musculoskeletal and limb anomalies | Kingdom of Belgium | 14874 (12465.2-17830.5) | 182.7 (152.6-218) | 17449 (14399.2-21177.2) | 203.2 (164-247.8) | 0.55 (0.46-0.63) |
| Congenital musculoskeletal and limb anomalies | Kingdom of Cambodia | 35356.2 (27208.7-44308.5) | 255.2 (201.2-319.4) | 41233 (32216-52137.4) | 235.7 (184.4-297.8) | -0.31 (-0.33--0.28) |
| Congenital musculoskeletal and limb anomalies | Kingdom of Denmark | 7272.8 (5981.3-8676.2) | 176.5 (145.3-213) | 7999.4 (6398.1-9790.7) | 180.5 (141.7-224.8) | 0.08 (0.02-0.13) |
| Congenital musculoskeletal and limb anomalies | Kingdom of Norway | 9179.1 (7420.7-11192.6) | 259.9 (206.8-318.5) | 10954.3 (8874.1-13373.5) | 261.3 (207.8-321.8) | -0.04 (-0.11-0.04) |
| Congenital musculoskeletal and limb anomalies | Kingdom of Spain | 65164.4 (54072.3-78253.8) | 196.9 (165.3-232.6) | 85687.2 (70835-103479.1) | 285 (228.5-354) | 1.54 (1.3-1.78) |
| Congenital musculoskeletal and limb anomalies | Kingdom of Sweden | 26174.6 (21242.6-31797.6) | 371.8 (297.8-456.3) | 30704.8 (25048.9-37019.7) | 379.7 (306.6-465.3) | 0.1 (0.06-0.13) |
| Congenital musculoskeletal and limb anomalies | Kingdom of Thailand | 128010.1 (100790.7-157529.3) | 221.8 (175.3-272.5) | 119187.4 (94596.9-146877.4) | 215.7 (170.9-265.6) | -0.04 (-0.09-0.01) |
| Congenital musculoskeletal and limb anomalies | Kingdom of the Netherlands | 26558.4 (22293.9-31927.1) | 211.3 (179.1-252.2) | 28509.3 (23166.6-35228.7) | 215.8 (174.5-268.9) | 0.23 (0.09-0.38) |
| Congenital musculoskeletal and limb anomalies | Kingdom of Tonga | 235 (183-294.1) | 202.7 (158-251.4) | 247.5 (195.3-311.5) | 210.8 (167.9-265.8) | 0.15 (0.14-0.17) |
| Congenital musculoskeletal and limb anomalies | Kyrgyz Republic | 15638.2 (12257.1-19353.6) | 309.3 (243-382.6) | 22594.1 (17718.9-27980.8) | 312.3 (245.5-387.5) | 0.07 (0.05-0.09) |
| Congenital musculoskeletal and limb anomalies | Lao People's Democratic Republic | 14669 (11630.3-18836.9) | 261.1 (210.3-330.3) | 19261.2 (15225-24139.3) | 244.3 (193.4-306.6) | -0.26 (-0.28--0.25) |
| Congenital musculoskeletal and limb anomalies | Lebanon | 8412.2 (6513.1-10465.7) | 255.5 (199.8-317.3) | 13358.4 (10650.8-16538.7) | 257.5 (206-320.3) | 0 (-0.03-0.03) |
| Congenital musculoskeletal and limb anomalies | Lesotho | 4117.3 (3188.7-5238.2) | 215.3 (168.5-271.3) | 4571.1 (3613.2-5831.7) | 224.7 (177.4-285.3) | 0.19 (0.16-0.22) |
| Congenital musculoskeletal and limb anomalies | Liberia | 7029 (5497.2-9064.3) | 210.5 (168.3-265) | 12968.9 (10225.5-16387.3) | 200 (158-249.7) | -0.2 (-0.24--0.17) |
| Congenital musculoskeletal and limb anomalies | Libya | 12681.6 (9931.4-16054) | 251.2 (198.4-314) | 16970.2 (13444.9-20946.2) | 270 (214.3-331.3) | 0.17 (0.12-0.22) |
| Congenital musculoskeletal and limb anomalies | Madagascar | 33876 (26447.7-42901.5) | 212.6 (168.3-264.9) | 73094.6 (57215.5-92265.5) | 211.9 (168.3-264.7) | 0 (-0.01-0.01) |
| Congenital musculoskeletal and limb anomalies | Malawi | 30554.2 (24170.1-39095.5) | 218.1 (175.3-272.3) | 50471.9 (40086.8-65210.9) | 212.7 (169.9-271.3) | -0.1 (-0.11--0.09) |
| Congenital musculoskeletal and limb anomalies | Malaysia | 47017.1 (36715.1-59280.8) | 238.1 (187.4-296.8) | 67585.5 (53605.4-83824.6) | 222.4 (175.5-275.2) | -0.27 (-0.33--0.22) |
| Congenital musculoskeletal and limb anomalies | Mali | 26327.7 (20457.5-33719.2) | 211.4 (165.2-264.8) | 71217 (54761-91500) | 209.9 (165.3-263.7) | -0.03 (-0.05-0) |
| Congenital musculoskeletal and limb anomalies | Mauritania | 5526.7 (4298.5-7073.2) | 208.5 (163.9-263.5) | 9849.2 (7704.9-12492.4) | 190.4 (148.9-239.2) | -0.26 (-0.27--0.24) |
| Congenital musculoskeletal and limb anomalies | Mauritius | 2506.5 (1981.4-3114.7) | 223.7 (176.7-278.2) | 2473.8 (1951.7-3023.8) | 221.4 (174.8-271.3) | 0 (-0.06-0.05) |
| Congenital musculoskeletal and limb anomalies | Monaco | 50.7 (40.9-61.6) | 245.1 (193.7-305) | 65 (52.1-80) | 250.3 (197.4-311.8) | 0.07 (0.05-0.09) |
| Congenital musculoskeletal and limb anomalies | Mongolia | 9009.1 (7110.8-11344) | 336.7 (270-422.5) | 11548.5 (9184.8-14283) | 327 (260.2-405.3) | -0.1 (-0.11--0.09) |
| Congenital musculoskeletal and limb anomalies | Montenegro | 1346.4 (1065.5-1665.4) | 221 (174.6-273.1) | 1395.4 (1121.4-1720.4) | 252.4 (203-310.1) | 0.39 (0.33-0.45) |
| Congenital musculoskeletal and limb anomalies | Morocco | 79849.1 (62663.1-101836.9) | 267.7 (212.9-339.7) | 99600 (79583.1-122770.9) | 276.6 (220.5-342.2) | 0.12 (0.11-0.13) |
| Congenital musculoskeletal and limb anomalies | Mozambique | 40589 (31641.3-52884.6) | 221.2 (176-282.5) | 90500.2 (70261.7-115588.5) | 219.8 (173.8-276.1) | 0.03 (0.01-0.06) |
| Congenital musculoskeletal and limb anomalies | Namibia | 3612.1 (2836-4578.7) | 208.8 (164-261.1) | 5230 (4124.7-6480.8) | 199.6 (158.8-246.4) | -0.14 (-0.16--0.12) |
| Congenital musculoskeletal and limb anomalies | Nauru | 27.7 (21.4-35) | 218.6 (170.9-271.9) | 28.5 (22.4-35.9) | 228.2 (181.3-286) | 0.14 (0.1-0.18) |
| Congenital musculoskeletal and limb anomalies | Nepal | 50845.9 (39838.8-64990.4) | 208.2 (165.1-263.5) | 62333.8 (49128.1-78201) | 196.1 (154.5-245.8) | -0.17 (-0.19--0.15) |
| Congenital musculoskeletal and limb anomalies | New Zealand | 11397.5 (9150.6-13960.4) | 353.8 (283.7-433.3) | 14385 (12047.2-17168.1) | 325.7 (273.1-387.5) | -0.3 (-0.36--0.24) |
| Congenital musculoskeletal and limb anomalies | Niger | 26620.2 (20358.4-34919.6) | 216.7 (171.4-275.1) | 80504.8 (61780.2-103908.6) | 215.2 (170.2-271.6) | -0.07 (-0.11--0.03) |
| Congenital musculoskeletal and limb anomalies | Nigeria | 249214.5 (199055.6-317415.7) | 211.1 (171.2-263.5) | 609650.7 (484960.9-772927.3) | 209.6 (169.6-260.7) | -0.04 (-0.06--0.03) |
| Congenital musculoskeletal and limb anomalies | Niue | 5 (4-6.3) | 209.9 (165.3-259.4) | 3.3 (2.6-4) | 212.6 (165.5-261.8) | 0.04 (0.01-0.07) |
| Congenital musculoskeletal and limb anomalies | North Macedonia | 4631.4 (3677.5-5700) | 238.4 (189-294.2) | 4771.3 (3764.3-5829.2) | 255 (201.8-311.3) | 0.22 (0.2-0.25) |
| Congenital musculoskeletal and limb anomalies | Northern Mariana Islands | 90.6 (71.4-113.1) | 193.8 (152.4-240.3) | 94.5 (75.3-117.7) | 207.1 (163.6-257.4) | 0.23 (0.2-0.26) |
| Congenital musculoskeletal and limb anomalies | Oman | 6330.3 (4974.1-8020.2) | 262.8 (209.1-328.7) | 12027 (9419.8-15046.4) | 260.4 (203.3-324.2) | 0.01 (-0.01-0.04) |
| Congenital musculoskeletal and limb anomalies | Pakistan | 290723.1 (230271-367234.3) | 210.8 (168.5-263.3) | 559382.6 (444993.5-691744) | 212.3 (169.5-260.7) | 0.01 (-0.02-0.04) |
| Congenital musculoskeletal and limb anomalies | Palau | 31.8 (25-39.6) | 203.4 (160.4-253.1) | 33 (26-40.7) | 211 (167-259.1) | 0.14 (0.1-0.18) |
| Congenital musculoskeletal and limb anomalies | Palestine | 4613.7 (3607.6-5865.3) | 178 (140.9-222.6) | 10131.8 (7829.3-12644) | 183.1 (141.8-227.5) | 0.13 (0.1-0.16) |
| Congenital musculoskeletal and limb anomalies | People's Democratic Republic of Algeria | 78042.9 (61866.9-99571.2) | 257.7 (206.3-322.1) | 118690.1 (93594.8-147600.1) | 264.2 (208.5-328.5) | 0.06 (0.05-0.08) |
| Congenital musculoskeletal and limb anomalies | People's Republic of China | 3740925.7 (3014593.7-4627167.6) | 316.4 (254.5-391.8) | 3112683.7 (2565594.2-3820090.2) | 266.1 (219.5-325.4) | -0.87 (-1.12--0.62) |
| Congenital musculoskeletal and limb anomalies | Plurinational State of Bolivia | 22231 (16821.7-28420.1) | 270 (208.1-339.3) | 33838.5 (26280.9-42596.5) | 281.2 (218.6-353.3) | 0.13 (0.11-0.14) |
| Congenital musculoskeletal and limb anomalies | Portuguese Republic | 23348.4 (18677.2-28890.6) | 275.9 (220.6-344.2) | 19971.7 (16692.5-23862.1) | 279.7 (230.5-338) | 0.12 (0.06-0.18) |
| Congenital musculoskeletal and limb anomalies | Principality of Andorra | 104.8 (84-129.7) | 240.2 (190.8-300.7) | 140.1 (112.3-172.3) | 240.7 (191.8-298.3) | 0.02 (0-0.05) |
| Congenital musculoskeletal and limb anomalies | Puerto Rico | 9731.7 (7660.1-12229.9) | 272.3 (214.5-342.6) | 7636.6 (6073.3-9420.6) | 282.2 (220.7-351.6) | 0.08 (0.06-0.11) |
| Congenital musculoskeletal and limb anomalies | Qatar | 2047.1 (1620.9-2499.3) | 440.6 (350.4-537.2) | 11841.7 (9244.5-14572.5) | 416.6 (326.4-507.9) | -0.42 (-0.56--0.27) |
| Congenital musculoskeletal and limb anomalies | Republic of Albania | 8474 (6699.3-10567.7) | 238.1 (188.7-297.7) | 5694.1 (4570.5-7137.9) | 247.8 (198.5-309.1) | 0.13 (0.11-0.16) |
| Congenital musculoskeletal and limb anomalies | Republic of Armenia | 10482.1 (8238.9-12857.9) | 297.3 (233.8-364.1) | 8095.1 (6401-10045) | 300 (236.3-372) | 0.06 (0.03-0.09) |
| Congenital musculoskeletal and limb anomalies | Republic of Austria | 12052.1 (9776.8-14718.4) | 193.2 (156.6-240.7) | 13306.4 (10636.7-16382.2) | 205.4 (161.6-256.9) | 0.21 (0.08-0.33) |
| Congenital musculoskeletal and limb anomalies | Republic of Azerbaijan | 24283.7 (19222.9-30228.5) | 307.6 (244.6-381.3) | 29935.3 (23841.6-36936.8) | 310 (245.7-385.4) | 0.08 (0.05-0.1) |
| Congenital musculoskeletal and limb anomalies | Republic of Belarus | 26742.6 (21178.7-32933.5) | 276.4 (219-340.2) | 22640.9 (17836.6-27955.9) | 283.7 (222.8-348.8) | 0.11 (0.08-0.14) |
| Congenital musculoskeletal and limb anomalies | Republic of Bulgaria | 18060.5 (14255-22075.2) | 233.5 (184.2-288.7) | 14288 (11475.3-17438.6) | 261.4 (208.1-322.3) | 0.38 (0.33-0.43) |
| Congenital musculoskeletal and limb anomalies | Republic of Chile | 53181.9 (42792.8-64979.4) | 384.8 (310.2-469.1) | 60880.2 (49753.1-72940.4) | 388.3 (324.8-464.2) | 0.08 (0-0.16) |
| Congenital musculoskeletal and limb anomalies | Republic of Colombia | 153189.2 (117086.1-194486.9) | 404.3 (313.3-506.5) | 163920.1 (127991.7-205287.2) | 384.1 (294.4-492.2) | -0.14 (-0.18--0.1) |
| Congenital musculoskeletal and limb anomalies | Republic of Costa Rica | 12501.6 (9335.8-16133.6) | 357.8 (275.2-453.4) | 15638.2 (12327-19334.8) | 380.5 (294-477.6) | 0.17 (0.14-0.21) |
| Congenital musculoskeletal and limb anomalies | Republic of Croatia | 9372.7 (7710.7-11499.9) | 211.1 (175.5-257.2) | 7853.4 (6319.3-9702) | 218.6 (173.3-272.7) | 0.05 (-0.01-0.1) |
| Congenital musculoskeletal and limb anomalies | Republic of Cuba | 27756.8 (21823.1-34682.7) | 264.9 (207-332.1) | 26721 (21318.9-32689.9) | 277.9 (221.5-340.3) | 0.14 (0.12-0.16) |
| Congenital musculoskeletal and limb anomalies | Republic of Cyprus | 1528.9 (1213.1-1913.2) | 210.6 (167.1-264.6) | 2059.8 (1647.3-2532) | 199.1 (157-251) | -0.23 (-0.38--0.07) |
| Congenital musculoskeletal and limb anomalies | Republic of Ecuador | 30454.8 (25213.8-36081.4) | 254.3 (213.5-300.1) | 52432.1 (45795.7-59261.7) | 295.3 (258.3-333.1) | 0.65 (0.44-0.86) |
| Congenital musculoskeletal and limb anomalies | Republic of El Salvador | 28631.2 (21372.3-37755.7) | 425.1 (322.5-546.4) | 26072.9 (19960-33503.1) | 409.8 (313.6-527) | -0.08 (-0.12--0.05) |
| Congenital musculoskeletal and limb anomalies | Republic of Estonia | 4033.1 (3196.1-4922.1) | 277.8 (219.6-340.2) | 3115 (2487-3860.3) | 278.9 (225.3-342) | 0.02 (-0.02-0.06) |
| Congenital musculoskeletal and limb anomalies | Republic of Fiji | 1798.9 (1411.5-2248.2) | 212.6 (168-265.4) | 2082.4 (1635.9-2606.1) | 223 (175.3-279.4) | 0.19 (0.17-0.2) |
| Congenital musculoskeletal and limb anomalies | Republic of Finland | 8426.1 (6865.9-10304.4) | 204.5 (166.9-253.5) | 8745.3 (7010-10697.7) | 220.3 (173.6-272) | 0.17 (0.13-0.22) |
| Congenital musculoskeletal and limb anomalies | Republic of Guatemala | 53672.3 (40586-69285.5) | 438.6 (340.6-552) | 72489.8 (55486.9-92653) | 436.4 (333.6-555.5) | -0.03 (-0.06-0) |
| Congenital musculoskeletal and limb anomalies | Republic of Guyana | 3012.8 (2299.5-3800.3) | 321.4 (248.8-402.4) | 2577.9 (2041.1-3257) | 337.4 (266.8-426.3) | 0.18 (0.17-0.19) |
| Congenital musculoskeletal and limb anomalies | Republic of Haiti | 25490.6 (20001.8-32112.6) | 306.6 (241.6-381.4) | 45297.6 (35553.8-56994.1) | 314.8 (248.1-393.9) | 0.09 (0.09-0.1) |
| Congenital musculoskeletal and limb anomalies | Republic of Honduras | 28407.3 (20834.9-36877.5) | 429.3 (326-547.9) | 49530.1 (37617.6-62778.3) | 449.4 (343.3-565.8) | 0.13 (0.09-0.16) |
| Congenital musculoskeletal and limb anomalies | Republic of Iceland | 666.5 (531.4-823.6) | 278.4 (222.1-345) | 806.2 (648.4-993.3) | 277.7 (220.3-347.1) | 0.02 (-0.06-0.1) |
| Congenital musculoskeletal and limb anomalies | Republic of Indonesia | 509692.2 (404374.1-630083.7) | 246.4 (196.7-306.1) | 639780.7 (514381.6-783909) | 243.3 (195.3-300.2) | -0.06 (-0.08--0.03) |
| Congenital musculoskeletal and limb anomalies | Republic of Iraq | 61094.7 (47029.7-78309.7) | 261.6 (203.3-331.3) | 115417.2 (91714.5-144003.4) | 268 (213.6-333.7) | 0.02 (0-0.04) |
| Congenital musculoskeletal and limb anomalies | Republic of Italy | 214988.8 (174321.5-258393.9) | 466.9 (376.3-571.3) | 180938.9 (153286.1-211055.4) | 467.9 (391.4-557.9) | -0.02 (-0.17-0.12) |
| Congenital musculoskeletal and limb anomalies | Republic of Kazakhstan | 53003.5 (41776.4-66295) | 308.8 (244.1-386.8) | 58901.9 (46495.8-72494.6) | 309 (243.6-380.5) | -0.03 (-0.08-0.02) |
| Congenital musculoskeletal and limb anomalies | Republic of Kiribati | 221.8 (174.3-280.7) | 236.5 (187.8-298.1) | 323.1 (254.3-402.1) | 241.1 (190.2-300.1) | 0.09 (0.07-0.12) |
| Congenital musculoskeletal and limb anomalies | Republic of Korea | 191609.6 (149838.2-242187.5) | 460.5 (360.1-584.1) | 127018.8 (100954.6-155757.2) | 425.7 (326.3-537.1) | -0.34 (-0.38--0.31) |
| Congenital musculoskeletal and limb anomalies | Republic of Latvia | 6779.9 (5457.9-8333.7) | 278.4 (223.4-341.5) | 4470.3 (3541.3-5513) | 289.9 (230.3-357.9) | 0.22 (0.18-0.27) |
| Congenital musculoskeletal and limb anomalies | Republic of Lithuania | 9474.4 (7578.8-11674.3) | 275.7 (219.6-340.5) | 6581.2 (5268.4-8043.6) | 294 (236.9-361.5) | 0.29 (0.22-0.35) |
| Congenital musculoskeletal and limb anomalies | Republic of Maldives | 666.5 (515.8-838.5) | 232.2 (184-288.2) | 950.7 (758.4-1183.1) | 201.7 (160.4-251.1) | -0.49 (-0.58--0.4) |
| Congenital musculoskeletal and limb anomalies | Republic of Malta | 731.3 (597.7-889.4) | 218.7 (179-266.2) | 709.7 (572.3-875.9) | 221.3 (174.8-273.6) | 0.05 (0-0.1) |
| Congenital musculoskeletal and limb anomalies | Republic of Moldova | 12369.6 (9729.9-15136.8) | 282.4 (221.9-347.4) | 8616.7 (6784-10600.7) | 293.2 (230.8-359.4) | 0.2 (0.16-0.23) |
| Congenital musculoskeletal and limb anomalies | Republic of Nicaragua | 20392.4 (15244.5-26447.6) | 387.3 (299.1-488.6) | 26739.1 (20499.8-33880.4) | 393.2 (302.2-496.8) | -0.02 (-0.04-0.01) |
| Congenital musculoskeletal and limb anomalies | Republic of Panama | 9881.9 (7582.2-12638.2) | 367.2 (287.3-464.7) | 15629.3 (12092.6-19609.3) | 380.2 (292.5-479.2) | 0.09 (0.06-0.12) |
| Congenital musculoskeletal and limb anomalies | Republic of Paraguay | 10192.1 (7943.3-12706.1) | 215.4 (170.6-264.8) | 15720.9 (12490.2-19279.3) | 221.3 (176.1-270.9) | 0.07 (0.06-0.08) |
| Congenital musculoskeletal and limb anomalies | Republic of Peru | 62301 (48111.7-79559.8) | 245.2 (191.3-310.1) | 84719 (65420-107793.3) | 240.9 (185.7-307.1) | -0.09 (-0.1--0.07) |
| Congenital musculoskeletal and limb anomalies | Republic of Poland | 67789.4 (53726.8-83323.4) | 188.1 (148.8-232.1) | 66027.1 (52582.5-80436.6) | 202 (159.6-245.1) | 0.22 (0.19-0.25) |
| Congenital musculoskeletal and limb anomalies | Republic of Serbia | 29278.9 (23184.3-36098.9) | 324.8 (256.9-401.8) | 26461.6 (21046.1-32827.2) | 350.4 (279.4-428.8) | 0 (-0.12-0.12) |
| Congenital musculoskeletal and limb anomalies | Republic of Singapore | 12104.9 (9508.8-15083.5) | 462.4 (357.1-582.9) | 16255.9 (13165.8-20273.2) | 439.7 (343.7-558.7) | -0.19 (-0.22--0.16) |
| Congenital musculoskeletal and limb anomalies | Republic of Slovenia | 5090.7 (4047.9-6237.7) | 278.5 (222.5-336.9) | 4967.6 (3951.7-6094.1) | 274.9 (219.1-337.1) | 0.04 (-0.03-0.12) |
| Congenital musculoskeletal and limb anomalies | Republic of Suriname | 1265.3 (982.1-1590.9) | 301.2 (236-372.7) | 1717.1 (1362.2-2144.7) | 318.5 (251.7-401) | 0.17 (0.16-0.18) |
| Congenital musculoskeletal and limb anomalies | Republic of Tajikistan | 20909.2 (16413.9-26420.9) | 310 (243.3-389.7) | 37297.4 (29744.4-46361.5) | 327.5 (261.2-406.9) | 0.25 (0.22-0.28) |
| Congenital musculoskeletal and limb anomalies | Republic of the Marshall Islands | 133.7 (104-170.4) | 228.8 (181.1-286.5) | 139.7 (111-175.4) | 238.2 (189.9-298.1) | 0.12 (0.1-0.13) |
| Congenital musculoskeletal and limb anomalies | Republic of the Philippines | 173996.4 (137919.2-214876.9) | 234.2 (186.3-289.4) | 275287.9 (220097.7-339978.1) | 238.3 (190.8-294.1) | 0.07 (0.06-0.09) |
| Congenital musculoskeletal and limb anomalies | Republic of the Union of Myanmar | 117191.1 (93633.6-146924.8) | 252.1 (202.1-316.8) | 129889.4 (103544.4-161704.2) | 233.6 (186-291.5) | -0.3 (-0.32--0.28) |
| Congenital musculoskeletal and limb anomalies | Republic of Trinidad and Tobago | 3801.3 (3042-4715.5) | 297.4 (238.6-367.6) | 3676.8 (2914.6-4594.1) | 307.3 (242.7-387.2) | 0.05 (0.03-0.08) |
| Congenital musculoskeletal and limb anomalies | Republic of Uzbekistan | 76606.4 (60406-95530.2) | 306.8 (242.4-384) | 114613.3 (90136.8-143058.1) | 323.3 (253.7-402.6) | 0.17 (0.15-0.19) |
| Congenital musculoskeletal and limb anomalies | Republic of Vanuatu | 439.3 (338.2-555.1) | 222.3 (173.3-276) | 853.8 (668.3-1065.2) | 236.4 (185.6-293.4) | 0.2 (0.19-0.22) |
| Congenital musculoskeletal and limb anomalies | Romania | 51740.4 (40808.8-64996.6) | 238 (188.4-296.8) | 39883.5 (31717.7-49015.5) | 251.6 (200.7-308.9) | 0.19 (0.15-0.22) |
| Congenital musculoskeletal and limb anomalies | Russian Federation | 539971.7 (428707.5-655820) | 386.6 (307.1-471.2) | 488767.4 (387984.9-594467.3) | 389.7 (310.2-472.1) | 0.02 (0-0.03) |
| Congenital musculoskeletal and limb anomalies | Rwanda | 22396.5 (17504.2-29075.2) | 224.8 (179.3-286.3) | 31790.3 (25108.9-39850.6) | 207 (163.9-257.6) | -0.35 (-0.39--0.31) |
| Congenital musculoskeletal and limb anomalies | Saint Kitts and Nevis | 136.4 (108.5-170.4) | 308 (246.4-381.9) | 152.4 (123.1-189.3) | 306.4 (243.3-381.7) | -0.02 (-0.05-0.02) |
| Congenital musculoskeletal and limb anomalies | Saint Lucia | 457.3 (353.2-570.6) | 298.5 (233.4-368.9) | 457.1 (361-564.2) | 306.1 (238.3-380.6) | 0.1 (0.07-0.13) |
| Congenital musculoskeletal and limb anomalies | Saint Vincent and the Grenadines | 360.9 (283.4-448) | 295.8 (234.2-363.8) | 319.1 (251.3-395.7) | 315.2 (246.8-392.1) | 0.21 (0.2-0.22) |
| Congenital musculoskeletal and limb anomalies | San Marino | 44.3 (35.6-54.5) | 231.7 (182-289.1) | 54.9 (44.1-68.1) | 239.6 (188.3-299.5) | 0.15 (0.13-0.16) |
| Congenital musculoskeletal and limb anomalies | Sao Tome and Principe | 295.9 (229.4-374.3) | 197.5 (154.1-246.3) | 440.8 (346.9-549.7) | 188.9 (148.7-234) | -0.15 (-0.16--0.14) |
| Congenital musculoskeletal and limb anomalies | Saudi Arabia | 51615.8 (39854-65856.9) | 269.6 (209.9-338.9) | 91512.1 (71396.4-113288.3) | 262.2 (206.7-323.4) | -0.12 (-0.13--0.11) |
| Congenital musculoskeletal and limb anomalies | Senegal | 21341.8 (16500.7-27667.2) | 206.8 (161.4-261.1) | 38059.4 (30003.7-47149.7) | 202.5 (160.4-249.2) | -0.02 (-0.05-0.01) |
| Congenital musculoskeletal and limb anomalies | Seychelles | 179.6 (143.4-219.2) | 234.9 (187.5-287.5) | 222.5 (176.4-275) | 226.5 (178.4-280.1) | -0.13 (-0.19--0.07) |
| Congenital musculoskeletal and limb anomalies | Sierra Leone | 11814.6 (9323.5-15043.4) | 208.7 (167.2-263.6) | 22758.6 (17901.1-29207.8) | 206.9 (164-261.9) | 0 (-0.02-0.01) |
| Congenital musculoskeletal and limb anomalies | Slovak Republic | 11698.8 (9180-14457.7) | 232.8 (182.3-287.8) | 11875.5 (9310.3-14601.3) | 252.4 (199-308.4) | 0.29 (0.25-0.32) |
| Congenital musculoskeletal and limb anomalies | Socialist Republic of Viet Nam | 176632.4 (138520.9-221524.9) | 228 (180-284.8) | 196770.8 (154734.8-243927.6) | 206.1 (162.6-256.9) | -0.36 (-0.41--0.32) |
| Congenital musculoskeletal and limb anomalies | Solomon Islands | 1074.9 (836.4-1359.9) | 235 (187.2-291.7) | 1975 (1540-2485.8) | 242.8 (191.2-303.9) | 0.13 (0.11-0.15) |
| Congenital musculoskeletal and limb anomalies | Somalia | 26150.9 (20555.1-34048.8) | 227.1 (180.9-289.3) | 73653.1 (56571.3-95220) | 234.3 (184.4-297.6) | 0.1 (0.09-0.11) |
| Congenital musculoskeletal and limb anomalies | South Africa | 83978.9 (65947.5-104656.4) | 203.3 (161.2-251.9) | 112107.4 (88877-139741) | 201.9 (160.1-252.2) | -0.05 (-0.08--0.02) |
| Congenital musculoskeletal and limb anomalies | South Sudan | 16594.8 (13110.9-21354.5) | 211.2 (169-266.1) | 26938.7 (21019.5-34553.7) | 211.3 (167.2-270.8) | -0.02 (-0.03-0) |
| Congenital musculoskeletal and limb anomalies | State of Israel | 12900.9 (10288.5-16043.8) | 251.4 (201.1-311.7) | 22849.4 (17902.5-28865) | 246.4 (192.9-311.4) | -0.09 (-0.1--0.07) |
| Congenital musculoskeletal and limb anomalies | State of Kuwait | 4246.1 (3365.9-5267.6) | 235.4 (187-291.4) | 10667.7 (8289.3-13279) | 246 (194.5-305.4) | 0.15 (0.14-0.16) |
| Congenital musculoskeletal and limb anomalies | Sudan | 74826.3 (58451.1-96177.8) | 274 (217.4-344.3) | 137907.6 (108875.1-173614.1) | 276.8 (219.3-346.6) | 0.01 (0-0.02) |
| Congenital musculoskeletal and limb anomalies | Swiss Confederation | 11458.9 (9546.6-13819.8) | 207.5 (172.5-249.6) | 14386.3 (11633.4-17612.8) | 217.9 (173.3-268.3) | 0.18 (0.01-0.36) |
| Congenital musculoskeletal and limb anomalies | Syrian Arab Republic | 41954.1 (32263.3-53273) | 261.8 (203.1-327.6) | 35518.9 (28010.9-44178.5) | 263.1 (207.1-326.7) | 0 (-0.05-0.05) |
| Congenital musculoskeletal and limb anomalies | Taiwan (Province of China) | 38547 (30654.7-47695.1) | 193.3 (153.1-240.8) | 42070.9 (33225.7-52960.8) | 213.3 (169.6-266.2) | 0.34 (0.3-0.37) |
| Congenital musculoskeletal and limb anomalies | Togo | 10202.7 (7861.8-13126.1) | 207 (162.9-258.1) | 20344 (16062.4-25627.5) | 206.3 (162.9-258.4) | -0.03 (-0.04--0.01) |
| Congenital musculoskeletal and limb anomalies | Tokelau | 3.9 (3-4.8) | 221.8 (174.9-274.5) | 2.8 (2.2-3.5) | 216.8 (171.9-267.7) | -0.05 (-0.07--0.03) |
| Congenital musculoskeletal and limb anomalies | Tunisia | 22675.5 (17656.3-27991.9) | 245.4 (193.1-302.7) | 28301.5 (22307.6-35126.3) | 254.9 (200.7-314.9) | 0.11 (0.1-0.12) |
| Congenital musculoskeletal and limb anomalies | Turkey | 153441.7 (121456.4-193370.8) | 243 (193.1-302.7) | 186754.2 (148051.1-232282.6) | 242.5 (192.6-300.4) | 0.15 (0.04-0.26) |
| Congenital musculoskeletal and limb anomalies | Turkmenistan | 13821.5 (10772.5-17549.7) | 311.5 (243.9-387.8) | 17002.4 (13519.6-21143.7) | 321.6 (255.7-399.7) | 0.15 (0.12-0.18) |
| Congenital musculoskeletal and limb anomalies | Tuvalu | 25.6 (19.9-31.9) | 224.1 (176.2-279.1) | 28.1 (22.1-35.2) | 220.7 (173.7-276.5) | -0.01 (-0.04-0.02) |
| Congenital musculoskeletal and limb anomalies | Uganda | 52250.4 (40464.3-67386) | 209.7 (166.3-263.7) | 116387.5 (91437.5-148900.9) | 205.9 (162.8-257.3) | -0.1 (-0.13--0.08) |
| Congenital musculoskeletal and limb anomalies | Ukraine | 109551.1 (86810-134501.1) | 229.8 (180.7-283.3) | 84726 (66548.1-104440.1) | 248.7 (195.6-305.3) | 0.33 (0.3-0.35) |
| Congenital musculoskeletal and limb anomalies | United Arab Emirates | 5512.2 (4352-6849.2) | 274.1 (216.5-339) | 23613.2 (18531.5-29380.4) | 284.1 (223.5-351.1) | 0.08 (0.06-0.1) |
| Congenital musculoskeletal and limb anomalies | United Kingdom of Great Britain and Northern Ireland | 121728.8 (98721-148858.8) | 256.5 (206.3-315.7) | 133461.7 (107700.8-162711) | 256 (205.7-316) | -0.01 (-0.02-0) |
| Congenital musculoskeletal and limb anomalies | United Mexican States | 595773.1 (459836.9-758739) | 571.2 (449.1-714.1) | 587042.5 (471408.2-714405) | 492.9 (394.4-605.9) | -0.42 (-0.57--0.27) |
| Congenital musculoskeletal and limb anomalies | United Republic of Tanzania | 71424.2 (55630.7-90344.3) | 205.5 (162.3-255.6) | 145384.7 (114536.1-186505.9) | 203.7 (161.9-259) | -0.03 (-0.04--0.03) |
| Congenital musculoskeletal and limb anomalies | United States of America | 595960.2 (476144.7-732399.5) | 250.9 (201.1-308.4) | 592614.4 (484977.6-720774.8) | 207 (170.7-248.4) | -0.19 (-0.32--0.06) |
| Congenital musculoskeletal and limb anomalies | United States Virgin Islands | 310.9 (241.8-389.8) | 285.6 (222.5-357.6) | 211.5 (167.2-264.5) | 306 (240.3-383.6) | 0.21 (0.19-0.22) |
| Congenital musculoskeletal and limb anomalies | Yemen | 54978.4 (42139.5-69674.2) | 276.2 (218.3-348.1) | 109677 (87145.4-136323.3) | 276.1 (219.9-345.9) | -0.02 (-0.03-0) |
| Congenital musculoskeletal and limb anomalies | Zambia | 24531.3 (19267-31168.7) | 218.6 (173.2-273.4) | 50502.8 (39223.3-64251.3) | 209.1 (165.5-263.1) | -0.18 (-0.22--0.13) |
| Congenital musculoskeletal and limb anomalies | Zimbabwe | 26502.9 (20654.9-33690.9) | 201.8 (159-254.3) | 41229.7 (32156.1-53267) | 219.9 (173.7-279.8) | 0.41 (0.35-0.48) |

Table S4. Predicted trends of congenital musculoskeletal and limb anomalies incidence, deaths and prevalence in the next decade (2021-2031).

| Year | Number of incidence | Number of deaths | Number of patients | Type |
| --- | --- | --- | --- | --- |
| 1990 | 2521672.1 | 18901.9 | 15570720.8 | Actual |
| 1991 | 2500889.4 | 18697.9 | 15843186.4 | Actual |
| 1992 | 2485214.3 | 18572.1 | 16086308.9 | Actual |
| 1993 | 2476298.0 | 18449.4 | 16300514.3 | Actual |
| 1994 | 2467259.1 | 18299.4 | 16475937.5 | Actual |
| 1995 | 2459058.7 | 18110.8 | 16608673.7 | Actual |
| 1996 | 2453359.0 | 17835.0 | 16711293.0 | Actual |
| 1997 | 2449040.2 | 17590.3 | 16799560.1 | Actual |
| 1998 | 2448297.2 | 17363.0 | 16881530.0 | Actual |
| 1999 | 2451731.7 | 17187.8 | 16967722.2 | Actual |
| 2000 | 2458775.4 | 17002.8 | 17071796.7 | Actual |
| 2001 | 2466238.2 | 16838.2 | 17207253.4 | Actual |
| 2002 | 2470922.5 | 16752.1 | 17370549.8 | Actual |
| 2003 | 2477070.3 | 16761.9 | 17546421.5 | Actual |
| 2004 | 2489012.6 | 16796.4 | 17719342.3 | Actual |
| 2005 | 2510074.9 | 16793.4 | 17876663.4 | Actual |
| 2006 | 2538984.5 | 16757.7 | 17978464.0 | Actual |
| 2007 | 2567822.6 | 16731.5 | 18015006.5 | Actual |
| 2008 | 2589338.0 | 16701.7 | 18020668.7 | Actual |
| 2009 | 2598744.0 | 16622.3 | 18025306.6 | Actual |
| 2010 | 2585013.2 | 16478.5 | 18049398.8 | Actual |
| 2011 | 2589780.3 | 16379.2 | 18104806.9 | Actual |
| 2012 | 2623303.5 | 16305.4 | 18197109.9 | Actual |
| 2013 | 2639705.8 | 16277.0 | 18315081.6 | Actual |
| 2014 | 2652149.8 | 16154.4 | 18425190.8 | Actual |
| 2015 | 2678446.6 | 16042.2 | 18523558.5 | Actual |
| 2016 | 2686951.8 | 15800.3 | 18592173.6 | Actual |
| 2017 | 2654663.9 | 15474.6 | 18614378.4 | Actual |
| 2018 | 2605041.2 | 15078.4 | 18606114.4 | Actual |
| 2019 | 2544310.3 | 14742.8 | 18608283.8 | Actual |
| 2020 | 2471139.7 | 14086.9 | 18533535.7 | Actual |
| 2021 | 2437890.1 | 13599.8 | 18549408.3 | Actual |
| 2022 | 2449310.8 | 13150.7 | 18598734.8 | Forecast |
| 2023 | 2458596.5 | 12734.3 | 18660411.4 | Forecast |
| 2024 | 2458596.5 | 12345.7 | 18726647.2 | Forecast |
| 2025 | 2458596.5 | 11981.2 | 18794566.1 | Forecast |
| 2026 | 2458596.5 | 11637.3 | 18863106.3 | Forecast |
| 2027 | 2458596.5 | 11310.9 | 18931875.9 | Forecast |
| 2028 | 2458596.5 | 10999.8 | 19000730.2 | Forecast |
| 2029 | 2458596.5 | 10701.6 | 19069615.8 | Forecast |
| 2030 | 2458596.5 | 10414.5 | 19138512.8 | Forecast |
| 2031 | 2458596.5 | 10137.0 | 19207414.2 | Forecast |

Table S5. Changes in incidence cases, deaths number, and number of patients according to population-level determinants from 1990 to 2021.

| Category | Congenital musculoskeletal and limb anomalies | | | | | | | | | | | |
| --- | --- | --- | --- | --- | --- | --- | --- | --- | --- | --- | --- | --- |
|  | Incidence cases | | | | Deaths | | | | Number of patients | | | |
|  | Overall difference | Aging | Population | Epidemiological change | Overall difference | Aging | Population | Epidemiological change | Overall difference | Aging | Population | Epidemiological change |
| Location |  |  |  |  |  |  |  |  |  |  |  |  |
| Global | 20841779656 | 933641062.8 (4.48%) | 20898845758 (100.27%) | -990707165 (-4.75%) | -1E+07 | -23710692.606 (235.66%) | 59582396.488 (-592.2%) | -45932925.206 (456.53%) | 54830601969 | -11150820485.419 (-20.34%) | 41127141397.018 (75.01%) | 24854281057.327 (45.33%) |
| Low SDI | 5533111852 | -172973765.3 (-3.13%) | 5723819861 (103.45%) | -17734243.69 (-0.32%) | 16234989 | -800914.917 (-4.93%) | 9769446.433 (60.18%) | 7266457.68 (44.76%) | 12803296366 | -471764265.288 (-3.68%) | 9818551885.454 (76.69%) | 3456508745.385 (27%) |
| Low-middle SDI | 5368342907 | -866313695.2 (-16.14%) | 7078565241 (131.86%) | -843908638.8 (-15.72%) | 17896086 | -4610150.211 (-25.76%) | 11501219.566 (64.27%) | 11005016.725 (61.49%) | 14554594865 | -1405399654.28 (-9.66%) | 10185181151.561 (69.98%) | 5774813367.335 (39.68%) |
| High SDI | 1126787930 | -310479399.6 (-27.55%) | 1859051829 (164.99%) | -421784499.8 (-37.43%) | 2619072 | 30839.107 (1.18%) | 2898815.647 (110.68%) | -310582.889 (-11.86%) | 6423042027 | 470986982.418 (7.33%) | 2848446120.022 (44.35%) | 3103608924.85 (48.32%) |
| High-middle SDI | 1420991398 | -155430198.3 (-10.94%) | 1911337656 (134.51%) | -334916060.4 (-23.57%) | 102267.1 | 1967502.529 (1923.89%) | 2233527.325 (2184.01%) | -4098762.748 (-4007.9%) | 8625276501 | 1795478445.639 (20.82%) | 3115206223.626 (36.12%) | 3714591831.347 (43.07%) |
| Middle SDI | 5820243482 | 230959229.9 (3.97%) | 5926812821 (101.83%) | -337528568.7 (-5.8%) | -7383578 | -10855688.703 (147.02%) | 16461291.331 (-222.94%) | -12989181.049 (175.92%) | 14121230918 | -5350085416.059 (-37.89%) | 11507316556.049 (81.49%) | 7963999777.888 (56.4%) |
| Sex |  |  |  |  |  |  |  |  |  |  |  |  |
| Both | 42502764389 | 3727289811.213 (8.77%) | 41401769674.947 (97.41%) | -2626295096.791 (-6.18%) | 65340359 | -8344984.648 (-12.77%) | 25959851.66 (39.73%) | 47725491.929 (73.04%) | 1.75E+10 | -729612711.709 (-4.16%) | 9018239312.889 (51.4%) | 9256458419.313 (52.76%) |
| Male | 10464356455 | 992980453.951 (9.49%) | 10318135986.903 (98.6%) | -846759985.376 (-8.09%) | 20023164 | -2534381.846 (-12.66%) | 7666636.015 (38.29%) | 14890909.798 (74.37%) | 4.22E+09 | -203068393.199 (-4.81%) | 2170338787.378 (51.4%) | 2254831854.82 (53.41%) |
| Female | 10786145655 | 871277527.239 (8.08%) | 10382248602.932 (96.26%) | -467380475.31 (-4.33%) | 12847357 | -1631092.885 (-12.7%) | 5334057.377 (41.52%) | 9144392.708 (71.18%) | 4.54E+09 | -163119786.732 (-3.59%) | 2336491960.243 (51.42%) | 2370617633.371 (52.17%) |

%: contribute to the total changes

Table S6. The trends in slope index of inequality and concentration index of congenital musculoskeletal and limb anomalies crude incidence, mortality and prevalence rates from 1990 to 2021.

| year | congenital musculoskeletal and limb anomalies | | |
| --- | --- | --- | --- |
|  | Incidence | Mortality | Prevalence |
|  | SII | SII | SII |
| 1990 | -84.02 (-92.6, -75.45) | -0.66 (-0.74, -0.58) | -47.44 (-74.91, -19.97) |
| 1991 | -83.61 (-92.07, -75.16) | -0.66 (-0.73, -0.58) | -46.64 (-74.58, -18.69) |
| 1992 | -83.45 (-92.05, -74.85) | -0.65 (-0.72, -0.58) | -46.75 (-74.36, -19.14) |
| 1993 | -84.08 (-92.55, -75.61) | -0.65 (-0.71, -0.58) | -47.92 (-75.49, -20.36) |
| 1994 | -84.09 (-92.63, -75.56) | -0.63 (-0.7, -0.57) | -48 (-75.51, -20.48) |
| 1995 | -85.55 (-93.53, -77.56) | -0.63 (-0.7, -0.57) | -51.28 (-78.95, -23.62) |
| 1996 | -86.81 (-94.49, -79.12) | -0.63 (-0.69, -0.56) | -55.22 (-82.83, -27.61) |
| 1997 | -86.94 (-94.46, -79.41) | -0.61 (-0.68, -0.55) | -57.54 (-85.09, -29.99) |
| 1998 | -87.5 (-94.65, -80.34) | -0.61 (-0.67, -0.55) | -60.96 (-88.3, -33.62) |
| 1999 | -87.37 (-94.37, -80.38) | -0.61 (-0.67, -0.55) | -61.17 (-88.46, -33.88) |
| 2000 | -86.78 (-93.68, -79.88) | -0.6 (-0.66, -0.53) | -62.87 (-90, -35.74) |
| 2001 | -86.49 (-93.15, -79.82) | -0.58 (-0.64, -0.52) | -64.57 (-90.95, -38.18) |
| 2002 | -86.06 (-92.81, -79.31) | -0.59 (-0.65, -0.53) | -63.94 (-90.27, -37.62) |
| 2003 | -84.26 (-91.23, -77.3) | -0.58 (-0.65, -0.52) | -64.17 (-90.02, -38.31) |
| 2004 | -83.5 (-90.45, -76.56) | -0.59 (-0.66, -0.52) | -63.66 (-89.37, -37.96) |
| 2005 | -82.36 (-89.25, -75.47) | -0.59 (-0.66, -0.52) | -63.03 (-88.95, -37.11) |
| 2006 | -80.99 (-87.78, -74.21) | -0.58 (-0.65, -0.51) | -63.38 (-88.67, -38.09) |
| 2007 | -78.77 (-85.67, -71.88) | -0.58 (-0.65, -0.51) | -61.49 (-86.53, -36.46) |
| 2008 | -77.02 (-83.99, -70.05) | -0.57 (-0.65, -0.5) | -59.01 (-83.61, -34.4) |
| 2009 | -75.83 (-82.87, -68.78) | -0.57 (-0.64, -0.5) | -56.73 (-81.46, -31.99) |
| 2010 | -74.78 (-81.71, -67.85) | -0.55 (-0.62, -0.48) | -58.82 (-83.4, -34.23) |
| 2011 | -74.26 (-81.22, -67.3) | -0.55 (-0.62, -0.48) | -58.76 (-83.49, -34.04) |
| 2012 | -72.7 (-79.78, -65.62) | -0.53 (-0.61, -0.46) | -59.24 (-83.73, -34.75) |
| 2013 | -71.12 (-77.98, -64.26) | -0.52 (-0.59, -0.45) | -58.07 (-82.49, -33.64) |
| 2014 | -69.97 (-77.05, -62.89) | -0.51 (-0.58, -0.44) | -57.79 (-82.32, -33.26) |
| 2015 | -68.66 (-75.7, -61.62) | -0.49 (-0.56, -0.42) | -57.1 (-81.59, -32.6) |
| 2016 | -67.34 (-74.13, -60.54) | -0.48 (-0.55, -0.41) | -56.17 (-80.35, -31.99) |
| 2017 | -66.22 (-73, -59.44) | -0.46 (-0.53, -0.39) | -56.17 (-80.22, -32.12) |
| 2018 | -65.06 (-71.99, -58.13) | -0.44 (-0.5, -0.37) | -55 (-79.21, -30.79) |
| 2019 | -64.33 (-71.26, -57.4) | -0.42 (-0.49, -0.36) | -55.75 (-80.08, -31.43) |
| 2020 | -62.61 (-69.5, -55.71) | -0.41 (-0.47, -0.35) | -56.66 (-81.18, -32.14) |
| 2021 | -62.17 (-69.01, -55.32) | -0.39 (-0.45, -0.34) | -55.56 (-79.86, -31.25) |
|  | CI | CI | CI |
| 1990 | 0.28 (0.18, 0.38) | 0.34 (0.23, 0.45) | 0.05 (0.05, 0.05) |
| 2021 | 0.35 (0.25, 0.45) | 0.42 (0.31, 0.53) | 0.05 (0.05, 0.05) |


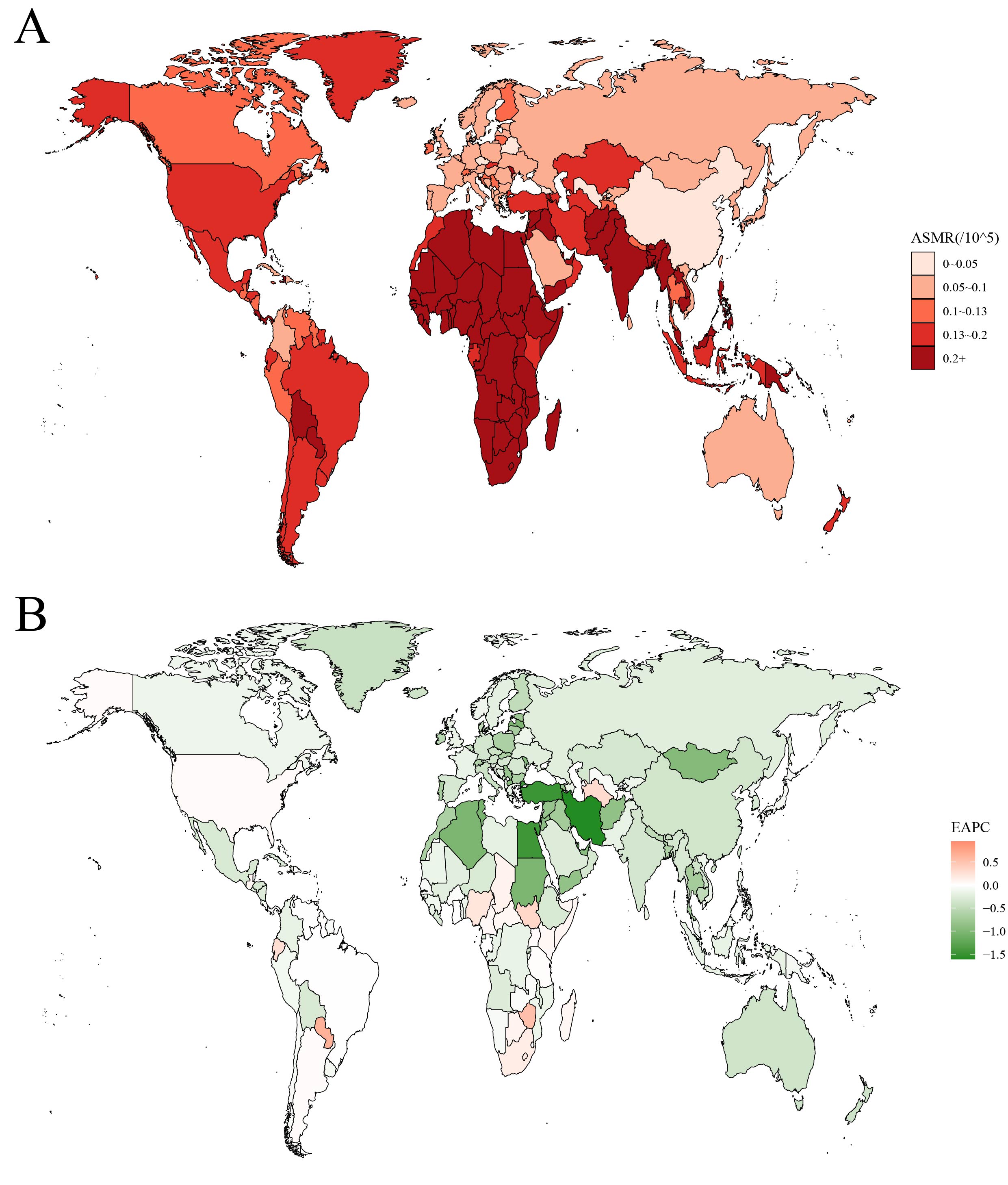


Figure S1. The global disease burden of congenital musculoskeletal and limb anomalies for both sexes in 204 countries and territories. (A) The ASMR of congenital musculoskeletal and limb anomalies in 2021; (B) The EAPC of congenital musculoskeletal and limb anomalies ASMR from 1990 to 2021. ASMR, age standardized mortality rate; EAPC, estimated annual percentage change.


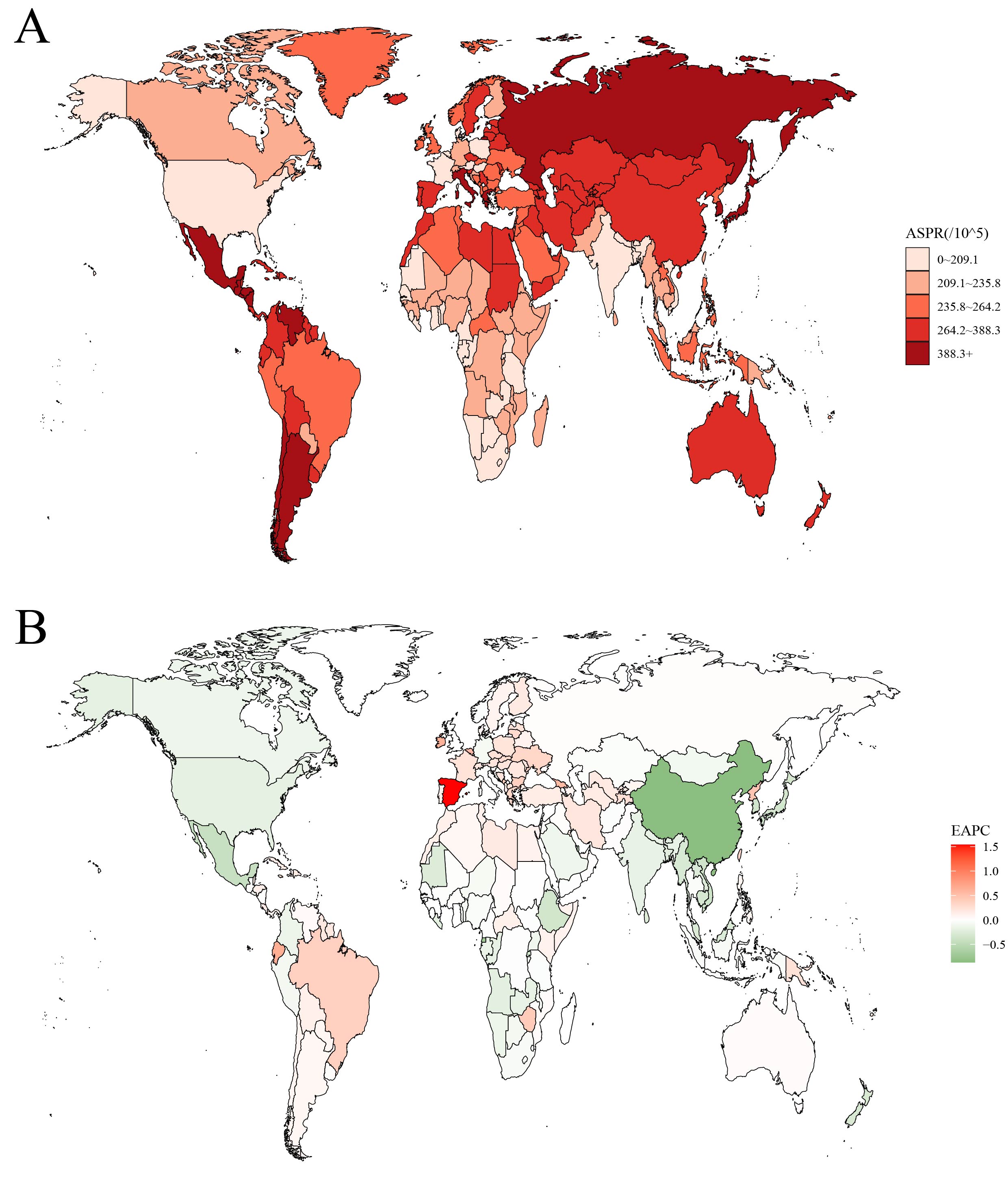


Figure S2. The global disease burden of congenital musculoskeletal and limb anomalies for both sexes in 204 countries and territories. (A) The ASPR of congenital musculoskeletal and limb anomalies in 2021; (B) The EAPC of congenital musculoskeletal and limb anomalies ASPR from 1990 to 2021. ASPR, age standardized prevalence rate; EAPC, estimated annual percentage change.
